# Supplementary material for: Parent-of-origin effects on complex traits in up to 236,781 individuals
Source: Nature. 2025 Aug 6;646(8085):647–56. doi: 10.1038/s41586-025-09357-5 (PMC12527933; doi:10.1038/s41586-025-09357-5)
Supplement: Supplementary file 1 — Supplementary Notes 1–20, Supplementary Figures 1–23 and Supplementary Tables 1–12 [file 41586_2025_9357_MOESM1_ESM.pdf]

nature portfolio

https://doi.org/10.1038/s41586-025-09357-5

Supplementary information

Parent-of-origin effects on complex traits in up to 236,781 individuals

In the format provided by the authors and unedited

Supplementary Information:
Parent-of-origin effects on complex traits in up to 236,781 individuals

Robin J. Hofmeister[1,2,3,4,✉], Théo Cavinato[1,2], Roya Karimi[5], Adriaan van der Graaf[1,2], Fanny-Dhelia Pajuste[4], Jaanika Kronberg[4], Nele Taba[4], Estonian Biobank research team[*], Reedik Mägi[4], Marc Vaudel[5,6], Simone Rubinacci[7], Stefan Johansson[5,8], Lili Milani[4,9], Olivier Delaneau[10], and Zoltán Kutalik[1,2,3,✉]

[1]Department of Computational Biology, University of Lausanne, Lausanne, Switzerland, [2]Swiss Institute of Bioinformatic (SIB), University of Lausanne, Lausanne, Switzerland, [3]University Center for Primary Care and Public Health, Lausanne, Switzerland, [4]Estonian Genome Centre, Institute of Genomics, University of Tartu, Estonia , [5]Mohn Center for Diabetes Precision Medicine, Department of Clinical Science, University of Bergen, Bergen, Norway, [6]Department of Genetics and Bioinformatics, Health Data and Digitalization, Norwegian Institute of Public Health, Oslo, Norway, [7]Institute for Molecular Medicine Finland, Helsinki, Finland, [8] Department of Pediatrics, Haukeland University Hospital, Bergen, Norway, [9]Estonian Biobank, Institute of Genomics, University of Tartu, Estonia, [10]Regeneron Genetics Center, Tarrytown, NY, USA, ✉ Corresponding authors: Robin J. Hofmeister and Zoltán Kutalik

This document contains Supplementary Notes, Supplementary Figures and Supplementary Tables.

✉ Corresponding authors: Robin J. Hofmeister and Zoltán Kutalik

Table of Content: Supplementary Notes

- Supplementary Note 1: Comparison of intra-chromosomal and inter-chromosomal phasing
- Supplementary Note 2: Comparison between our current and previous Parent-of-Origin inference methods
- Supplementary Note 3: Impact of parent-of-origin inference errors and missingness
- Supplementary Note 4: Challenges in replicating Parent-of-Origin Effects on birth weight
- Supplementary Note 5: POE enrichment in growth- and metabolism-related traits
- Supplementary Note 6: Detailed description of Parent-of-origin association within imprinted regions
- Supplementary Note 7: Detailed description of Parent-of-origin association within additively associated regions
- Supplementary Note 8: Detailed description of Parent-of-origin association genome-wide
- Supplementary Note 9: Evidence of sex-specific bi-polar POE on glucose levels
- Supplementary Note 10: Parent-of-origin effects in early life
- Supplementary Note 12: Detailed Exploration of Parent-of-Origin protein Quantitative Trait Loci (POE-pQTLs)
- Supplementary Note 11: Detailed Exploration of Parent-of-Origin SNP Heritability
- Supplementary Note 13: Parent-of-Origin inference in the Estonian Biobank
- Supplementary Note 14: Parent-of-origin inference quality
- Supplementary Note 15: Parental rearing effect as confounder
- Supplementary Note 16: Power to detect POEs
- Supplementary Note 17: Multi-trait integration reveals broader parental contributions
- Supplementary Note 18: Evolutionary insights supporting the parental conflict hypothesis
- Supplementary Note 19: Protein QTLs, a new frontier for POEs
- Supplementary Note 20: Replication analyses support our novel findings
- Supplementary Note 21: Validation of Linkage Disequilibrium (LD) equivalence in Parent-of-Origin Effects and its applications to heritability and colocalization analyses

✉ Corresponding authors: Robin J. Hofmeister and Zoltán Kutalik

Table of Content: Supplementary Figures

- Supplementary Figure 1: Relatedness inference and clustering of close relatives in the UK Biobank.
- Supplementary Figure 2: Validation cohort structure and distribution of surrogate parents.
- Supplementary Figure 3: Inter-chromosomal phasing from close relative groups.
- Supplementary Figure 4: Illustration of errors in statistical methods.
- Supplementary Figure 5: Phasing error rates.
- Supplementary Figure 6: Chromosome X IBD analysis for parental side assignment in males.
- Supplementary Figure 7: Parental side assignment using mtDNA MVS in second-degree relatives.
- Supplementary Figure 8: Parental side assignment using mtDNA MVS in third-degree relatives.
- Supplementary Figure 9: Parental side assignment using mtDNA MVS in fourth-degree relatives.
- Supplementary Figure 10: Summary of mtDNA MVS predictors and parental assignment probabilities.
- Supplementary Figure 11: Evaluation of sibling scores in inter- and intra-chromosomal phasing.
- Supplementary Figure 12: Validation and derivation of parent-of-origin probabilities using sibling scores from inter-chromosomal phased data.
- Supplementary Figure 13: Derivation of parent-of-origin probabilities using sibling scores from intra-chromosomal phased data.
- Supplementary Figure 14: Selected parent-of-origin predictors and error rate.
- Supplementary Figure 15: Overview of inter-chromosomal phasing and parent-of-origin inference yield in the UK Biobank cohort.
- Supplementary Figure 16: Classification and detection of parent-of-origin effects in GWAS.
- Supplementary Figure 17: Overview of relatedness metrics and inter-chromosomal phasing in the Estonian Biobank (EstBB), with comparisons to the UK Biobank (UKBB).
- Supplementary Figure 18: Parental side assignment from chromosome X IBD analysis in male individuals in the Estonian Biobank.

✉ Corresponding authors: Robin J. Hofmeister and Zoltán Kutalik

- Supplementary Figure 19: Evaluation of sibling scores from intra- and inter-chromosomal phasing in the Estonian Biobank validation cohort.
- Supplementary Figure 20: Validation and derivation of parent-of-origin probabilities using sibling scores from inter-chromosomal phased data in the Estonian Biobank.
- Supplementary Figure 21: Parent-of-origin probability distribution and error rates in the Estonian Biobank.
- Supplementary Figure 22: Overview of inter-chromosomal phasing and parent-of-origin inference yield in the Estonian Biobank cohort.
- Supplementary Figure 23: Haplotype-based Linkage disequilibrium.

✉ Corresponding authors: Robin J. Hofmeister and Zoltán Kutalik

Table of Content: Supplementary Tables

- Supplementary Table 1: UK Biobank traits selected for this study.
- Supplementary Table 2: Replication of previously published parent-of-origin effects.
- Supplementary Table 3: Imprinted regions lifted over to hg19.
- Supplementary Table 4: Replication of standing height associations with sitting height.
- Supplementary Table 5: Parent-of-origin effects within additively associated regions, per phenotype.
- Supplementary Table 6: Parent-of-origin effects genome-wide.
- Supplementary Table 7: Replication of associations with early childhood traits in the UK Biobank.
- Supplementary Table 8: Number of individuals per BMI time point in the MoBa cohort.
- Supplementary Table 9: Replication of associations with infancy and childhood BMI in the MoBa cohort.
- Supplementary Table 10: Replication of associations with infancy and childhood height in the MoBa cohort.
- Supplementary Table 11: Sex-specific of parent-of-origin effects.
- Supplementary Table 12: Replication of parent-of-origin effects in the Estonian Biobank cohort.
- Supplementary Table 13: Summary of POEs replication.

✉ Corresponding authors: Robin J. Hofmeister and Zoltán Kutalik

Supplementary Notes

Supplementary Note 1 Comparison of intra-chromosomal and inter-chromosomal phasing

Traditional phasing methods[1] focus on resolving haplotypes within individual chromosomes (referred to as "intra-chromosomal phasing"). While these methods are highly effective at reducing phasing errors within chromosomes, they do not leverage information from haplotype sharing across relatives, which can provide critical additional information for phasing accuracy. In contrast, our inter-chromosomal phasing approach integrates identity-by-descent (IBD) sharing with surrogate parents across all autosomes, allowing us to assign alleles inherited from the same parent to a consistent parental haplotype across the genome. This does not only reduce phasing errors but also ensures that parental haplotypes remain correctly segregated, which is particularly crucial for parent-of-origin inference.

To quantify the benefit of inter-chromosomal phasing, we assessed phasing accuracy in a subset of 548 individuals from the UK Biobank who had both (i) close relatives allowing inter-chromosomal phasing and (ii) both parental genomes available, enabling us to directly measure phasing errors accurately. In this subset, we compared the traditional intra-chromosomal phasing against our inter-chromosomal phasing approach:

- Using intra-chromosomal phasing, the average switch error rate (SER) was 0.15%.
- Using inter-chromosomal phasing, the average SER was 0.14%.

Although the overall reduction in SER is small, the key advantage of inter-chromosomal phasing lies in the specific types of errors it corrects: it resolves long-range misphasing, where large haplotype segments are incorrectly assigned to parental haplotypes (Supplementary Figure 5A). These errors are particularly problematic for PofO inference, as they can lead to systematic misclassification of maternal and paternal alleles. For example, a single phasing error can result in half of the chromosome being incorrectly phased. While this has a relatively small impact on the overall SER, it results in 50% of alleles assigned to the incorrect parental haplotype.

By integrating inter-chromosomal phasing, we observed a substantial improvement in haplotype accuracy at the genome-wide scale (Supplementary Figure 5B,C):

- With intra-chromosomal phasing, approximately 6.24% of the genome is incorrectly phased (computed as the proportion of the genome in centimorgan).
- With inter-chromosomal phasing, this proportion was reduced to 3.82%.
- When restricting to chromosomes where inter-chromosomal phasing actively corrected misphased segments (i.e., those with at least one IBD-sharing relative), the error rate dropped further to 2.53%.

✉ Corresponding authors: Robin J. Hofmeister and Zoltán Kutalik

In addition, inter-chromosomal phasing produced longer haplotypes than intra-chromosomal phasing, reflecting a larger distance between two phasing errors (Supplementary Figure 5D,E); a higher proportion of individuals were perfectly phased using inter-chromosomal phasing compared to intra-chromosomal phasing (Supplementary Figure 5F), further highlighting its accuracy in reconstructing long, correctly phased haplotype segments.

This improvement is particularly critical for accurate PofO inference, where even minor errors in phasing, such as a single error in the middle of a chromosome, can introduce substantial misclassifications in maternal *vs.* paternal alleles. Given that our PofO assignment is based on the phased segregation of haplotypes, ensuring that entire haplotype blocks are correctly assigned to a single parent significantly improves the reliability of our method. Therefore, even though the absolute reduction in SER may seem small, the elimination of long-range phasing errors enhances the robustness of PofO inference, preventing systematic misclassifications of alleles and ensuring higher accuracy in downstream POE analyses.

It is also important to note that a significant portion of the SER calculation reflects genotyping errors, which appear as short misphased haplotype segments. The length of these segment depends on the distance to the next heterozygous site.

Supplementary Note 2 Comparison between our current and previous Parent-of-Origin inference methods

Here, we provide a detailed comparison between our novel approach and our previous parent-of-origin inference method[2], and highlight three key methodological differences.

2.1 Phasing Strategy and Sample Size Trade-Off

First, our previous approach incorporated a phasing sampling strategy, wherein we performed 1,000 iterations of inter-chromosomal phasing. This allowed us to estimate phase certainty at each variant site, defined as the proportion of runs in which a given variant was assigned to the same haplotype. We then filtered out variants with low phasing confidence (< 0.7), thereby reducing inference errors. However, this approach had several drawbacks: it was computationally demanding, complex to implement, and substantially reduced the coverage of PofO inference, with on average only 80% of an individual's allele assign to a PofO. In contrast, the current method omits this filtering step, leading to a slightly higher error rate but a significantly increased per-variant sample size. This trade-off improves association signal detection and makes the method more practical for application to other biobanks. Specifically, we estimated our increased power by estimating the effective sample size from (i) the sample size, (ii) the error rate, and (iii) the call rate, as detailed in Supplementary Note 3.

Using the estimates from our previous and current approaches, we move from a 0.51% error rate in 26,393 (with a 74.5% call rate) to a 2.05% error rate in 109,385 white British individuals (with a 100% call rate). As a result, the effective sample size increases from $(0.745 \cdot 26,393) \cdot (1 - 2 \cdot 0.0051)^2 = 19,263.71$ to $(1 \cdot 109,385) \cdot (1 - 2 \cdot 0.0206)^2) = 100,557.4$.

✉ Corresponding authors: Robin J. Hofmeister and Zoltán Kutalik

This substantial gain in effective sample size demonstrates that the trade-off between error rate and sample size ultimately strengthens the power of our analyses.

2.2 Expanding Inference to More Distant Relatives

Second, our previous method was restricted to third-degree relatives, whereas the current approach extends PofO inference to fourth-degree relatives. This change enhances the overall sample size, with 29.9% of individuals with inferred PofO having only fourth-degree relative(s) available. However, increasing relatedness distance also introduces greater uncertainty due to shorter IBD segments, likely leading to a decrease in accuracy. Additionally, the inclusion of more distant relatives increases the likelihood of undetected ancestral consanguinity, which could further affect inference accuracy.

2.3 Integration of Additional Parent-of-Origin Predictors

Third, despite these methodological differences, the key distinction between the two approaches lies in the PofO predictors used. The previous method inferred PofO using only chromosome X data, whereas the current method integrates additional predictors, including mtDNA and sib-score information. To assess the concordance of these predictors, we reanalyzed our data using only chromosome X to predict the maternal side of surrogate parents, replicating our prior study's conditions. We present these results below and compare them to our novel approach.

Using the validation cohort, we found that no paternal relatives shared a haplotype segment larger than 11.3cM, and 99% of them shared only segments smaller than 2.8 cM. We therefore used a lenient threshold and defined maternal surrogate parents as sharing a haplotype segment larger than 2.8cM. In our validation cohort, we found that 65.5% of maternal relatives met this criterion.
We next applied this criterion to identify surrogate mothers for the remaining male of the UK Biobank (i.e those not included in the validation cohort). Among the surrogate mothers we could infer using this approach, 5,809 were related to the second-degree, 26,383 third-degree, and 59,610 fourth-degree. For these target-relative pairs, we compared the maternal side inferred from chromosome X, to the parental side inferred using mtDNA MVS. The results showed that chromosome X and mtDNA predictions were concordant in 97.5% of second-degree, 96.7% of third-degree, and 92.0% of fourth-degree pairs.
Additionally, we used chromosome X to identify surrogate mothers for 4,342 individuals who also had at least one sibling. We compared chromosome X-based assignment to the sibling-score approach, and found a 90.0% agreement.

The previous concordance rate compared chromosome X assignment to each of our additional predictors individually. However, our novel approach selects the most accurate predictor at the individual level rather than applying a uniform method to all cases. To directly compare the chromosome X-only approach with our multi-predictor method, we analyzed 45,056 individuals where maternal side was inferred using chromosome X (cM > 2.8) and at having at least one additional predictor. For 20,660 individuals, chromosome X was the most accurate predictor and remained the sole determinant of maternal side. For the remaining

✉ Corresponding authors: Robin J. Hofmeister and Zoltán Kutalik

individuals, chromosome X and the additional predictor indicated the same parental side in 97% of cases. Notably, 56.1% of inconsistencies were attributed to mtDNA minor variant sharing in fourth-degree relatives.

Supplementary Note 3 Impact of parent-of-origin inference errors and missingness

Let X be a random variable taking the value of -1 if the coded allele was inherited from the father and +1 if the same allele was inherited from the mother, similar to our data for the differential POE GWAS. In a sample of n heterozygous individuals, we expect to see the value +1 and -1 half of the time. When we infer this variable, let Z denote the estimate for the true value X. We are assumed to make mistakes and flip the origin with probability π, referred to as the error rate. These mistakes happen randomly, in the sense that it is expected that half of the time we flip a -1 to a +1 and half of the time the opposite. Thus, $E[X] = E[Z] = 0$. The variance of these random variables is $Var(X) = Var(Z) = E(X^2) - E^2(X) = E(X^2) = 1$. We first compute the correlation between X and Z:

$$cor(X,Z) = \frac{cov(X,Z)}{\sqrt{Var(X)\cdot Var(Z)}} = cov(X,Z) = E((X-E(X))\cdot(Z-E(Z))) \quad (1)$$

$$= E(X\cdot Z) = \pi\cdot(-1) + (1-\pi)\cdot 1 = 1-2\pi \quad (2)$$

Since the effective sample size equals the actual sample size multiplied by the squared correlation between the true and the noisy variable, with sample size n, at 1% error rate the effective sample size is $n\cdot(1-2\cdot 0.01)^2 \approx .96\cdot n$, while at 2% error rate the effective sample size is $n\cdot(1-2\cdot 0.02)^2 \approx .92\cdot n$. Thus, if we can increase the sample size by only 5% at the cost of increasing the error rate from 1% to 2%, we already increase statistical power to detect true associations.

Supplementary Note 4 Challenges in replicating Parent-of-Origin Effects on birth weight

Despite successfully replicating previously reported POEs for multiple traits, none of the eight POEs on birth weight identified in an earlier study were replicated in the UK Biobank dataset. Several factors likely contributed to this result.

4.1 Differences in birth weight measurement accuracy

In the original Icelandic study that identified POEs on birth weight[3], birth weight data were obtained from maternal reports of their offspring's birth weight. In contrast, UKB participants self-reported their own birth weight via touchscreen questionnaire, often decades after birth, which can be considered a less accurate measurement than maternal reports. This introduces potential recall bias and random measurement error, which can dilute true genetic effects, particularly for POEs that tend to have smaller effect sizes compared to additive genetic effects.

✉ Corresponding authors: Robin J. Hofmeister and Zoltán Kutalik

4.2 Lack of Gestational Age Adjustment

Gestational age is a major determinant of birth weight, as preterm infants typically weigh less than full-term infants. When gestational age is known, it can be included as a covariate in genetic analyses to help isolate genetic effects on fetal growth. However, in the UKBB cohort, participants were not asked to report their gestational age at birth, making it impossible to account for this key factor in our analysis.

The impact of this limitation is supported by previous studies. The original Icelandic study[3] adjusted for gestational age in their birth weight GWAS. However, in their replication efforts using the UKBB data, they explicitly noted that gestational age was not available for most individuals and could not be adjusted for.

4.3 Evidence of POEs influence from SNP heritability estimates

Although we did not replicate individual POEs on birth weight, we observed non-zero POE SNP heritability (h^2_{POE}) for birth weight. Furthermore, we detected nominally significant differences between maternal and paternal contributions to SNP heritability. These findings suggest that POEs influence birth weight, but the limitations of the UKBB birth weight data likely reduced our ability to detect POEs at the single-variant level.

Supplementary Note 5 POE enrichment in growth- and metabolism-related traits

We investigated whether POEs within imprinted regions were significantly enriched in growth- and metabolism-related traits. For this, we classified the 59 traits analyzed in this study into three broad categories: growth (13 traits), metabolism (22 traits), and other (24 traits). Within imprinted regions, we identified 12 POEs linked to growth traits, 13 to metabolic traits, and only 2 to traits classified as ”other”. For comparison, we conducted a parallel analysis of additive genetic effects using the same set of variants, trait definitions, and significance threshold as for POEs. This analysis yielded 76 additive associations in imprinted regions, of which 22 were for growth traits, 31 for metabolic traits, and 23 for other traits. We compared these proportions to the ones obtained for POEs scans, which revealed a significant overrepresentation among POEs (odds ratio = 5.35, p-value = 0.018). This enrichment suggests that POEs within imprinted regions disproportionately influence traits related to growth and metabolic regulation, consistent with evolutionary theories such as the parental conflict hypothesis.

Supplementary Note 6 Detailed description of Parent-of-origin association within imprinted regions

We detailed here the POE associations identified within imprinted regions. We refer to POEs as ”significant” when then passed the phenome-wide imprinted region-focused significance threshold ($0.05/(16,574 \times 48)$. We refer to POEs as ”suggestive” when then passed the imprinted region-focused significance threshold, uncorrected for the number of trait tested ($0.05/16,574$).

✉ Corresponding authors: Robin J. Hofmeister and Zoltán Kutalik

6.1 7q32.2 revealed novel bi-polar dominant effects on lipid-related profiles

We identified a novel significant POE of rs62471721 on triglycerides (TG) and two novel suggestive POEs of rs4731690 and rs10239342 on HDL-cholesterol (HDL-C) and sex hormone-binding globulin (SHBG) levels, respectively ($P_D = 6.5 \times 10^{-9}, 1.9 \times 10^{-6}$ and 1.8×10^{-6}, respectively, Table 1). These variants are in moderate to high LD ($r^2 > 0.4$) with each other, but are not in LD with other previously reported POE associations ($r^2 < 0.001$). These novel POEs revealed clear bi-polar dominant effects, where parental alleles influence traits in opposite directions (Figure 2, Figure 3).

We found the maternal A allele of rs62471721, which is an eQTL for the imprinted genes *KLF14*, *MEST*, *COPG2* and *CPA4* in adipose tissue[4], to be associated with increased, and the paternal A allele with decreased TG levels ($\widehat{\beta}_P = -0.023$, $\widehat{\beta}_M = 0.033$, $P_P = 9.7 \times 10^{-4}$, $P_M = 1.5 \times 10^{-6}$, Figure 3B).

We also identified a bi-polar dominant POE of rs4731690 on HDL-cholesterol levels, a variant also influencing *MEST*, *COPG2* and *CPA4* expression levels in adipose tissue[4]. Specifically, we found the paternal G allele to be associated with increased, but the maternal G allele associated with decreased HDL-C levels ($\widehat{\beta}_P = 0.019$, $\widehat{\beta}_M = -0.022$, $P_P = 1.9 \times 10^{-3}$, $P_M = 2.7 \times 10^{-4}$).

Lastly, we found a bi-polar dominant POE of rs10239342, also eQTL for imprinted genes *KLF14*, *MEST* and *COPG2*[4], on SHBG levels, with the paternal G allele associated with increased, but the maternal G allele associated with decreased SHBG level ($\widehat{\beta}_P = 0.022$, $\widehat{\beta}_M = -0.024$, $P_P = 1.3 \times 10^{-3}$, $P_M = 4.1 \times 10^{-4}$).

We evaluated the independence of these effects using co-localization analysis, which found that all three traits (HDL-C, TG and SHBG) likely share the same causal POE variant (posterior probability $H_4 > 0.99$ for HDL-TG and SHBG-TG, $H_4 = 0.75$ for HDL-SHBG).

6.2 Novel maternal effects on hip circumference at 7q32.2

We discovered a novel suggestive POE of rs6467315 on hip circumference, a variant eQTL for the maternally expressed gene *KLF14* in multiple tissues, located within the 7q32.2 imprinted region (Table 1, Extended data Figure 1A). We found the maternal G allele of rs6467315 associated with increased hip circumference and we observed no effect of the paternal G allele ($\widehat{\beta}_M = -0.042$, $\widehat{\beta}_P = 0.001$, $P_M = 5.8 \times 10^{-11}$, $P_P = 0.9$, $P_D = 2.2 \times 10^{-6}$, Extended data Figure 1B). The variant is not in LD with the previous POEs we reported on TG, HDL-C and SHBG above ($r^2 < 0.001$), but is in high LD ($r^2 = 0.96$) with other variants exhibiting maternal effects on TG, HDL-C, and T2D reported by previous studies (see replication section, Supplementary Table 2)[5,6]. Consistently, we found moderate associations when testing rs6467315 for POEs on these traits ($P_D = 6.9 \times 10^{-3}$, 8.9×10^{-4} and 4.6×10^{-4} for T2D, TG and HDL-C, respectively), with maternal effects aligning to those previously reported. For example, we found the maternal G allele of rs6467315 to be associated with increased T2D prevalence, while the paternal G allele had no effect ($\widehat{\beta}_M = 0.088$, $\widehat{\beta}_P = -0.010$, $P_M = 6.6 \times 10^{-4}$, $P_P = 0.69$).

Also at 7q32.2, the conditional analysis allowed us to discover a novel suggestive POE, rs3847104, independent of rs6467315 ($r^2 = 0.012$), located only 62kb away from it (Extended

✉ Corresponding authors: Robin J. Hofmeister and Zoltán Kutalik

data Figure 1C). The variant was also not in LD with previous POEs we reported on TG, HDL-C and SHBG above ($r^2 < 0.001$). We found the maternal A allele of rs3847104 associated with an increase in hip circumference and did not observe an effect of the paternal G allele ($\widehat{\beta}_{M_c} = 0.05$, $\widehat{\beta}_{P_c} = -0.016$, $P_{M_c} = 1.9 \times 10^{-08}$, $P_{P_c} = 0.1$, $P_{D_c} = 3.8 \times 10^{-7}$, Extended data Figure 1D).

6.3 A complex pattern of bi-polar and maternal dominant effect on standing height at 11p15.5

We detected three independent significant loci associated with standing height in a parent-of-origin dependent fashion at the *H19*/*IGF2* imprinted region (Table 1, Figure 2, Extended data Figure 2). Two of these, rs143840904 and rs77708343, are in high LD ($r^2 > 0.6$) with variants that were associated with height in previous POE studies[7,8,9] (see replication section, Extended data Figure 2F,G).
At the same locus, the conditional analysis allowed us to discover an independent and novel significant POE of rs576603, located more than 120kb away and not in LD with both previously reported lead SNPs ($r^2 < 0.003$ with rs77708343 and rs143840904, respectively). The paternal T allele of this variant is associated with decreased height, while the maternal one had no effect ($\widehat{\beta}_P = -0.027$, $\widehat{\beta}_M = 0.005$, $P_P = 8.53 \times 10^{-11}$, $P_M = 0.2$, $P_D = 3.86 \times 10^{-8}$, Extended data Figure 2A,E). The variant is a splicing QTL (sQTL) for the maternally expressed *H19* gene in >25 GTEx tissues and also acts as an eQTL for the paternally expressed gene *IGF2* in tibial artery[4]. Further conditional analyses showed that all three variants were independently associated with standing height ($P_{D_c} = 3.86\times10^{-8}$, 3.88×10^{-10} and 5.8×10^{-13} for rs576603, rs77708343 and rs143840904, respectively).
To understand how these loci can differentially impact various growth parameters, we evaluated these variants for POEs on sitting height. For rs143840904 and rs77708343, the associations showed similar (and significant) trends for sitting height (see Supplementary Table 4). Interestingly, while the significance and effect sizes (in SD) of rs143840904 decreased for sitting height, the association of rs77708343 remained relatively stable, indicating that it may contribute equally to trunk size as it does to overall height. On the other hand, rs576603 did not associate with sitting height in a similar fashion, suggesting that this variant likely contributes to femoral length, rather than to trunk size.

6.4 Bi-polar dominant effects on metabolic traits at 11p15.5

At the same standing-height associated locus, we also uncovered suggestive bi-polar POEs on metabolic rate and body composition traits (Table 1, Figure 2). Specifically, the paternal G allele of the standing height-associated SNP rs77708343 (see above) was also associated with a decrease in metabolic rate, while the maternal G allele showed an increase ($\widehat{\beta}_P = -0.048$, $\widehat{\beta}_M = 0.035$, $P_P = 1.2 \times 10^{-5}$, $P_M = 1.3 \times 10^{-3}$, $P_D = 7.5 \times 10^{-8}$). Given that basal metabolic rate is estimated from height and weight, we further examined the effects of rs77708343 on weight, and found the paternal G allele associated with decreased, and the maternal G allele associated with increased weight ($\widehat{\beta}_P = -0.064$, $\widehat{\beta}_M = 0.031$, $P_P = 1.3 \times 10^{-5}$, $P_M = 3.1 \times 10^{-2}$, $P_D = 3.8 \times 10^{-6}$).

✉ Corresponding authors: Robin J. Hofmeister and Zoltán Kutalik

Due to the correlation between height and weight, we further explored associations of rs77708343 with additional morphological traits, including waist and hip circumference, as well as fat, fat-free and water mass measurements for legs, arms, trunk and whole body. The analysis revealed that rs77708343 influenced waist circumference, fat-free mass and whole body water mass, while no significant effect was observed on hip circumference or fat percentage. Specifically, the paternal G allele was associated with decreased and the maternal G allele with increased trait levels ($\widehat{\beta}_P = -0.056$, -0.052, -0.032, -0.038 and -0.043, $\widehat{\beta}_M = 0.039$, 0.032, 0.030, 0.034 and 0.033, $P_P = 1.0 \times 10^{-3}$, 1.2×10^{-6}, 1.8×10^{-3}, 1.9×10^{-4} and 1.0×10^{-5}, $P_M = 1.7 \times 10^{-2}$, 2.0×10^{-3}, 3.1×10^{-3}, 8.0×10^{-4} and 1.2×10^{-3}, $P_D = 7.0 \times 10^{-5}$, 1.8×10^{-8}, 1.6×10^{-5}, 5.5×10^{-7} and 1.6×10^{-7} for waist circumference, leg, arm, and trunk fat-free mass, and whole-body water mass, respectively).

6.5 Novel bi-polar dominant effects on renal function indicators at 11p15.5

We identified two novel significant POEs on cystatin C and creatinine, and a novel suggestive POE on urate levels ($P_D = 5.9 \times 10^{-14}, 4.9 \times 10^{-11}$ and 2.2×10^{-7}, respectively, Table 1, Figure 2) at a locus located over 300kb away and not in LD with other known POEs on blood biomarkers ($r^2 < 0.001$). We observed bi-polar effects of lead SNPs rs170102 and rs217215 ($r^2 > 0.98$) for both cystatin C and creatinine, respectively. Specifically, we found the paternal A allele of rs170102 associated with decreased, but the maternal A allele associated with increased cystine C levels ($\widehat{\beta}_P = -0.041$, $\widehat{\beta}_M = 0.026$, $P_P = 5.9 \times 10^{-11}$, $P_M = 3.1 \times 10^{-5}$). We also found the paternal G allele of rs217215 associated with increased, but the maternal G allele associated with decreased creatinine levels ($\widehat{\beta}_P = 0.03$, $\widehat{\beta}_M = -0.024$, $P_P = 2.5 \times 10^{-7}$, $P_M = 2.6 \times 10^{-5}$). For urate, we found the G allele of rs4264135 associated with decreased urate levels when paternally inherited, and observed no effect when maternally inherited ($\widehat{\beta}_P = -0.033$, $\widehat{\beta}_M = 0.014$, $P_P = 1.8 \times 10^{-7}$, $P_M = 0.033$).

6.6 Distinct bi-polar dominant and maternal effects on blood glucose biomarkers and type 2 diabetes at the 11p15.5 region

We identified significant POEs on type 2 diabetes (T2D) and HbA1c and suggestive POE on glucose levels, at two variants in high LD ($r^2 > 0.99$), rs10838787 and rs4417225 ($P_D = 1.8 \times 10^{-9}, 2.7 \times 10^{-17}$ and 7.4×10^{-8}, respectively, Table 1, Figure 2). Specifically, we found the paternal A allele of rs10838787 to be associated with increased T2D prevalence, while the maternal A allele appears to be protective ($\widehat{\beta}_P = 0.136$, $\widehat{\beta}_M = -0.09$, $P_P = 3.8 \times 10^{-7}$, $P_M = 7.4e \times 10^{-4}$, $P_D = 1.8 \times 10^{-9}$, Extended data Figures 4A,B). We also found bi-polar dominant effects on HbA1c and glucose, with paternal alleles associated with increased, and maternal alleles associated with decreased trait levels ($\widehat{\beta}_P = 0.05$ and 0.029 , $\widehat{\beta}_M = -0.028$ and -0.028, $P_P = 2.3 \times 10^{-14}$ and 9.2×10^{-5}, $P_M = 1.8 \times 10^{-5}$ and 2.0×10^{-4} for HbA1c and glucose, respectively, Extended data Figures 4C,D).

These variants are in high LD ($r^2 > 0.9$) with rs2334499, a variant previously associated with T2D in a parent-of-origin manner[6]. To determine whether rs10838787 and rs2334499 represent independent signals, we conducted a conditional analysis. After adjusting for rs2334499, we found that rs10838787 remained nominally significant ($P_{D_c} = 0.026$), whereas

✉ Corresponding authors: Robin J. Hofmeister and Zoltán Kutalik

rs2334499 lost its association ($P_{D_c} = 0.67$). This strongly that the previously reported POE of rs2334499 was likely driven by LD with rs10838787, rather than an independent causal effect. Our results thus refine the parent-of-origin association at 11p15.5, suggesting rs10838787 as the likely causal variant driving the observed effects on T2D and HbA1c. We identified a additional, independent and suggestive POE of rs2299620 on HbA1c ($P_D = 2.2 \times 10^{-6}$), a variant located 1.1 Mb away and not in LD with the previously discussed variants ($r^2 < 0.01$, Table 1, Extended data Figure 4C). The maternal T allele was associated with decreased HbA1c levels ($\widehat{\beta}_M = -0.12$, $P_M = 1.3 \times 10^{-13}$), while the paternal T allele has no detectable effect. Here again, the variant is in high LD ($r^2 = 0.5$) with rs2237892, a variant previously associated with T2D[6]. Since HbA1c is a known proxy for T2D[10], we also examined the POE of rs2299620 on T2D. Although not reaching our conventional significance threshold, we observed a moderate protective effect of the maternal T allele ($\widehat{\beta}_M = -0.36$, $\widehat{\beta}_P = -0.01$, $P_M = 3.2 \times 10^{-7}$, $P_P = 0.42$, $P_D = 2.2 \times 10^{-3}$). To disentangle the observed signal at this locus, we performed a conditional analysis for HbA1c, which revealed that the two SNPs are not independently associated with HbA1c. SNP rs2299620 emerged as the lead variant driving the observed HbA1c association at this locus ($P_{D_c} = 8.1 \times 10^{-5}$ and 0.37 for rs2299620 and rs2237892, respectively).

6.7 A novel locus with bi-polar dominant effects on body fat composition at 20q13.32

We identified a novel POEs within the 20q13.32 imprinted region, including a significant POE on arm fat percentage and several suggestive POE on diverse fat percentage traits, including body fat percentage. Specifically, we found the paternal G allele associated with increased in body fat percentage, while the maternal G allele showed the opposite effect ($\widehat{\beta}_P = 0.037$, $\widehat{\beta}_M = -0.037$, $P_P = 2.6 \times 10^{-4}$, $P_M = 2.2 \times 10^{-4}$, $P_D = 2.0 \times 10^{-7}$). To assess which parts of body fat this effect is driven by, we examined its POE on arm-, leg-, and trunk fat percentages. Significant POEs were observed for all these traits ($\widehat{\beta}_P = 0.025, 0.035$, and 0.042; $\widehat{\beta}_M = -0.024, -0.042$, and -0.039; $P_P = 2.8 \times 10^{-3}, 4.3 \times 10^{-4}$, and 3.6×10^{-4}; $P_M = 3.5 \times 10^{-3}, 2.0 \times 10^{-5}$, and 1.0×10^{-3}; $P_D = 2.9 \times 10^{-5}, 3.4 \times 10^{-8}$, and 1.3×10^{-6} for leg, arm, and trunk fat percentages, respectively). These lead variants were identified as either sQTLs for *STX16* or *CTSZ* in adipose tissue[4] or eQTLs for the *GNAS* imprinted gene in blood[11], underscoring their potential functional relevance in the observed POEs.

6.8 Novel and distinct maternal and bi-polar effects on IGF-1 at 6q24.2 and 16p13.3

We identified a novel suggestive POE of rs12528876 on IGF-1 levels at the 6q24.2 locus, located in an intron of the *PLAGL1* gene ($P_D = 2.9 \times 10^{-6}$, Table 1). Specifically, we found the paternal C allele of rs12528876 to be associated with decreased IGF-1 levels and the maternal allele having moderate opposite effect ($\widehat{\beta}_P = -0.036$, $\widehat{\beta}_M = 0.021$, $P_P = 2.5 \times 10^{-5}$, $P_M = 0.016$).

We identified a second independent, novel and suggestive POE of rs7188903 on IGF-1 at 16p13.3 ($P_D = 6.1 \times 10^{-7}$, Table 1), which is an eQTL in blood for two maternally expressed genes, *ZNF597* and *NAA60*. We found the maternal G allele of rs7188903 to be associated

✉ Corresponding authors: Robin J. Hofmeister and Zoltán Kutalik

with increased IGF-1 levels and the paternal allele having no effect ($\widehat{\beta}_M = 0.041$, $\widehat{\beta}_P = -0.012$, $P_M = 5.3 \times 10^{-8}$, $P_P = 0.1$).

6.9 Novel bi-polar dominant effect on total protein levels at 15q12

We identified a novel suggestive POE of rs146982369 on total blood protein levels within the 15q12 imprinted region, located in the intro of *ATP10A* ($P_D = 2.5 \times 10^{-6}$, Table 1). We found the paternal G allele of rs146982369 associated with an increase in blood protein levels, while the maternal G allele has the opposite effect ($\widehat{\beta}_P = 0.101$, $\widehat{\beta}_M = -0.106$, $P_P = 1.8 \times 10^{-3}$, $P_M = 9.9 \times 10^{-4}$).

6.10 A known maternal effect on platelet count at 14q32.2

Lastly, we identified a significant POE of rs59228823 on platelet count, an association that was already reported in our previous study[2]. This variant has been mapped to the *MEG3* maternally expressed gene, and is in high LD ($r^2 > 0.55$) with rs12881545, a known maternal eQTL of *MEG3*[12].

Supplementary Note 7 Detailed description of Parent-of-origin association within additively associated regions

Another way to reduce multiple testing burden in a meaningful way is to restrict our POE analysis to variants exhibiting an additive effect on the examined traits, as captured by traditional GWAS. For simplicity, we refer to these as "additively associated regions" in subsequent sections. We first performed additive association testing (ignoring the PofO of the alleles) for 59 selected traits (Supplementary Table 1) and identified 1,812 significant (additive P-value $< 1 \times 10^{-9}$, see Methods) LD-pruned additive associations. We then sought to detect whether some of these associations may involve POEs. To this end, we applied a differential POE GWAS scan (see Methods) restricted to these SNP-trait pairs. This approach is geared towards the identification of POEs where the maternal and paternal effects are not in opposite direction and hence even the simple additive effect is non-zero. While this strategy allows us to detect only a subset of POE scenarios (Supplementary Figure 16), it also drastically reduces the multiple testing burden, setting the significance threshold to $0.05/1812 = 2.75 \times 10^{-5}$ (see Methods). This analysis identified six significant POEs (Table 1, Figure 2). We provide here a more detailed description of each association.

Two of these POEs were novel associations, both with telomere length (Figure 4). First, the C allele of rs2293607 on chromosome 3, located within the *TERC* gene (exon of noncoding transcript), was found to be associated with decreased telomere length when inherited from either parent, although the effect is significantly stronger when inherited paternally ($\widehat{\beta}_P = -0.12$, $\widehat{\beta}_M = -0.068$, $P_P = 2.2 \times 10^{-55}$, $P_M = 5.3 \times 10^{-18}$, $P_D = 5.7 \times 10^{-7}$, Figure 4A,B).Furthermore, the T allele of rs11100479 on chromosome 4, an eQTL for the gene *NAF1*, was associated with an increase in telomere length when inherited from the father. We also observed a moderate effect of the maternal T allele, albeit the effect of the paternal T allele was significantly stronger ($\widehat{\beta}_P = 0.078$, $\widehat{\beta}_M = 0.022$, $P_P = 1.8 \times 10^{-22}$,

✉ Corresponding authors: Robin J. Hofmeister and Zoltán Kutalik

$P_M = 6.6 \times 10^{-3}$, $P_D = 6.25 \times 10^{-7}$, Figure 4A,C).

Two additional associations were found in moderate to low LD with previously reported variants. First, at the 5p15.33 locus, the POE of rs7705526 on telomere length exhibited moderate LD ($r^2 = 0.27$) with rs2735940, a variant previously implicated in telomere length in our earlier work[1]. This reflects the limitations of the additively associated regions approach, which assesses the POE specificity of the lead additive variant but may not always pinpoint the true lead POE variant. Indeed, rs2735940 was also identified as the lead POE variant for telomere length in our genome-wide analysis (see Supplementary Note 8, Figure 4A,D).

Second, at the 11p15.5 region, we identified maternal effect of rs2237895 on HbA1c. This variant was in low LD ($r^2 = 0.025$) with rs2299620, previously implicated in our imprinted region-focused analysis. Conditional analysis suggested that neither variant met the significance threshold independently ($P_{D_c} = 3.5 \times 10^{-4}$ and 4.3×10^{-5} for rs2237895 and rs2299620, respectively), suggesting that they may represent the same association. To avoid redundancy, we only reported rs2299620 in Table 1 and Figure 2, as it is our lead POE-variant identified in the imprinted region-focused analysis.

The remaining two associations - with standing height at 11p15.5 and with platelet count at 14q32.2 - were already identified in our imprinted region-focused analysis (see Table 1).

In less stringent analysis, where the testing burden is reduced by evaluating each trait individually, we identified six additional suggestively significant POEs (Supplementary Table 5), two of which (both novel) with IGF-1. The maternal T allele of the first lead variant rs17145750, intronic to *MLXIPL*, was associated with increased IGF-1 levels, and we observed no effect from the paternal T allele ($\widehat{\beta}_P = 0.015$, $\widehat{\beta}_M = 0.063$, $P_P = 0.09$, $P_M = 9.07 \times 10^{-13}$, $P_D = 1.18 \times 10^{-4}$). The second association is located within the 11p15.5 imprinted region. The lead variant rs3213217, intronic to *IGF2*, demonstrated asymmetric polar effects, with both maternal and paternal T alleles associated with increased IGF-1 levels, albeit to different extents ($\widehat{\beta}_P = 0.096$, $\widehat{\beta}_M = 0.058$, $P_P = 1.4 \times 10^{-34}$, $P_M = 2.0 \times 10^{-13}$, $P_D = 5.7 \times 10^{-4}$). While these findings did not meet the stringent significance threshold when focusing on additive regions, they highlight potential POEs with moderate effects.

Supplementary Note 8 Detailed description of Parent-of-origin association genome-wide

We performed a genome-wide scan for parent-of-origin effects (POEs) across 59 selected traits (Supplementary Table 1) and identified six significant POE associations (differential P-value $P_D < 1 \times 10^{-9}$, see Methods, Table 1, Figure 2, Supplementary Table 6A). All of these associations had already been detected in our analyses focused on imprinted regions or additively associated regions (or were in LD with previously identified lead variants), and therefore did not represent additional independent signals. However, because the lead POE variant can differ from the lead additive variant, this analysis allowed us to refine the maternal-only effect on telomere length at the *TERT* locus, where we identified rs2735940 as the likely POE-driving variant, consistent with our earlier findings[2].

✉ Corresponding authors: Robin J. Hofmeister and Zoltán Kutalik

By reducing the testing burden and analyzing each trait independently, we identified 11 additional suggestively significant POEs (differential P-value $P_D < 5 \times 10^{-8}$). Of these, 4 overlapped with findings from the targeted strategies (for triglycerides, T2D, leg fat-free mass, and arm fat percentage), while the remaining 7 were novel (Supplementary Table 6B). For example, we found a novel suggestive POE of rs145976519 on birth weight. This variant, located at 1p34.2, lies outside known imprinted regions and shows no additive association with birth weight. The C allele was associated with decreased birth weight when inherited maternally, while the paternal C allele increased it. Beyond its role as an eQTL for *CAP1* and *PPT1*, rs145976519 is located approximately 130kb from *ZMPSTE24* and modulates the expression of its divergent transcript[11]. Notably, *ZMPSTE24* has been implicated in neonatal pathologies, including intrauterine growth retardation and prematurity.

Supplementary Note 9 Evidence of sex-specific bi-polar POE on glucose levels

Recognizing that complex traits often exhibit sex-specific variation[13,14], we investigated whether POEs also display sex-specific patterns. To explore this, we conducted POE analyses separately for males and females across the 30 POE associations identified in this study and listed in Table 1.

We identified one significant sex-specific difference, for the POE of rs4417225 (near *H19*/*IGF2*) on glucose levels (Supplementary Table 11, Extended data Figure 5). The POE was significant in male individuals ($P_{D_{males}} = 4.7 \times 10^{-9}$), but not in female individuals ($P_{D_{females}} = 0.19$). Specifically, the T allele of rs4417225 was associated with increased glucose levels in men when paternally inherited, and with decreased levels when maternally inherited ($\widehat{\beta}_P = 0.045$, $\widehat{\beta}_M = -0.040$, $P_P = 1.0 \times 10^{-4}$, $P_M = 1.3 \times 10^{-5}$), while we observed no effect of neither paternal nor maternal T alleles in women ($P_P = 0.3$, $P_M = 0.38$). Additionally, we formally tested for significant sex differences in POE by comparing male and female GWAS effect sizes using a Z-score approach, applying a significance threshold of $P_Z < 0.05/30 = 1.67 \times 10^{-3}$ (see Methods). This analysis confirmed a significant sex-specific POE for glucose levels ($P_{Z_D} = 1.3 \times 10^{-3}$, Supplementary Table 11). Interestingly, although this locus is also associated with HbA1c and type 2 diabetes risk in a POE fashion, no sex-specific effects were detected for these traits (Supplementary Table 11).

Supplementary Note 10 Parent-of-origin effects in early life

We examined the timing of POEs on complex traits to assess whether these effects manifest in early childhood or are specific to adult traits. For this purpose, we first tested three loci associated with standing height for significant POEs on ”comparative height size at age 10” in the UKBB and on infant height in the Norwegian Mother, Father and Child Cohort Study (MoBa). Additionally, we tested seven loci linked to obesity-related traits for POEs on birth weight and ”comparative body size at age 10” in the UKBB, and on infant BMI in MoBa. We determined significant associations as having a POE differential p-value $P_D < 7.4 \times 10^{-4}$ (see Methods).

✉ Corresponding authors: Robin J. Hofmeister and Zoltán Kutalik

10.1 Comparative childhood traits at age 10 in the UK Biobank

In the UK Biobank, we found two loci associated with adult standing height also exhibiting significant POEs on "comparative height size at age 10" ($P_D < 7.4 \times 10^{-4}$, see Methods, Supplementary Table 7, Extended data Figure 6A). Specifically, the paternal T allele of rs576603, our novel height-associated POE, and the paternal G allele of rs77708343, both located near *IGF2*, were linked to reduced height at age 10, whereas their maternal counterparts were associated with increased height. These findings mirror the POE effects observed in adult standing height, suggesting that these loci may influence early growth trajectories with long-term effects.

Moreover, despite being in low LD ($r^2 = 0.003$) but in close proximity ($\approx 120kb$), we confirmed that these two variants independently affected height at age 10, consistent with their impact on adult height ($P_{D_c} = 1.4 \times 10^{-5}$ and 1.8×10^{-4} for rs576603 and rs77708343, respectively).

10.2 Early-life POEs in the MoBa cohort

We additionally analyzed the same loci for association with early life height and BMI using data from the Norwegian Mother, Father and Child Cohort Study (MoBa)[15]. We used 45,402 children with both parents and longitudinal height and BMI measurements at 11 time points, from 6 weeks to 8 years of age[16] (Supplementary Table 8). Depending on the time point, up to 42,346 individuals were included in the association analyses.

10.2.1 Infant height POEs in the MoBa cohort

Two of the three standing height-associated POE variants were available for replication in MoBa: rs576603 and rs77708343. We identified significant bi-polar POEs on height only for rs77708343 ($P_D < 7.4 \times 10^{-4}$, see Methods). Specifically, we found the paternal G allele decreasing, and the maternal G allele increasing height (all time points, 6 weeks to 8 years $\widehat{\beta}_P = -0.091$ to -0.175, $\widehat{\beta}_M = 0.068$ to 0.143, $P_P = 9.1 \times 10^{-3}$ to 9.6×10^{-10}, $P_M = 2.7 \times 10^{-2}$ to 3.7×10^{-6}). The observed POEs of rs77708343 in infant were consistent with those observed in the UK Biobank, both with comparative height at age 10 and with adult height (Supplementary Table 10, Extended data Figure 6A). We further confirmed that the observed effect on infant height was not attributable to maternal untransmitted alleles in MoBa (Extended data Figure 6A, Supplementary Table 10), ruling out maternal rearing effects and supporting true imprinting.

10.2.2 Early-life BMI dynamics in the MoBa cohort

We also identified seven significant POEs on infant BMI ($P_D < 7.4 \times 10^{-4}$, see Methods), all involving the SNP rs6467315. Specifically, we found the maternal G allele of rs6467315 associated with increased BMI during infancy (6 weeks to 2 years, $\widehat{\beta}_M = 0.057$ to 0.089, $P_M = 5.9 \times 10^{-4}$ to 9.4×10^{-7}), while the paternal allele showed no effect. The maternal effect was strongest at 6 months and gradually attenuated, becoming undetectable by the age of 3 years (Supplementary Table 9, Extended data Figure 6B). This attenuation likely explains the absence of a detectable effect at age 10 in the UK Biobank cohort.

✉ Corresponding authors: Robin J. Hofmeister and Zoltán Kutalik

Interestingly, the maternal G allele had an opposite direction of effect on adult traits, where it decreased both hip circumference (see above, Table 1) and BMI ($\widehat{\beta}_M = -0.02$, $\widehat{\beta}_P = 0.006$, $P_M = 2.6 \times 10^{-4}$, $P_P = 0.33$, $P_D = 1.0 \times 10^{-3}$, Extended data Figure 6B). We performed co-localization analysis, confirming that the same causal variant likely drives both traits at this locus (posterior probability H4 > 0.96).

These findings align with earlier research on rs287621 (r^2=0.36 with rs6467315), which reported similar opposing maternal effects on BMI across infancy and adulthood[16]. Consistently, we also find a moderate maternal effect of rs287621 on hip circumference, with the maternal T allele decreasing hip circumference, and no observable effect of the paternal C allele ($\widehat{\beta}_M = -0.035$, $\widehat{\beta}_P = -0.003$, $P_M = 9.3 \times 10^{-7}$, $P_P = 0.61$, $P_D = 1.8 \times 10^{-3}$). We, however, found no POE of this variant on adult BMI in the UK Biobank cohort ($\widehat{\beta}_M = 0.016$, $\widehat{\beta}_P = 0.001$, $P_M = 0.023$, $P_P = 0.82$, $P_D = 0.14$).

To disentangle the observed signal at this locus, we performed a conditional analysis for both adult hip circumference and BMI, which revealed that our lead POE variant, rs6467315, is the one driving the observed associations at this locus (hip circumference $P_{D_c} = 3.6 \times 10^{-3}$ and 0.84; BMI $P_{D_c} = 9.7 \times 10^{-3}$ and 0.49, for rs6467315 and rs287621, respectively).

We also confirmed that the observed maternal effect on early-life BMI was not attributable to maternal untransmitted alleles (Extended data Figure 6B, Supplementary Table 9), ruling out maternal rearing effects as the underlying mechanism and consistent with imprinting effects.

Supplementary Note 11 Detailed Exploration of Parent-of-Origin SNP Heritability

While the main text highlights the overall modest contribution of h^2_{POE} to SNP-based heritability, we provide here additional details on the maternal *vs.* paternal contributions and on the enrichment of h^2_{POE} within imprinted regions.

11.1 Enrichment of h^2_{POE} in imprinted regions

To assess whether h^2_{POE} is more pronounced in known imprinted regions, we compared h^2_{POE} within and outside imprinted loci (Extended data Figure 7A). We observed nominally significant increases in h^2_{POE} within imprinted regions for six traits: IGF-1, Triglycerides, Eosinophil count, Lymphocyte count, Birth weight and basophil count.

This enrichment within imprinted regions suggests that genomic imprinting plays a disproportionate role in shaping POEs for these traits. Reassuringly, no traits exhibited higher h^2_{POE} outside imprinted regions, indicating that POEs are not randomly distributed across the genome but rather concentrated in regions known to be under imprinting control.

While h^2_{POE} remains modest overall, its trait-specific enrichment in imprinted regions and imbalances in maternal *vs.* paternal contributions suggest that parent-of-origin effects modulate key biological pathways, particularly those involved in growth, metabolism, and immune function. Future studies leveraging larger datasets and multi-omics approaches (e.g., transcriptomic and epigenomic data) will be essential to further elucidate the mechanisms

✉ Corresponding authors: Robin J. Hofmeister and Zoltán Kutalik

underlying POEs and their contribution to complex traits.

11.2 Maternal *vs.* Paternal contributions to SNP-based heritability

To further explore whether maternal and paternal haplotypes contribute differently to SNP-based heritability, we estimated $h^2_{maternal}$ and $h^2_{paternal}$ separately for each trait, both genome-wide and restricted to imprinted regions (Extended data Figure 7B,C). In most cases, maternal and paternal contributions were similar, consistent with the expectation of additive genetic effects. However, a few notable trait-specific imbalances were observed:

- Higher maternal SNP heritability: Type 2 diabetes and arm fat percentage exhibited a trend toward a greater maternal contribution, suggesting that maternally inherited alleles may have a stronger influence on these metabolic traits.
- Higher paternal SNP heritability: Birth weight, glucose, and basophil count showed a greater paternal contribution, which may reflect paternal influences on early growth and immune regulation.

These patterns align with previous findings linking genomic imprinting and differential parental contributions to metabolic and developmental traits. However, while nominally significant (P-value < 0.05), these differences in maternal *vs.* paternal h^2 estimates did not reach significance when correcting for the number of phenotype assessed, suggesting that while interesting, these effects warrant further investigation in larger datasets.

Supplementary Note 12 Detailed Exploration of Parent-of-Origin protein Quantitative Trait Loci (POE-pQTLs)

In addition to our investigation of POEs on complex traits, we explored the UK Biobank plasma proteomics data to identify parent-of-origin protein quantitative trait loci (POE-pQTLs). Our analysis was restricted to two main objectives: first, determining whether variants exhibiting POEs on complex traits identified in this study also exhibit POE-pQTL effects on their associated genes, and second, assessing whether previously established pQTLs[17] exhibit associations in a PofO fashion.

12.1 Parent-of-Origin pQTLs at known POEs

Across the genes associated with variants exhibiting POEs on complex traits identified in this study (Table 1), two genes, *CPA4* and *GNAS*, had available protein quantification data. To investigate potential POEs at the protein level, we tested eight variant-protein pairs: rs10239342, rs62471721, rs4731690, rs6467315 and rs3847104 with *CPA4*, and rs80116540, rs6026426, and rs3730173 with *GNAS*. While no significant POE-pQTLs were observed for *GNAS*, we identified one significant ($P_D < 0.05/8$) POE-pQTL for *CPA4*. Specifically, the G allele of rs4731690, previously identified as exerting a bi-polar effect on HDL-C, was found to increase *CPA4* protein levels only when inherited paternally.

✉ Corresponding authors: Robin J. Hofmeister and Zoltán Kutalik

12.2 Novel Parent-of-Origin pQTLs among additive pQTLs

We additionally evaluated a set of established additive pQTLs[17] for potential POEs. From the 14,287 lead pQTL associations spanning 2,414 proteins reported in a previous study[17], we could directly test 10,611 associations in our dataset, where the same SNP and protein data were available, and we used proxy variants selected based on LD ($r^2 > 0.8$) for the remaining ones. We identified four significant ($P_D < 0.05/14285 = 3.5 \times 10^{-6}$) POE associations, with namely *DLK1*, *CPA4*, *ADAM23* and *PER3* protein levels (Extended data Table 1, Extended data Figure 8).

We also found two POE associations where genes were located outside known imprinted regions: for *ADAM23* and *PER3* protein levels. In both cases, the paternal allele was associated with higher protein levels compared to the maternal allele. Notably, for both genes, the lead POE-pQTL differed from the lead additive pQTL reported in previous studies, with a stronger POE signal observed at a distinct variant in each case (Extended data Table 1, Extended data Figure 8C-D, Supplementary Note 12).

Supplementary Note 13 Parent-of-Origin inference in the Estonian Biobank (EstBB)

Among the 211,259 EstBB participants, we identified 14,063 parent-offspring trios, 37,686 parent-offspring duos, over 55,000 individuals with at least one sibling and more than 150,000 individuals with more distant relatives. Notably, 95% of individuals had at least one close relative (up to the fourth degree), which were then clustered in surrogate parent groups, allowing us to leverage inter-chromosomal statistical phasing via shared IBD haplotype segments for almost the entire cohort. This extensive relatedness in EstBB enabled an inter-chromosomal phasing error rate of only 0.56%, compared to 1% in the UK Biobank (Supplementary Figure 17).

For individuals without directly available parental genomes, we inferred the PofO of parental haplotype sets by analyzing chromosome X inheritance in male individuals (Supplementary Figure 18) and by using crossover inference combined with sex-specific recombination maps for sibling pairs (Supplementary Figures 19 and 20). Using this approach, we successfully inferred PofO of haplotypes in a total of 124,238 EstBB individuals, of which more than 78% (N=97,346) had a probability greater than 0.99, and achieving an average error rate at heterozygous sites of just 0.89%, compared to 2.06% in the UK Biobank (Supplementary Figure 21). We then kept only individuals for which at least one of our UKBB POE-trait was available, resulting in 96,972 individuals (Supplementary Figure 22). Depending on the phenotype, up to 85,050 individuals were included in the association analyses.

Supplementary Note 14 Parent-of-origin inference quality

While our main discussion section focused on the main advantages and limitations of our methodology, we provide here a more detailed discussion of the accuracy of our PofO inference approach. The accuracy of PofO inference depends on the availability of haplotype segments shared IBD across chromosomes, which in turn is influenced by cohort structure

✉ Corresponding authors: Robin J. Hofmeister and Zoltán Kutalik

and data availability. Sample sizes vary across chromosomes, with fewer individuals available for smaller chromosomes where detecting IBD segments is more challenging. This variability restricts the analysis power, particularly in cohorts with limited data on related individuals. This limitation is evident when comparing inferences between the UK Biobank and the Estonian Biobank. While the UK Biobank includes an average of 1.6 relatives per individual, the Estonian Biobank includes approximately 12 relatives per individual. This difference results in larger sample sizes per chromosome and reduces error rates by half in the Estonian Biobank.

Errors in PofO inference are unlikely to generate false-positive associations but primarily reduce statistical power. Because PofO errors are expected to be randomly distributed with respect to the phenotype, they should not systematically bias association results. Instead, these errors lead to dilution of true POE signals and reduce the effective sample size, as effects are misattributed to both parental origins, thereby reducing the ability to detect significant associations (see Supplementary Note 3). A similar principle applies to imputation errors: while lower imputation quality may introduce noise and reduce statistical power, stringent quality control filtering (e.g., INFO > 0.8) minimizes this risk, and any remaining imputation inaccuracies are expected to reduce power rather than induce false-positive associations. Notably, for imputation to introduce systematic errors in POE detection, imputation errors would need to be correlated with either the parent-of-origin of an allele or with the phenotype itself—an unlikely scenario given that imputation accuracy is primarily influenced by allele frequency and linkage disequilibrium rather than parental origin.

Our method is also highly dependent on the degree of relatedness among individuals. Higher accuracy and call rate is achieved with closer relatives, as seen with mtDNA minor variant sharing (MVS), where second-degree relatives can be use to predict both paternal and maternal side, versus accurately predicting only maternal side when using more distant relatives. Consequently, biobanks enriched with closer familial relationships, such as the Estonian Biobank, provide a larger proportion of the cohort with accurate PofO inference. Indeed, approximately half of the Estonian Biobank cohort had PofO probabilities exceeding 0.99, compared to only about a quarter of the UK Biobank cohort.

Another potential limitation that was previously discussed[1] relates to consanguinity within populations. Higher levels of consanguinity could lead to individuals sharing chromosome X or mtDNA from both sides of the family, potentially introducing errors in PofO assignment. However, our observations in the Estonian Biobank do not support this hypothesis. Despite significantly higher average Runs-of-Homozygosity in the Estonian Biobank compared to the UK Biobank, we observed lower error rates and better PofO inference coverage in the Estonian cohort. This is likely due to the larger number of close relatives per individual and our rigorous clustering approach for relatives. We excluded individuals for whom relatives could not be clustered into two distinct groups or for whom group clustering was ambiguous. Together, these measures ensure the robustness of our multi-step approach, even in populations with high consanguinity.

✉ Corresponding authors: Robin J. Hofmeister and Zoltán Kutalik

Supplementary Note 15 Parental rearing effect as confounder

Althoug our method has several advantages compared to trio-based studies, one limitation is that we are unable to distinguish between true imprinting effect and parental rearing, that is, the effect of parental untransmitted alleles. The effect of a maternal transmitted allele may emerge through changing maternal behavior impacting how a child is raised (starting from intra-uterine effects to child education), or it may exert its effect directly in the offspring via suppressed gene expression. This weakness can be addressed in the future by inferring untransmitted parental effects and comparing them to that of the transmitted one. We could examine this in the MoBa cohort, where the availability of a large number of parental genomes allowed us to infer parental untransmitted alleles. We observed effects on BMI from maternally transmitted alleles, but no effects from either paternally transmitted alleles or maternally untransmitted alleles, suggesting that maternal rearing effects are unlikely to be the underlying mechanism and aligning more closely with true imprinting effects.

Supplementary Note 16 Power to detect POEs

The detection power of the POE analysis is comparable to that of additive GWAS: The expected test statistic for additive GWAS is $\approx (\beta_M + \beta_P)/2 \cdot \sqrt{N} \cdot \sqrt{2q(1-q)}$. On the other hand, only heterozygous samples are informative to detect POEs, hence its test statistic is $\approx (\beta_M - \beta_P) \cdot \sqrt{N} \cdot \sqrt{q(1-q)/2}$, where β_M and β_P are the per allele maternal and paternal effect size, respectively. In classical POEs scenario, when a SNP has an effect only when it comes from one of the parents (e.g. $\beta_P = 0$), the test statistics become equal. Note that in these situations, an additive effect should also be detectable. The fact that we detect much less POEs is therefore likely driven by the lack of classical patterns (i.e, strict paternal or maternal effect). Thus, besides the genome-wide scan, we explored two additional strategies to boost power: focus on (i) SNPs that show an additive association (since apart from the scenario of $\beta_M = -\beta_P$, the additive effect will not be zero); (ii) imprinted regions (where POEs are more likely). These filters reduce multiple testing burden and hence lead to milder P-value thresholds and can improve statistical power.

Supplementary Note 17 Multi-trait integration reveals broader parental contributions

One of the key advantages of our method is its scalability and applicability to biobank-scale datasets, providing access to a vast array of phenotypes. Such phenotype-rich biobanks allow in-depth POE pheWAS follow-up, which can pinpoint pleiotropic mechanisms underlying multi-trait associations and opens new avenues to follow-up, such as colocalization and Mendelian Randomization to distinguish vertical and horizontal pleiotropy.

For example, we identified POEs associated with standing height and basal metabolic rate at 11p15.5. To better understand the genetic signal at this locus, we explored its associations with several related traits, including weight, waist and hip circumference, and detailed body composition measures such as fat, fat-free, and water mass. Interestingly, while the lead variant showed no significant association with fat percentage across different body regions

✉ Corresponding authors: Robin J. Hofmeister and Zoltán Kutalik

(arm, leg, trunk, or whole body), it was strongly associated with fat-free traits, such as fat-free mass and water mass. This pattern suggests that the genetic signal at 11p15.5 is more likely driven by height and fat-free body mass, which are closely related to lean tissue and skeletal structure, rather than by weight alone.

Another illustrative example comes from the 11p15.5 locus, where we identified POEs on cystatin C, creatinine, and urate levels. Both cystatin C and creatinine are well-established markers of kidney function, and colocalization analyses provided strong evidence for a shared genetic basis for these traits at this locus, likely related to renal filtration capacity. In contrast, urate, a byproduct of purine metabolism that is also filtered by the kidneys, exhibited lower colocalization probabilities with cystatin C and creatinine. This finding suggests that urate regulation, while partly overlapping with GFR-related traits, likely involves additional distinct pathways. Together, these three POEs link this locus to renal functions.

Lastly, we observed POEs on triglycerides (TG), HDL-C, and SHBG at the 7q32.2 region. These traits are established indicators of metabolic health and are closely tied to insulin resistance and metabolic syndrome. Insulin resistance typically raises TG levels while lowering HDL-C and SHBG levels, a pattern that aligns with the observed POEs. Despite the known associations of these traits with T2D risk, no significant associations were detected between the identified variants and T2D, pointing to independent regulatory mechanisms, likely specific to lipid and hormonal pathways.

Supplementary Note 18 Evolutionary insights supporting the parental conflict hypothesis

Analyzing POEs at a biobank scale enables us to move beyond investigating contrasting parental effects on the same trait at a single locus, such as bi-polar effects. While we can explore contrasting effects on the same trait across different loci, a more comprehensive approach leverages the vast range of available phenotypes to examine contrasting parental effects across both traits and loci. This broader perspective allows us to identify overarching patterns and gain deeper insights into the evolutionary dynamics of imprinted genes.

For example, IGF-1 levels exhibited contrasting POEs at two distinct loci. At 6q24.2, we identified a paternal effect decreasing IGF-1 level driven by a variant within the paternally expressed gene *PLAGL1*, which is implicated in transient neonatal diabetes mellitus[18,19]. The role of *PLAGL1* in glucose metabolism and insulin sensitivity[20] underscores the importance of this locus in regulating IGF-1 levels and metabolic pathways. At 16p13.3, we observed a maternal effect increasing IGF-1 levels driven by a variant that acts as eQTL for two maternally expressed genes, *ZNF597* and *NAA60*, as well as for *TRAP1*, a gene linked to mitochondrial function and metformin use[21]. Together, these findings illustrate how contrasting maternal and paternal effects at different loci collectively shape the genetic architecture of IGF-1 levels, offering valuable insights into the broader evolutionary dynamics underlying imprinted loci.

Similarly, for standing height, we identified contrasting parental effect at two independent POE loci at 11p15.5. We observed a paternal effect of rs576603, and a maternal effect of rs143840904, located 900 kb away. This pattern also exemplifies how parental effects can differ at separate loci for the same trait, reflecting a complex interplay of genetic influences.

✉ Corresponding authors: Robin J. Hofmeister and Zoltán Kutalik

Notably, such findings align with the parental conflict theory, which posits that paternal alleles are selected to enhance resource extraction from the mother, promoting offspring growth and increasing the father's genetic success. In this framework, paternally expressed imprinted genes often drive growth and resource acquisition. In contrast, maternally inherited alleles are selected to conserve maternal resources for future reproduction and offspring survival, with maternally expressed genes acting as growth suppressors to counterbalance paternal effects. At the genetic association level, this dynamic is reflected by differential paternal and maternal effects on traits related to resource allocation, often across different loci and in opposing directions, exemplifying bi-polar patterns. This pattern is evident at other loci too, for traits related to resource allocation: at 7q32.2 for SHBG, triglycerides and HDL-cholesterol; at 11p15.5 for T2D, HbA1c, glucose and height, but also for basal metabolic rate, whole body water mass, leg and trunk fat-free masses, and creatinine and cystatine C; at 15q12 for total protein levels, and finally at 20q13.32 for fat percentage in the arms, legs, trunk, and whole body. These findings underscore the role of imprinted genes in balancing maternal and paternal interests across traits linked to resource allocation, fat storage, and energy usage. Their strong presence among our findings (across multiple loci and traits) may support the conflict hypothesis and likely represent footprints of the evolutionary forces shaping the genetic architecture of complex traits.

Supplementary Note 19 Protein QTLs, a new frontier for POEs

We further leveraged protein quantification data both to validate POEs at the protein level and to assess POEs at established protein QTLs (pQTLs). Although the available protein panel did not cover many of the genes located at our complex trait POE-variant, we identified a significant POE-pQTL for *CPA4* when testing variants initially linked to triglycerides, HDL-C, and SHBG. While this could initially suggest a causal relationship between *CPA4*, the lead variants, and these traits, further analysis revealed a much more significant POE-pQTL located approximately 75 kb away from the POE-variant and in moderate linkage disequilibrium ($r^2 > 0.05$). This finding indicates that the detected POEs on triglycerides, HDL-C, and SHBG are unlikely to be driven by *CPA4*, with the moderate significance observed at the POE variant likely arising from linkage disequilibrium with the true causal variant. Instead, other genes at this locus, such as *KLF14* and *MEST*, are more plausible candidates due to their established eQTL associations with the lead variants. In addition, the POE-variant exhibited a paternal effect on *CPA4* protein level, despite the gene being maternally expressed. This likely reflect influence of paternally expressed genes via shared protein network, such as *MEST*[22]. While this finding does not conclusively identify the gene responsible for the observed POEs, it allows us to refine our candidate list, excluding *CPA4* and prioritizing other loci for further study.

Beyond testing complex trait POE-variant for POE-pQTLs, we also evaluated PofO specificity at established pQTLs[17] and identified four significant POE-pQTLs. Two of these were located within known imprinted regions and showed parental effects consistent with the parental expression of the respective genes. For instance, the protein product of the paternally expressed gene *DLK1* was predominantly associated with paternally inherited alleles, though a moderate maternal association was also observed. This biparental influ-

✉ Corresponding authors: Robin J. Hofmeister and Zoltán Kutalik

ence likely reflects the interconnected nature of imprinted gene networks, where maternally expressed genes can indirectly modulate the expression or activity of paternally expressed genes. For example, the imprinting regulation of *DLK1* is also dependent on two differentially methylated regions (DMRs), including the *MEG3* DMR[23], a maternally expressed gene. This highlights the complex epigenetic mechanisms that drive *DLK1* expression and its imprinting pattern. In contrast, the protein product of *CPA4* exhibited a strong maternal association, consistent with its preferential maternal expression[24]. Interestingly, a significant - but moderate - paternal association was also observed for *CPA4*, potentially reflecting network-level interactions with paternally expressed genes such as *MEST*, which may modulate *CPA4* protein levels through shared pathways[22].

The remaining two POE-pQTLs were identified for genes outside known imprinted regions: *ADAM23* and *PER3*, both of which exhibited paternal effects, albeit demonstrating "asymmetric polar" effect. Although *ADAM23* is not currently classified as imprinted, it is located near two paternally expressed genes, *ZDBF2* and *GPR1*, and a recent study reported a paternal bias in its expression[25], suggesting incomplete imprinting. Similarly, *PER3* has been documented to show paternal-biased expression[26], consistent with the paternal effect observed in our study. Additionally, a large-scale DNA methylation study[27] identified three CpG sites near *PER3* that were tested for parent-of-origin-dependent methylation QTLs (POE-mQTLs). Notably, both our lead POE-pQTL, rs228682, and the lead additive pQTL, rs228647, were found to be significant POE-mQTLs for *PER3*. This suggests that the observed POE on PER3 protein levels may be linked to parent-of-origin-dependent DNA methylation mechanisms at this locus.

Interestingly, no significant POE-pQTLs were detected for several other known imprinted genes. This absence may indicate that protein levels do not always reflect RNA expression due to complex feedback mechanisms that could mask detectable PofO associations at the protein level. These results underscore the importance of complementary transcriptomic analyses to capture the full spectrum of POEs. Recent studies focusing on parental-specific eQTLs in parent-offspring trios have successfully validated known imprinted genes and identified novel candidates, emphasizing the value of integrating multi-omics approaches[12].

Supplementary Note 20 Replication analyses support our novel findings

Lastly, we validated several of our findings in the Estonian Biobank (EstBB), a cohort with an enrichment of familial relatedness, though limited in phenotype overlap with our detected POEs. Among the ten associations replicated in the EstBB, six were novel associations, while four corresponded to previously reported loci or variants in high linkage disequilibrium with known associations. Specifically, we confirmed our newly identified bi-polar effect of rs62471721 on triglycerides, rs4731690 on HDL cholesterol, and rs4264135 on creatinine. Additionally, we also validated our novel bi-polar effects of rs4417225 on glucose levels and rs10838787 on HbA1c. While the latter locus had been previously associated with T2D, its link to glucose-related traits provides further, albeit less novel, insights. Notably, among the replicated associations there were bi-polar effects driven by variants acting as eQTLs for distinct genes with contrasting parental expression (for HDL cholesterol and triglycerides).

✉ Corresponding authors: Robin J. Hofmeister and Zoltán Kutalik

This finding further supports the hypothesis that pleiotropic effects may offer a mechanistic explanation for bi-polar dominant phenomena. Not all POEs identified in the UKBB were replicated in the EstBB, which could reflect differences in statistical power, cohort characteristics, or environmental factors, or false positive associations. For instance, while two of the POEs on standing height at 11p15.5 were successfully replicated, the third association at the same locus was not. This discrepancy could arise from cohort-specific imprinting or parental nurturing effects.

Interestingly, a POE not replicated in the Estonian cohort — the maternal effect on hip circumference — was validated in the MoBa cohort, where it showed a significant effect on infant BMI. This effect, which operates in opposite directions during infancy and adulthood, highlights the importance of examining POEs at different stages of life to capture the dynamic nature of genetic imprinting.

Supplementary Note 21 Validation of Linkage Disequilibrium (LD) equivalence in Parent-of-Origin Effects and its applications to heritability and colocalization analyses

A key consideration when analyzing POEs is whether traditional LD structures, commonly used in additive genetic studies, remain valid when applied to differential genotypes (i.e., $G_M - G_P$). Given that recombination rates differ between male and female meioses, one might expect that LD could vary between maternal and paternal haplotypes. However, because each individual's haplotype is a recombined mixture of multiple past generations — each subject to both male and female recombinations — LD differences between maternal and paternal haplotypes can only be due to a single generation of sex-specific recombinations.

21.1 Empirical observations: LD Consistency Across Parental Haplotypes

To validate this theory, we empirically compared LD computed separately from maternally inherited haplotypes and paternally inherited haplotypes using the UK Biobank dataset. Specifically, we:

- Computed LD matrices using maternally inherited haplotypes.
- Computed LD matrices using paternally inherited haplotypes.
- Correlated these LD estimates at the 11p15.5 imprinted region (Supplementary Figure 23A).

We observed a strong correlation (Pearson R = 0.999963) between $LD_{paternal}$ and $LD_{maternal}$, with only minor deviations (mean absolute error = 3.55×10^{-4}). These deviations were comparable to those observed when traditional LD (i.e from genotype data) is computed in two non-overlapping groups of 100,000 individuals (Pearson R = 0.999961, mean absolute error=3.47×10^{-4}, Supplementary Figure 23B), indicating that LD structure does not systematically differ between maternal and paternal haplotypes.

✉ Corresponding authors: Robin J. Hofmeister and Zoltán Kutalik

21.2 Theoretical derivations

To formally test whether LD structures remain consistent between parental haplotypes, we derived the theoretical relationship between LD for differential genotypes and conventional LD for additive genotypes.

Let $G_M, G_P \in \mathcal{R}^n$ denote the maternal and paternal genotypes of SNP G. Similarly, F_M, F_P denote the respective genotypes for SNP F in the genome. We write their sum as $G := G_M + G_P$ and $F := F_M + F_P$. In the following we will compute the correlation between the differential genotypes $G_M - G_P$ and $F_M - F_P$. To do so, we will make the following plausible assumptions:

- male and female allele frequencies do not differ (this has been shown via large GWAS studies on sex, which revealed extremely minor differences only due to sex-differential study selection bias[28]). It follows that $E[G_M] = E[G_P] = q_G, E[F_M] = E[F_P] = q_F$.
- LD is the same in men and women (even if recombination positions differ between men and women, given that an individual's maternal chromosome is a recombined version of a male and a female genome (one generation before) it has minor impact on LD (see the empirical evidence above). It follows that $r(G_M, F_M) = r(G_P, F_P) = r(G, F)$.

$$\begin{aligned} cov((G_M - G_P),(F_M - F_P)) &= E[(G_M - G_P)\cdot(F_M - F_P)] && (3)\\ &= E[(G_M + G_P)\cdot(F_M + F_P)] - 2\cdot E[G_M\cdot F_P + G_P\cdot F_M] && (4)\\ &= E[G\cdot F] - 2\cdot E[G_M\cdot F_P] - 2\cdot E[G_P\cdot F_M] && (5)\end{aligned}$$

The correlation between a maternal genotype at SNP G and a paternal genotype at SNP F can only happen through assortative mating for a trait Y. It we define $\rho := r(Y_P, Y_M)$, $\beta_G := r(Y_M, G_M) = r(Y_P, G_P)$, $\beta_F := r(Y_M, F_M) = r(Y_P, F_P)$ we can then express:

$$r(G_M, F_P) = r(G_P, F_M) \approx \rho\cdot\beta_G\cdot\beta_F < 10^{-4} \quad (6)$$

The last inequality is assuming that individual SNPs explain less than 0.1% of trait variance on average and phenotypic correlation between couples is less than 10% on average - both of which are widely supported by real data. Hence,

$$\begin{aligned} E[G_M\cdot F_P] &= E[G_M]\cdot E[F_P] + r(G_M, F_P)\cdot\sqrt{Var(G_M)\cdot Var(F_P)} && (7)\\ &= q_G\cdot q_F + e \approx q_G\cdot q_F && (8)\end{aligned}$$

because $|e| < 10^{-4}\cdot\sqrt{q_G(1-q_G)\cdot q_F(1-q_F)}$. Therefore,

✉ Corresponding authors: Robin J. Hofmeister and Zoltán Kutalik

$$(G_M - G_P) \cdot (F_M - F_P) \approx E[G \cdot F] - E[G] \cdot E[F] = cov(G, F) \tag{9}$$

Similarly, for the variance of the difference genotypes:

$$\begin{aligned}
Var(G_M - G_P) &= E[G_M^2] + E[G_P^2] - 2E[G_M \cdot G_P] - E^2[G_M - G_P] && (10)\\
&= E[G_M^2] + E[G_P^2] - 2E[G_M \cdot G_P] && (11)\\
&= E[(G_M + G_P)^2] - 4E[G_M \cdot G_P] && (12)\\
&\approx E[G^2] - 4E[G_M] \cdot E[G_P] && (13)\\
&\approx E[G^2] - 4q_G^2 && (14)\\
&\approx Var(G) && (15)\\
& && (16)
\end{aligned}$$

The approximation comes from the fact that we ignored the negligible difference induced by assortative mating (similar computation as above for $r(G_M, F_P)$). Similar derivation can be done to obtain $Var(F_M - F_P) = Var(F)$. Finally, this allows us to demonstrate that:

$$\begin{aligned}
r((G_M - G_P), (F_M - F_P)) &= \frac{cov((G_M - G_P), (F_M - F_P))}{\sqrt{Var(G_M - G_P) \cdot Var(F_M - F_P)}} && (17)\\
&= \frac{cov(G, F)}{\sqrt{Var(G) \cdot Var(F)}} && (18)\\
&= r(G, F) && (19)
\end{aligned}$$

Therefore, the LD between the maternal-paternal difference genotypes of two SNPs is the same as the LD between these SNPs in additive coding.

Note that the uni-variable linear regression estimates from $Y \sim G_M - G_P$ are identical if we use the full data *vs* if we use only the samples that are heterozygous for G (i.e. $G_M - G_P = 0$). The reason for this is that $E[Y \cdot (G_M - G_P)]$ does not change if we remove individuals for which $G_M - G_P = 0$ and same golds for $E[(G_M - G_P)^2]$.

To obtain heritability estimates for the parental differential genotypes we can use association summary statistics from the $Y \sim G_M - G_P$ model (restricted or not to non-zero values of $G_M - G_P$) and regular LD scores can be used (that are already in place for additive genotype GWAS).

21.3 Application to heritability estimation

Given the theoretical derivation and empirical validation, we conclude that conventional LD reference panels can be used for heritability estimation of POEs. Specifically, for heritability estimation using LD Score Regression (LDSC), we leverage association summary statistics from the differential POE model:

$$Y \sim G_M - G_P \tag{20}$$

✉ Corresponding authors: Robin J. Hofmeister and Zoltán Kutalik

Importantly, we show that this regression model yields identical estimates regardless of whether we use the full dataset or restrict to heterozygous individuals only. This ensures that LDSC-derived SNP-heritability (h^2_{POE}) can be computed using standard LD scores without requiring recalibration for POE analyses.

21.4 Application to colocalization analyses

The equivalence of LD structures between differential genotypes and tota genotypes also supports the use of standard colocalization approaches. Specifically, (i) given that colocalization methods rely on LD to model shared causal effects across traits, the use of conventional LD structures remains valid for POE studies; (ii) because LD differences between parental haplotypes are negligible, maternal and paternal POE effects can be analyzed using standard colocalization frameworks without introducing biases.

✉ Corresponding authors: Robin J. Hofmeister and Zoltán Kutalik

Supplementary Figures

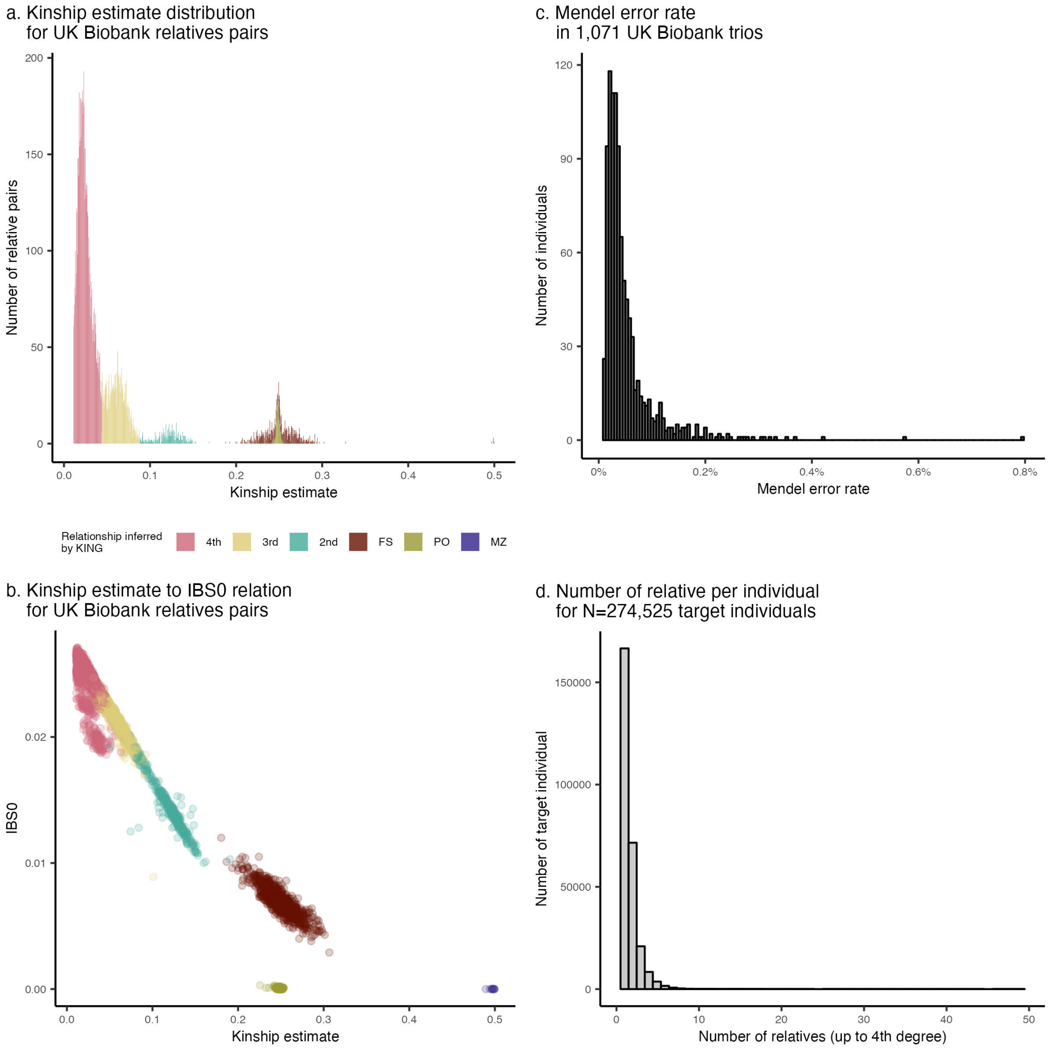

Supplementary Fig. 1 | Relatedness inference and close relative clustering in the UK Biobank. **a**) Kinship estimates distribution and **b**) Kinship estimates versus IBS0 estimates across all UK Biobank monozygotic twins (MZ), first siblings (FS), parent-offspring (PO), second-, third-, and fourth-degree relative pairs. **c**) Mendel error rate distribution across 1,071 UK Biobank parent-offspring trios. None of the trios exhibited a high error rate that would necessitate exclusion. **d**) Distribution of the number of close relatives (up to the fourth degree) per individual across 274,252 UK Biobank individuals with available surrogate parent cluster(s). We found on average 1.65 relatives per individual.

✉ Corresponding authors: Robin J. Hofmeister and Zoltán Kutalik

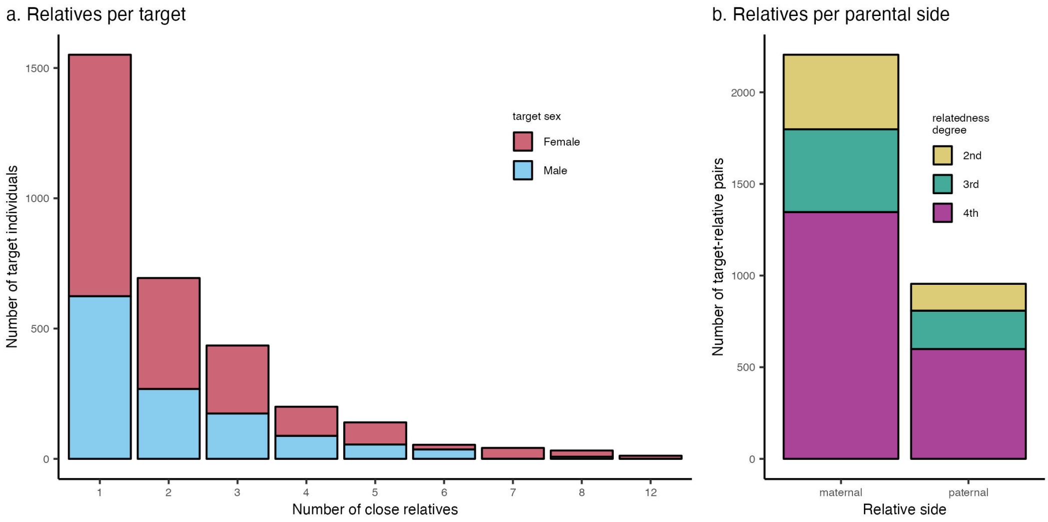

Supplementary Fig. 2 | Validation cohort structure. a) Distribution of the number of close relatives (up to the fourth degree) per individual across 2,141 UK Biobank individuals with both available surrogate parent cluster(s) and available parental genomes. Most target individuals are females (60.3%) and have a single surrogate parent (49%). **b**) Distribution of surrogate mother and surrogate father stratified by relatedness degree for 3,160 target-relative pairs. Approximately 70% of surrogate parents in the validation cohort are on the maternal side (i.e., surrogate mothers), and the majority are fourth-degree relatives pairs (61.5%).

✉ Corresponding authors: Robin J. Hofmeister and Zoltán Kutalik

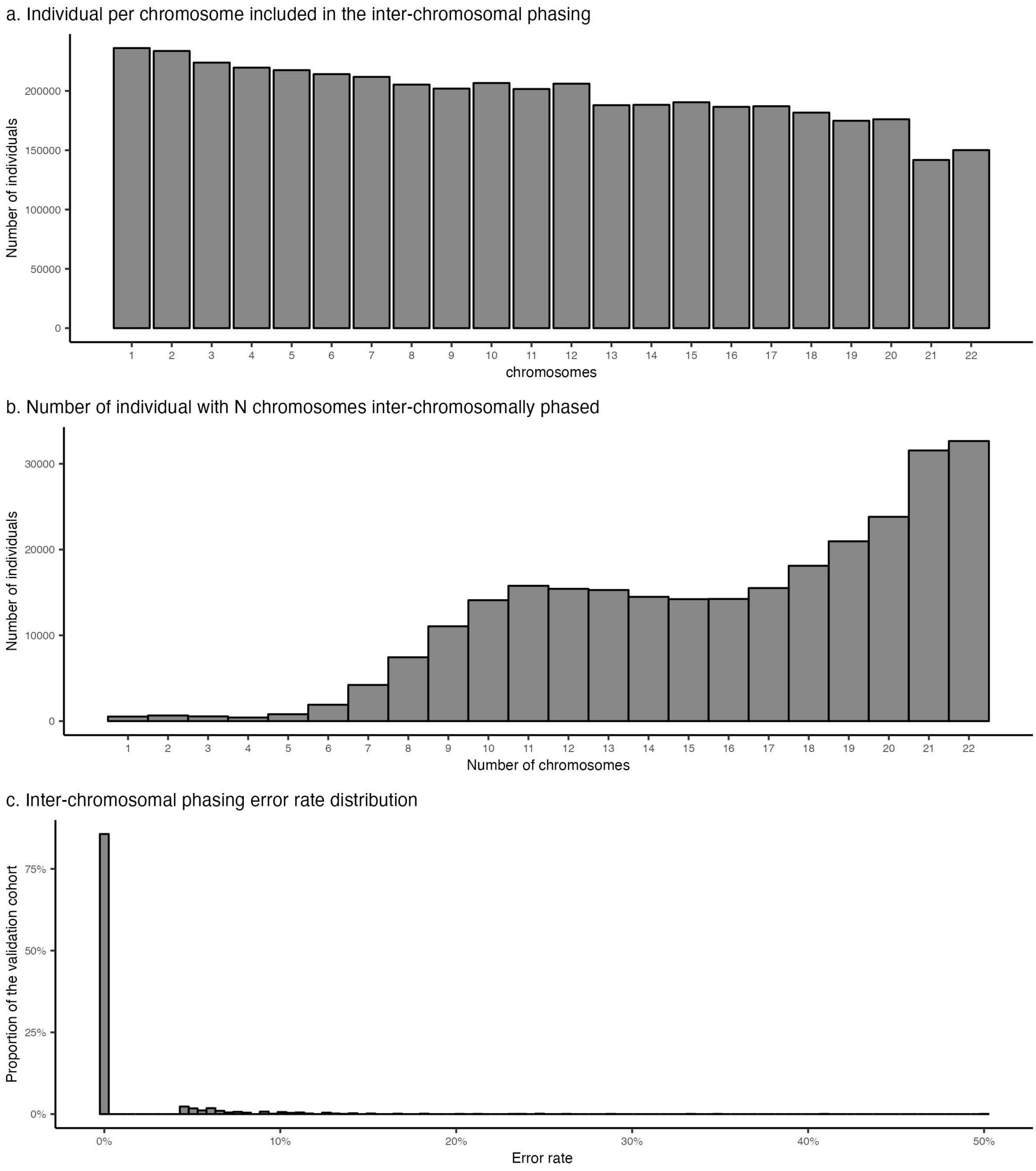

Supplementary Fig. 3 | Inter-chromosomal phasing from close relative groups. **a**) Distribution of the number of individuals with a given chromosome included in the inter-chromosomal phasing. The sample sizes vary across chromosomes, as inter-chromosomal phasing requires at least one IBD segment shared with a surrogate parent on the chromosome. Larger chromosomes are more frequently included, likely due to a higher likelihood of containing at least one IBD segment. **b**) Distribution of the number of individuals with N chromosomes (x-axis) successfully included in the inter-chromosomal phasing. Only 12% of individuals (32,651) had all 22 autosomes phased inter-chromosomally. On average, individuals had 16 chromosomes included in inter-chromosomal phasing. **c**) Distribution of inter-chromosomal phasing error rates across individuals in the validation cohort. Error rates were calculated by comparing phasing results based on surrogate parents to the ground truth obtained from available parental genomes. Most individuals (85.6%) were perfectly phased, with an average error rate of only 1.06% for flipped haplotypes, underscoring the robustness and reliability of the inter-chromosomal phasing method in accurately resolving parental haplotype sets.

✉ Corresponding authors: Robin J. Hofmeister and Zoltán Kutalik

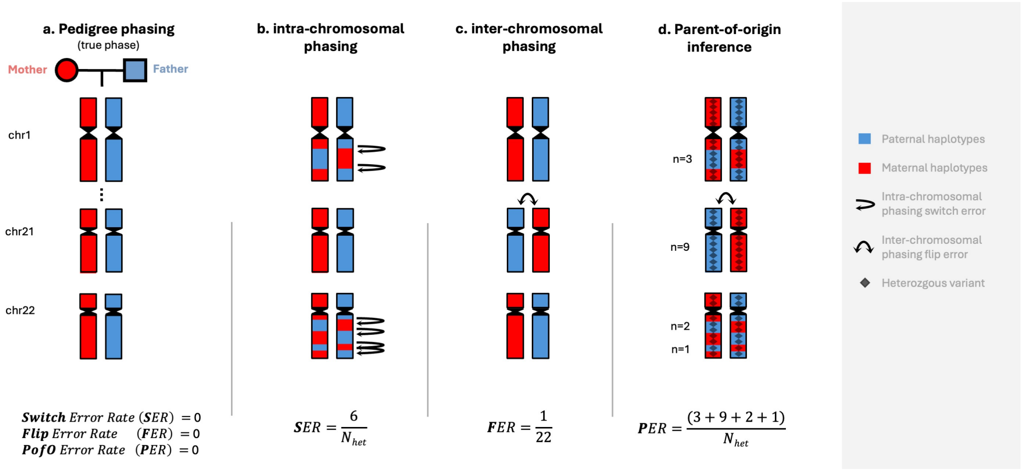

Supplementary Fig. 4 | Illustration of errors in statistical methods. a) A perfectly phased individual (both intra- and inter-chromosomally). Phasing is performed using the parental genomes: maternal (red) and paternal (blue). In this scenario, the PofO is directly inferred alongside the phasing process without errors. **b**) An individual with intra-chromosomal phasing errors introduced by traditional phasing software (e.g., SHAPEIT5[1]). These errors are represented as switches between parental haplotypes within the same chromosome (horizontal arrows), with 6 switches shown here. Since switches occur only at heterozygous sites, the switch error rate (SER) is calculated as $SER = 6/N_{het}$, where N_{het} is the total number of heterozygous sites. **c**) An individual with inter-chromosomal phasing errors but assumed perfect intra-chromosomal phasing (no switches within the same chromosome). Errors in this case are represented as flips of entire parental haplotypes across chromosomes (vertical arrow), with one flip shown here. The flip error rate (FER) is calculated as the proportion of chromosomes flipped relative to the majority, ranging from 0% to 50%. **d**) An individual with combined intra- and inter-chromosomal phasing errors. PofO inference errors aggregate contributions from both switch and flip errors. The PofO error rate (PER) is computed as the proportion of heterozygous sites (grey diamonds) incorrectly assigned. For example, in this scenario, assigning the first haplotype as maternal and the second as paternal results in a total of 15 errors.

✉ Corresponding authors: Robin J. Hofmeister and Zoltán Kutalik

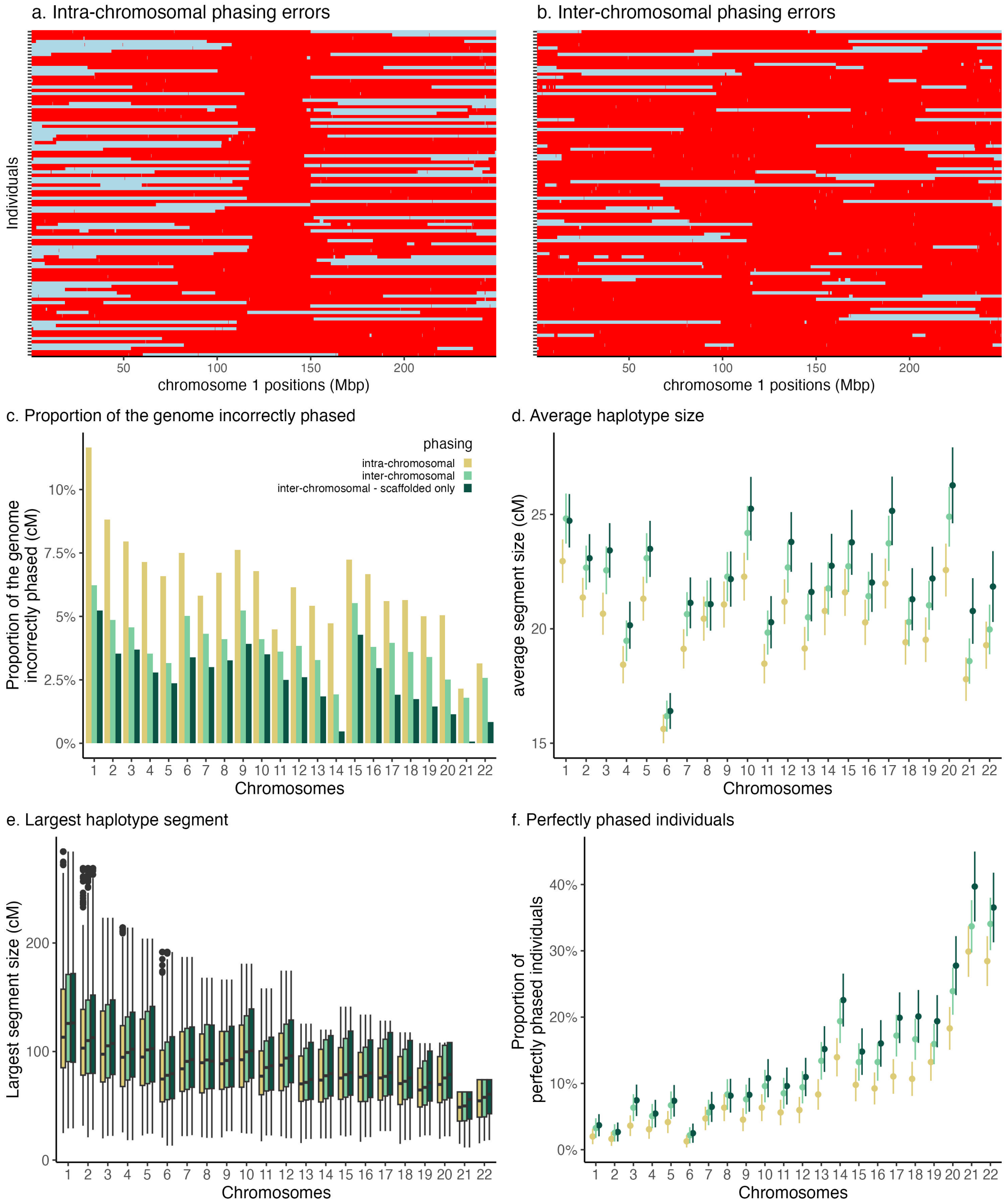

Supplementary Fig. 5 | Phasing error rates. Intra-chromosomal (**a**) and inter-chromosomal (**b**) phasing errors for 100 randomly selected trios-offspring. Each line (y-axis) represent an individual along chromosome 1 (x-axis). Switch between red and blue represents a phasing error between parental haplotypes. **c**) Proportion of the genome in centimorgan (cM) incorrectly phased (y-axis) for each chromosome (x-axis) when phased using intra-chromosomal phasing (yellow) or inter-chromosomal phasing (light green), computed genome-wide. Dark green show the proportion of error when restricting to chromosomes where IBD-sharing relatives were available, such as used for PofO inference. **d**) Average size of phased haplotype segments (y-axis) per chromosome (x-axis). Dots represent average size, while error bars indicate 95% confidence intervals. **e**) Distribution of the largest haplotype segment per individual (y-axis) per chromosome (x-axis). Boxes indicate the interquartile range (IQR), with the bottom and top of the box representing the 25th (Q1) and 75th (Q3) percentiles, respectively. The horizontal line within the box represents the median (50th percentile). Whiskers extend to the smallest and largest values within $Q1-1.5 \times IQR$ and $Q3 + 1.5 \times IQR$. **e**) Proportion of individuals perfectly phased (y-axis) per chromosome (x-axis). Dots represent the proportion, while error bars indicate 95% confidence intervals.

✉ Corresponding authors: Robin J. Hofmeister and Zoltán Kutalik

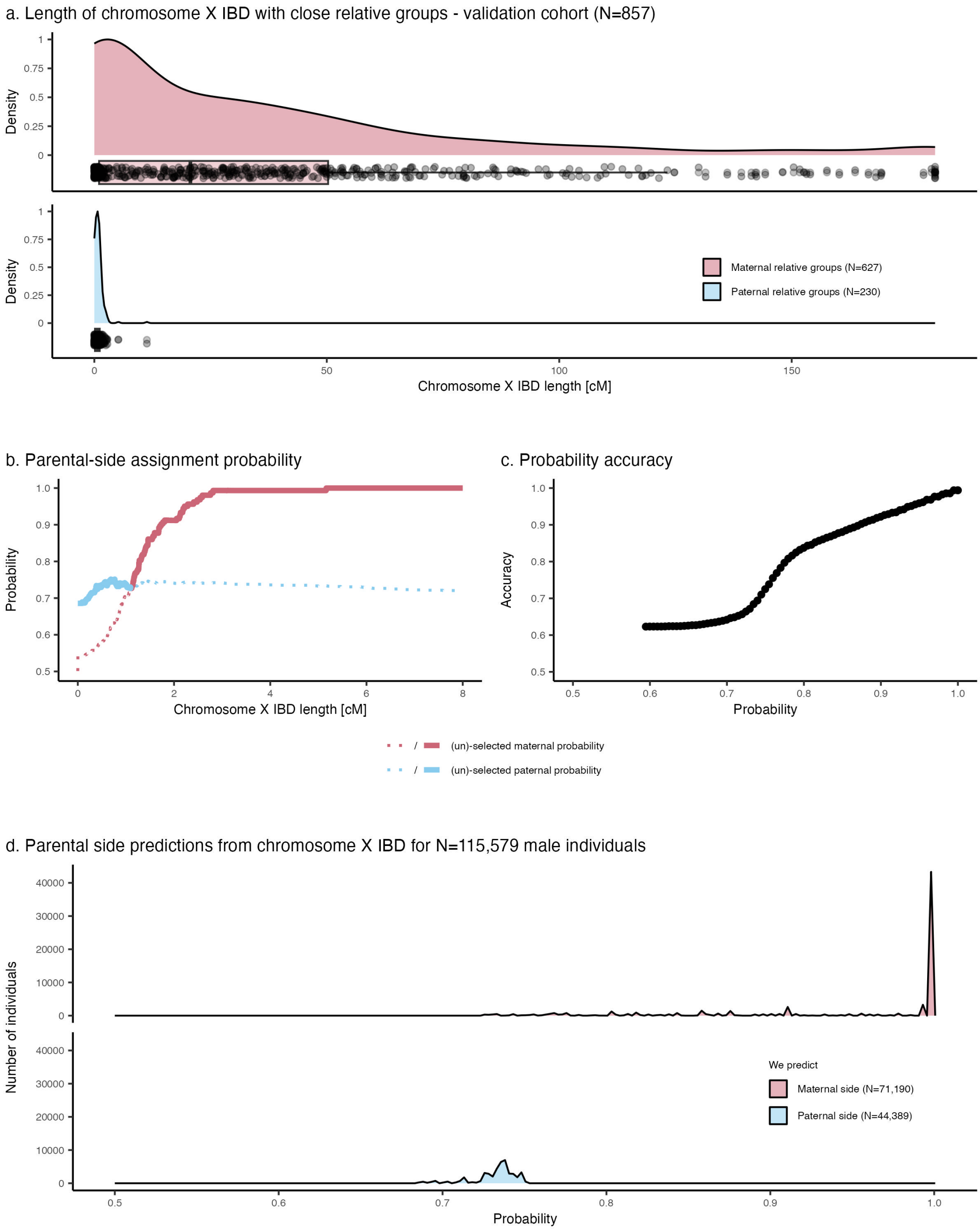

Supplementary Fig. 6 | Parental side assignment from chromosome X IBD analysis in male individuals. a) Density distribution of chromosome X IBD segment length in centimorgan (cM) between targets and surrogate father (blue) and targets and surrogate mother (red) across the validation cohort's male individuals. Boxes indicate the interquartile range (IQR), with the bottom and top of the box representing the 25th (Q1) and 75th (Q3) percentiles, respectively. The horizontal line within the box represents the median (50th percentile). Whiskers extend to the smallest and largest values within $Q1-1.5\times IQR$ and $Q3+1.5\times IQR$. Each point represent a target-relative pair. **b)** Parental side probabilities depending on chromosome X IBD segment length derived from the validation cohort. Selected and unselected maternal (red) and paternal (blue) probabilities for a given x-axis value are indicated by solid and dotted lines, respectively. **c)** Accuracy of parental side predictions (y-axis) as a function of the parental side probability (x-axis). **d)** Distribution of maternal (red) and paternal (blue) side assignment probabilities derived chromosome X IBD across 115,579 UK Biobank male individuals.

✉ Corresponding authors: Robin J. Hofmeister and Zoltán Kutalik

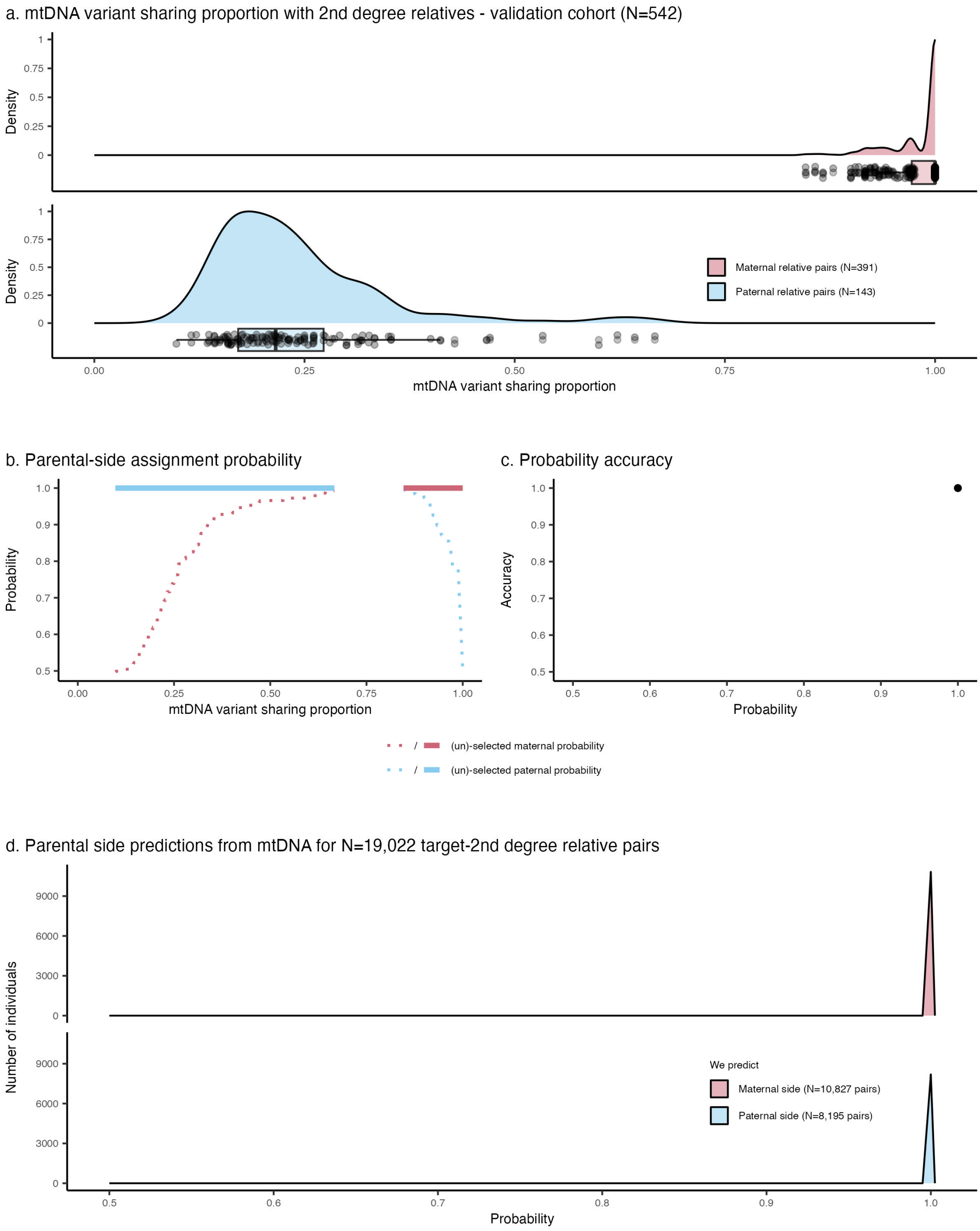

Supplementary Fig. 7 | Parental side assignment from mitochondrial DNA Minor Variant Sharing (MVS) analysis in 2nd degree relative pairs. **a**) Density distribution of mtDNA Minor Variant Sharing (MVS) between targets and 2nd degree surrogate father (blue) and targets and 2nd degree surrogate mother (red) across the validation cohort's individuals. Boxes indicate the interquartile range (IQR), with the bottom and top of the box representing the 25th (Q1) and 75th (Q3) percentiles, respectively. The horizontal line within the box represents the median (50th percentile). Whiskers extend to the smallest and largest values within $Q1-1.5\times IQR$ and $Q3+1.5\times IQR$. Each point represent a target-relative pair. **b**) Parental side probabilities depending on mtDNA MVS derived from the validation cohort. Selected and unselected maternal (red) and paternal (blue) probabilities for a given x-axis value are indicated by solid and dotted lines, respectively. **c**) Accuracy of parental side predictions (y-axis) as a function of the parental side probability (x-axis). **d**) Distribution of maternal (red) and paternal (blue) side assignment probabilities derived from mtDNA MVS across 19,022 UK Biobank 2nd degree relative pairs.

✉ Corresponding authors: Robin J. Hofmeister and Zoltán Kutalik

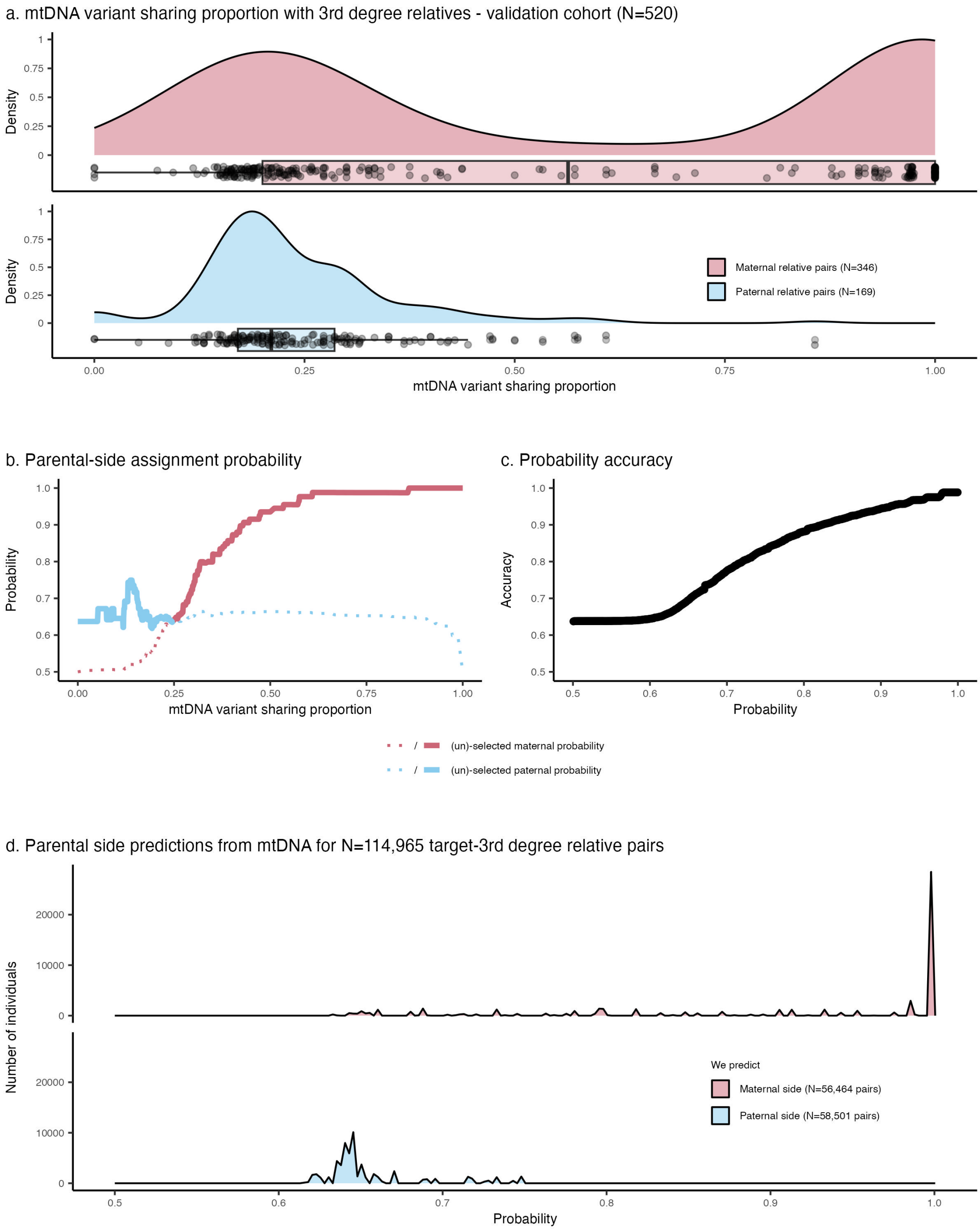

Supplementary Fig. 8 | Parental side assignment from mitochondrial DNA Minor Variant Sharing (MVS) analysis in 3rd degree relative pairs. **a**) Density distribution of mtDNA Minor Variant Sharing (MVS) between targets and 3rd degree surrogate father (blue) and targets and 3rd degree surrogate mother (red) across the validation cohort's individuals. Boxes indicate the interquartile range (IQR), with the bottom and top of the box representing the 25th (Q1) and 75th (Q3) percentiles, respectively. The horizontal line within the box represents the median (50th percentile). Whiskers extend to the smallest and largest values within $Q1-1.5 \times IQR$ and $Q3 + 1.5 \times IQR$. Each point represent a target-relative pair. **b**) Parental side probabilities depending on mtDNA MVS derived from the validation cohort. Selected and unselected maternal (red) and paternal (blue) probabilities for a given x-axis value are indicated by solid and dotted lines, respectively. **c**) Accuracy of parental side predictions (y-axis) as a function of the parental side probability (x-axis). **d**) Distribution of maternal (red) and paternal (blue) side assignment probabilities derived from mtDNA MVS across 114,965 UK Biobank 3rd degree relative pairs.

✉ Corresponding authors: Robin J. Hofmeister and Zoltán Kutalik

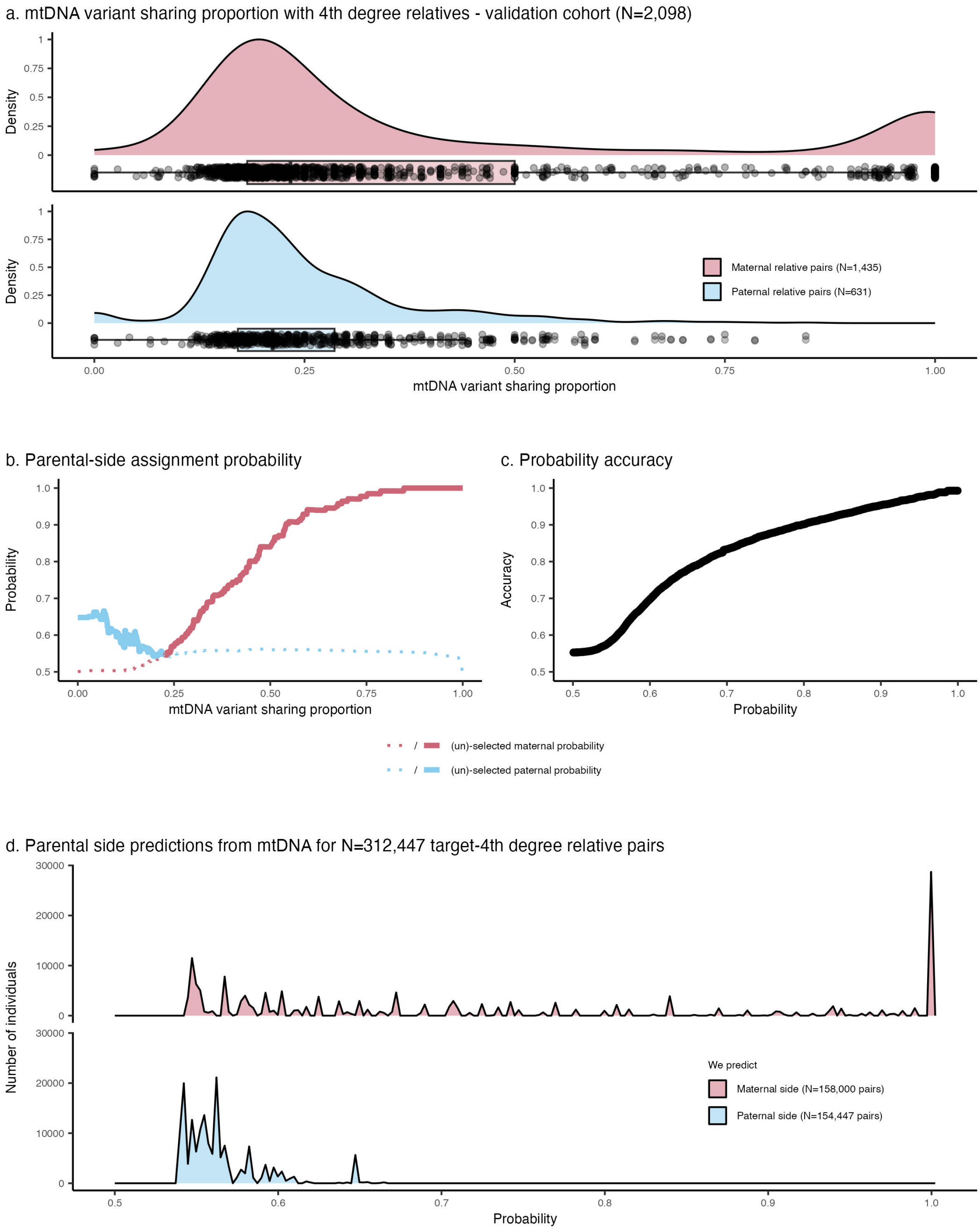

Supplementary Fig. 9 | Parental side assignment from mitochondrial DNA Minor Variant Sharing (MVS) analysis in 4th degree relative pairs. **a**) Density distribution of mtDNA Minor Variant Sharing (MVS) between targets and 4th degree surrogate father (blue) and targets and 4th degree surrogate mother (red) across the validation cohort's individuals. Boxes indicate the interquartile range (IQR), with the bottom and top of the box representing the 25th (Q1) and 75th (Q3) percentiles, respectively. The horizontal line within the box represents the median (50th percentile). Whiskers extend to the smallest and largest values within $Q1-1.5 \times IQR$ and $Q3 + 1.5 \times IQR$. Each point represent a target-relative pair. **b**) Parental side probabilities depending on mtDNA MVS derived from the validation cohort. Selected and unselected maternal (red) and paternal (blue) probabilities for a given x-axis value are indicated by solid and dotted lines, respectively. **c**) Accuracy of parental side predictions (y-axis) as a function of the parental side probability (x-axis). **d**) Distribution of maternal (red) and paternal (blue) side assignment probabilities derived from mtDNA MVS across 312,447 UK Biobank 4th degree relative pairs.

✉ Corresponding authors: Robin J. Hofmeister and Zoltán Kutalik

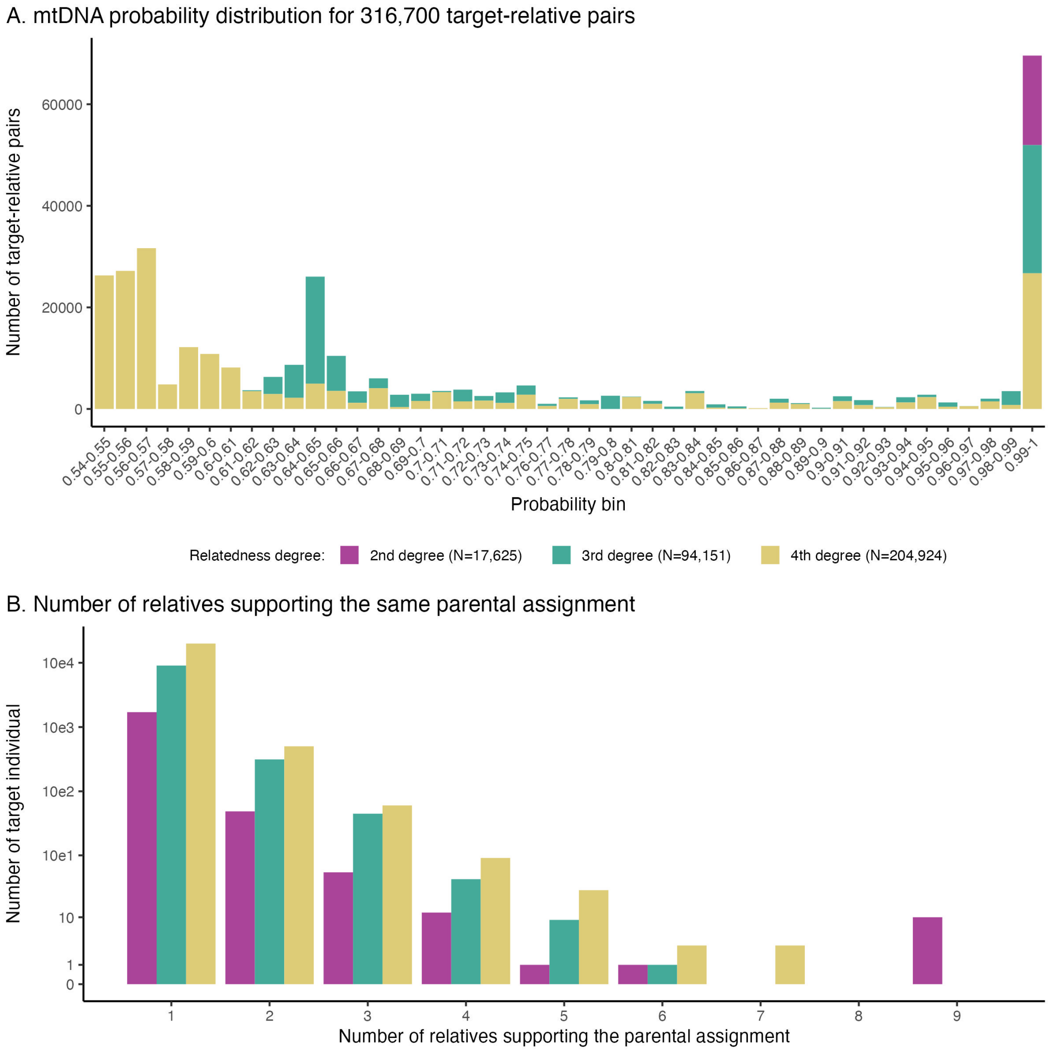

Supplementary Fig. 10 | Selection of mitochondrial DNA (mtDNA) Minor Variant Sharing (MVS) predictors for parental side assignments. **a**) Distribution of parental side assignment probabilities for 316,700 target-relative pairs, stratified by relatedness degree (2nd, 3rd, and 4th degree). For individuals with multiple relatives of the same degree, only the relative with the highest predicted accuracy was retained. Probabilities are binned at intervals of 0.01 for visualization. **b**) Log-scale distribution of the number of target individuals (y-axis) based on the number of relatives (x-axis) supporting the same parental side assignment, further stratified by degree of relatedness.

✉ Corresponding authors: Robin J. Hofmeister and Zoltán Kutalik

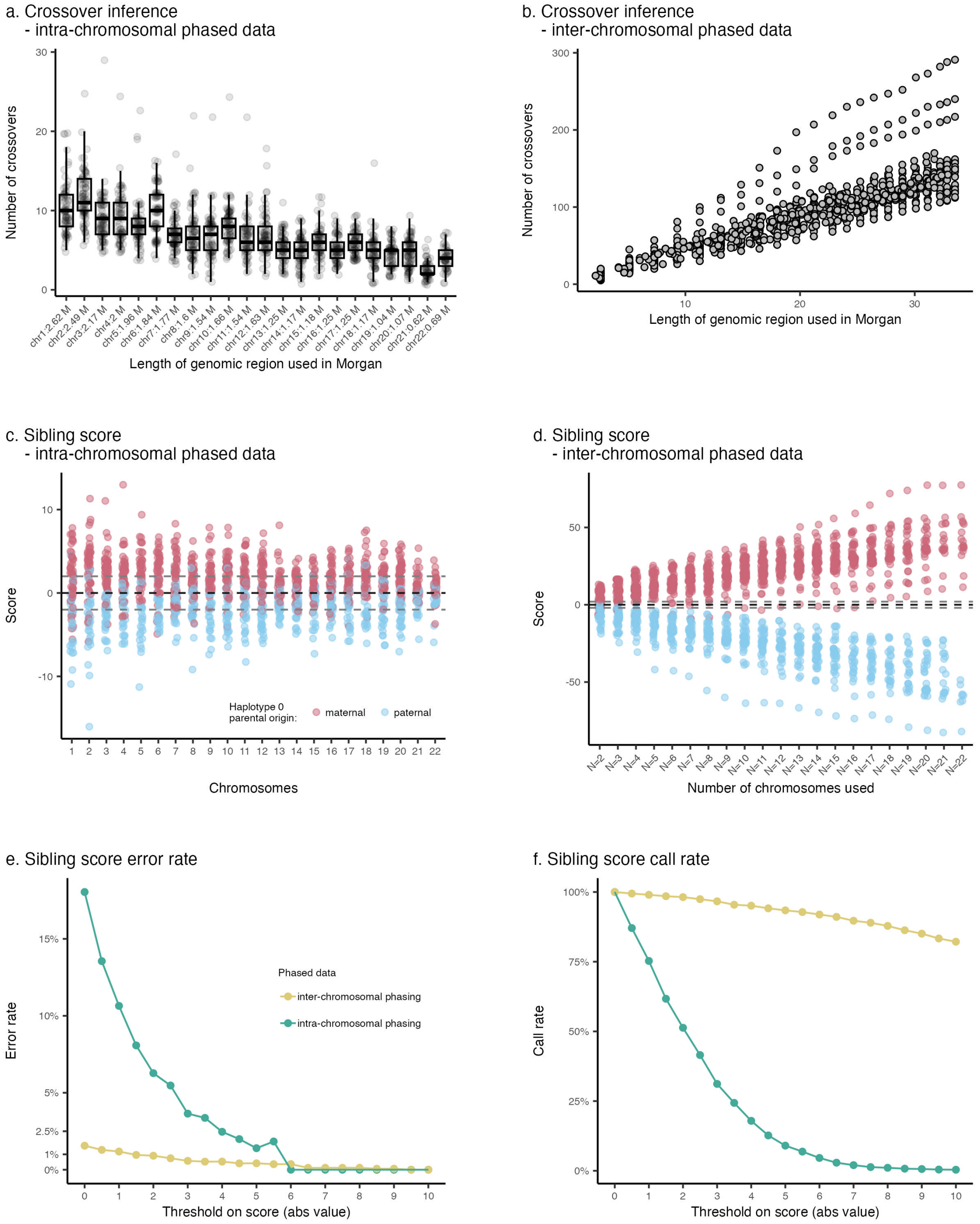

Supplementary Fig. 11 | Evaluation of sibling scores from intra- and inter-chromosomal phasing in N=88 individuals of the validation cohort. **a**) Distribution of the number of inferred crossovers (y-axis) per chromosome (x-axis) derived from intra-chromosomally phased data. **b**) Distribution of the number of inferred crossovers (y-axis) relative to the genomic length used (in Morgans, x-axis) derived from inter-chromosomally phased data. **c**) Distribution of sibling scores (y-axis) per chromosome (x-axis) using intra-chromosomally phased data. Maternal and paternal haplotypes are color-coded in red and blue, respectively. **d**) Distribution of sibling scores (y-axis) relative to the number of chromosomes included in inter-chromosomally phased data (x-axis). **e**) Error rates (y-axis) of sibling scores for varying score thresholds (absolute values, x-axis). For example, excluding ambiguous scores between -2 and 2 reduced error rates to 1.4% with inter-chromosomal phasing, compared to 6.4% with intra-chromosomal phasing. **f**) Call rates (y-axis) of sibling scores for varying score thresholds (absolute values, x-axis), showing the proportion of individuals meeting the threshold criteria.

✉ Corresponding authors: Robin J. Hofmeister and Zoltán Kutalik

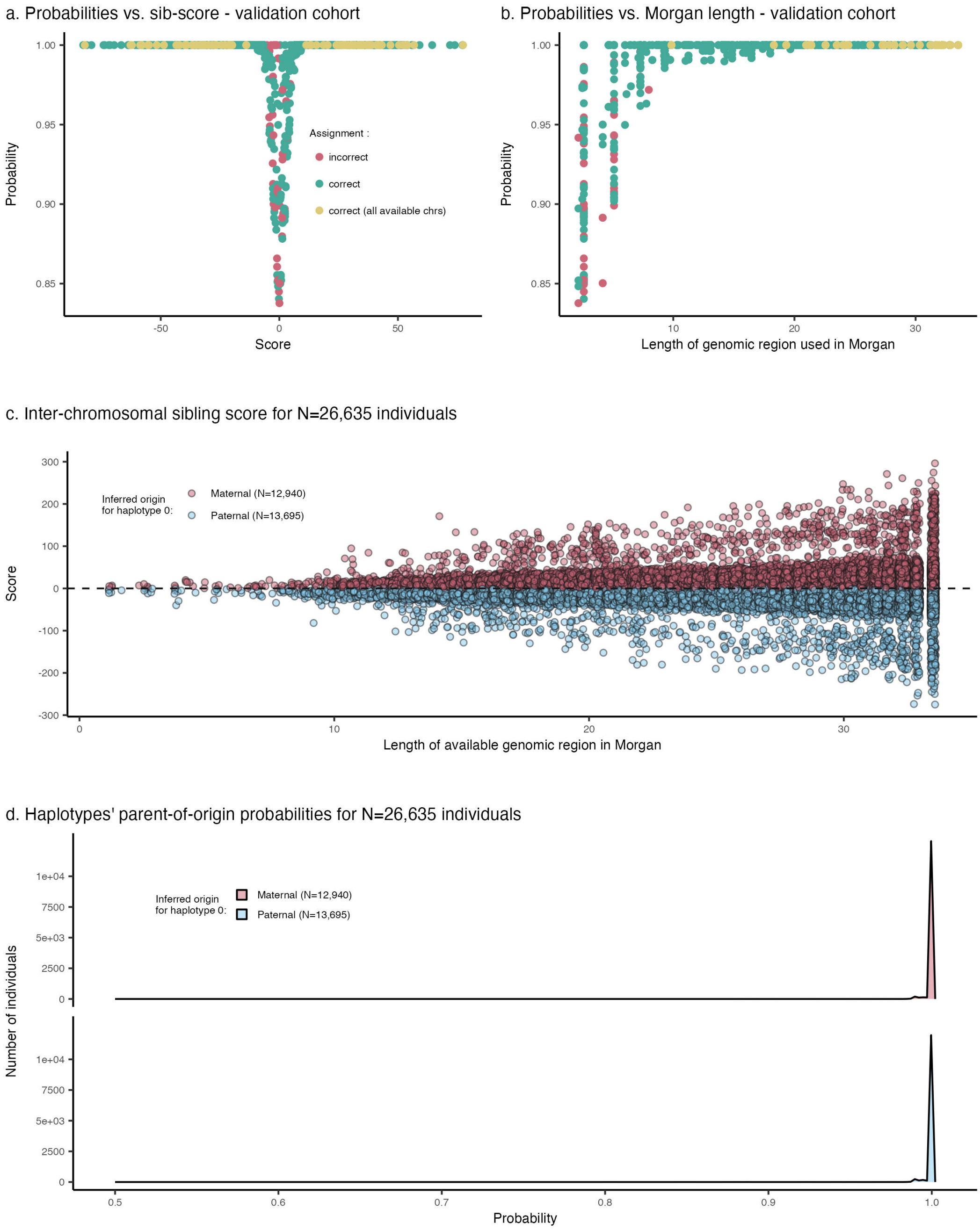

Supplementary Fig. 12 | Validation and derivation of parent-of-origin probabilities using sibling scores from inter-chromosomal phased data. a) PofO probabilities (y-axis) as a function of sibling scores (x-axis) in the validation cohort. Each dot represents a specific configuration (an individual with a given number of chromosomes used). We varied the number of chromosome used per individual to assess the accuracy of different configurations (see Supplementary Figure 11D). Red dots indicate incorrect parental assignments, green dots indicate correct assignments, and yellow dots denote correct assignments using the maximum available chromosomes (N_{max}, corresponds to the full set of inter-chromosomally phased chromosome for a given individual). No errors were observed when N_{max} chromosomes were used. **b**) PofO probabilities (y-axis) plotted against the total available genomic length in Morgans (x-axis) for sibling score calculation in the validation cohort. Each configuration is represented as a dot (color-coded as in panel a). Increasing genomic length results in higher assignment accuracy, with perfect accuracy achieved at N_{max} (yellow dots). **c**) Sibling scores (y-axis) derived for N=26,635 individuals across available genomic lengths (x-axis). Most configurations (99.5%) cover more than 10 Morgans and include over 30 inferred crossover events, ensuring high assignment accuracy (as indicated in the validation panels a and b). **d**) Distribution of PofO probabilities for 26,635 individuals. A majority of individuals (>95%) exhibit probabilities of 1, resulting in perfect accuracy within the validation cohort (see yellow dots in panel a).

✉ Corresponding authors: Robin J. Hofmeister and Zoltán Kutalik

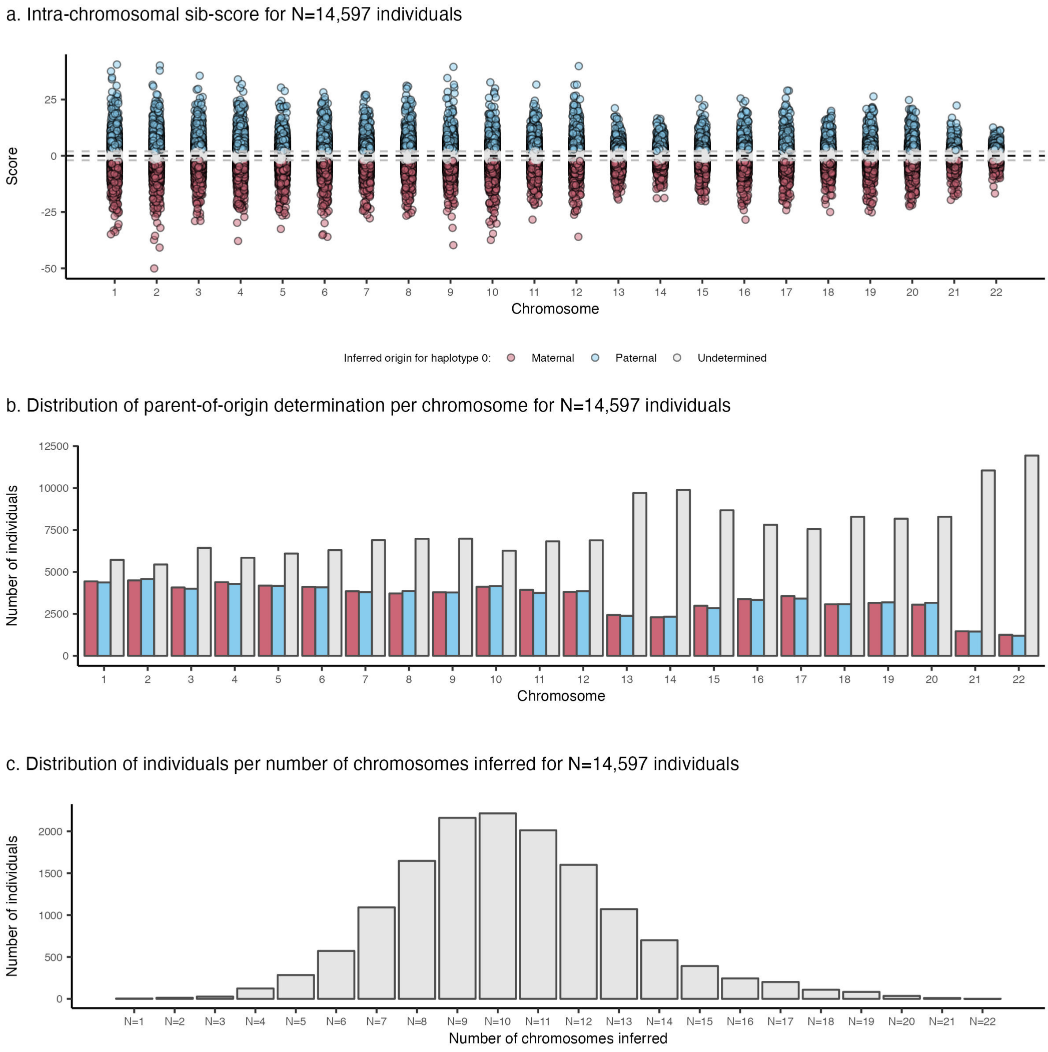

Supplementary Fig. 13 | Derivation of parent-of-origin probabilities using sibling scores from intra-chromosomal phased data. a) Sibling scores calculated for each chromosome independently (x-axis). Each dot corresponds to a single chromosome, with blue and red dots indicating paternal and maternal assignment, respectively, for the first haplotype. Undetermined assignments are shown in gray. **b**) Distribution of the number of individuals (y-axis) with inferred PofO for each chromosome (x-axis). Larger chromosomes exhibit higher numbers of PofO inference (e.g., 8,811 individuals for chromosome 1), while smaller chromosomes show fewer inferences (e.g., 2,450 individuals for chromosome 22), reflecting the increased likelihood of crossovers on larger chromosomes. **c**) Distribution of the number of chromosomes with inferred PofO per individual (x-axis), with counts shown on the y-axis. On average, individuals have 10.3 chromosomes with PofO inferred. This is lower compared to the average of 16 chromosomes inferred using inter-chromosomal phasing (see Supplementary Figure 3B).

✉ Corresponding authors: Robin J. Hofmeister and Zoltán Kutalik

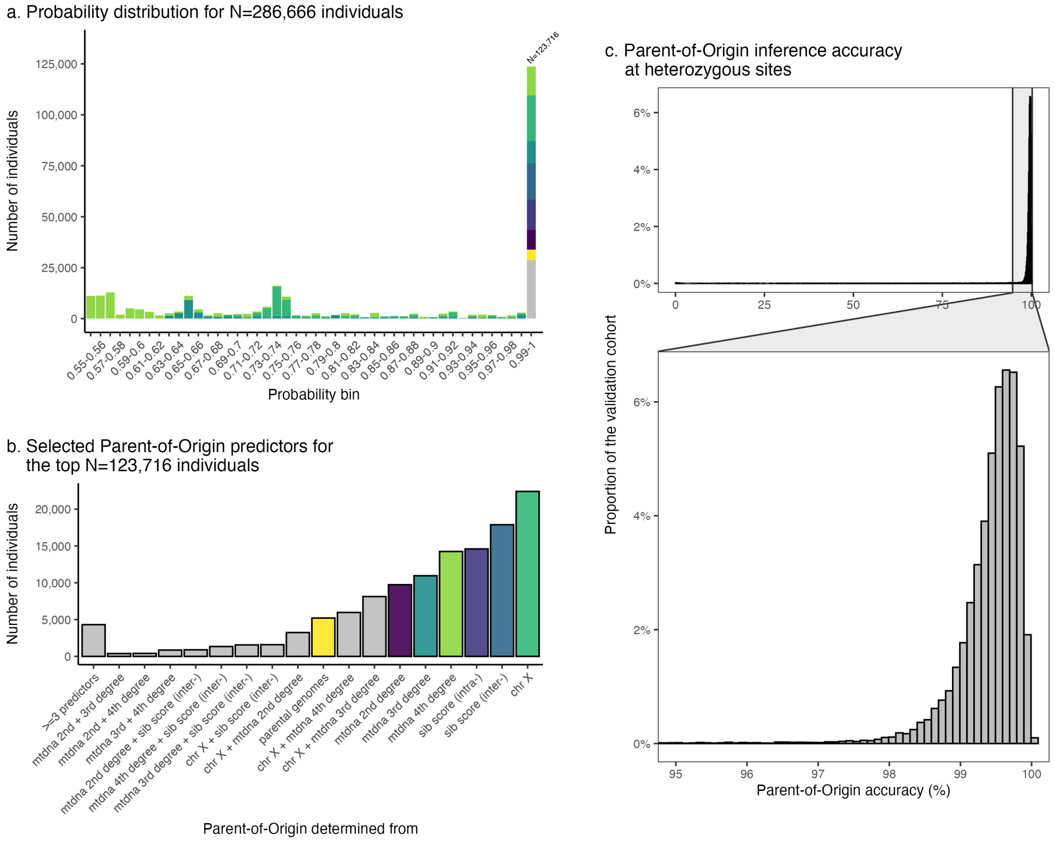

Supplementary Fig. 14 | Selected parent-of-origin predictors and error rate. a) Distribution of PofO probabilities for N=286,666 individuals. The x-axis represents probability bins, and the y-axis shows the number of individuals. Colors correspond to the predictors used for PofO determination, as indicated in panel (b). **b**) Distribution of selected PofO predictors for individuals having a PofO probability greater than 0.99. **c**) Distribution of the PofO inference accuracy. Errors were computed at heterozygous site only by comparing the PofO determined using our approach to the one obtained from parental genomes. We found an average accuracy of 97.94%. This rate is impacted by individuals for which entire haplotypes are incorrect, resulting from inter-chromosomal phasing errors, totaling 64 haplotypes (in the validation cohort). These are likely due to the presence of relatives sharing both paternal and maternal IBD segments, which may be due to consanguinity, and were not filtered out during the construction of the validation cohort. As a result, only a few individuals' haplotypes decrease the global accuracy, and most individuals have a PofO correctly assigned. Indeed, the majority of haplotypes (83.9%) exhibit accuracy above 99%, and half of the haplotypes have more than 0.52% accuracy.

✉ Corresponding authors: Robin J. Hofmeister and Zoltán Kutalik

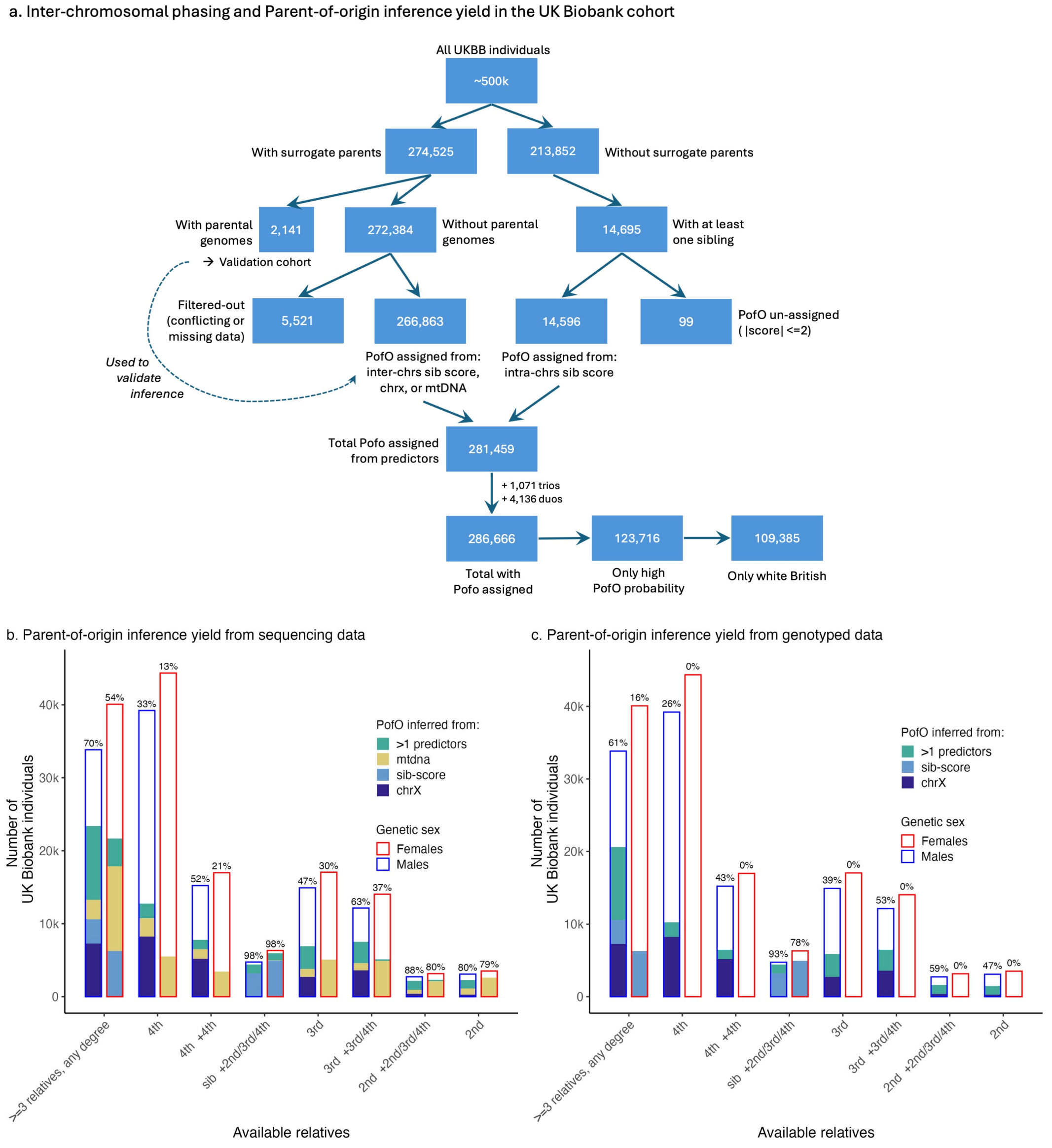

Supplementary Fig. 15 | Overview of inter-chromosomal phasing and parent-of-origin inference yield in the UK Biobank cohort. **a**) Flowchart depicting the stepwise selection of individuals for parent-of-origin inference. Of the approximately 500,000 UK Biobank participants, 274,525 had at least one identified surrogate parent, allowing PofO assignment through inter-chromosomal phasing combined with chromosome X sharing, mitochondrial DNA, or sibling-based crossover inference. An additional 14,695 individuals without surrogate parents had at least one sibling, enabling PofO inference from intra-chromosomal sibling-based crossover scores. After quality control and filtering (e.g., missing or conflicting data), a total of 286,666 individuals had an assigned PofO, including 123,716 with high-confidence PofO probabilities (¿0.99). Of these, the final analysis cohort included 109,385 white British individuals. Yield of Parent-of-Origin inference in the UK Biobank **b**) using WGS data and **c**) using SNP-array data. Number of UK Biobank individuals (y-axis) stratified by available relatives (x-axis). The total bar represent the total number of individuals (y-axis) in each relative category (x-axis). The fill portion represent the number of individuals with PofO inferred from each predictors (colors), with the cumulative proportion indicated on top of each bar. Males and females are also represented separately since chromosome X can be used only in male individuals. As a result, when using only SNP-array data, female individuals can have their PofO inferred only when having a sibling.

✉ Corresponding authors: Robin J. Hofmeister and Zoltán Kutalik

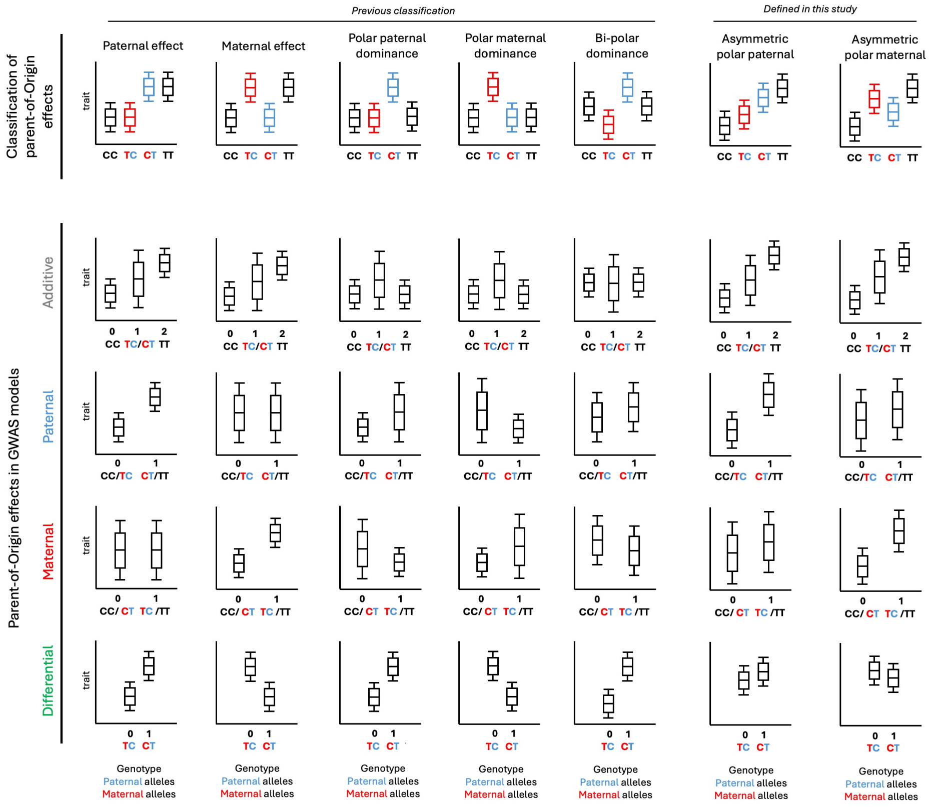

Supplementary Fig. 16 | Classification and detection of parent-of-origin effects in GWAS. Top panel illustrates the classification of POEs[29] and shows the variation in trait values (y-axis) across genotypes and parental origin of alleles (x-axis). Maternal alleles are shown in red, and paternal alleles are shown in blue. Boxplots represent the distribution of trait values for different genotypes: black boxes for homozygotes, red boxes for maternal heterozygotes, and blue boxes for paternal heterozygotes. Each boxplot includes the 25th, 50th (median), and 75th percentiles, with whiskers extending to the minimum and maximum values. **Bottom panels** show the interpretation of the different POEs (x-axis) using various GWAS models (y-axis), including additive, paternal, maternal, and differential models. In this study, we identified POEs using the differential GWAS model.

✉ Corresponding authors: Robin J. Hofmeister and Zoltán Kutalik

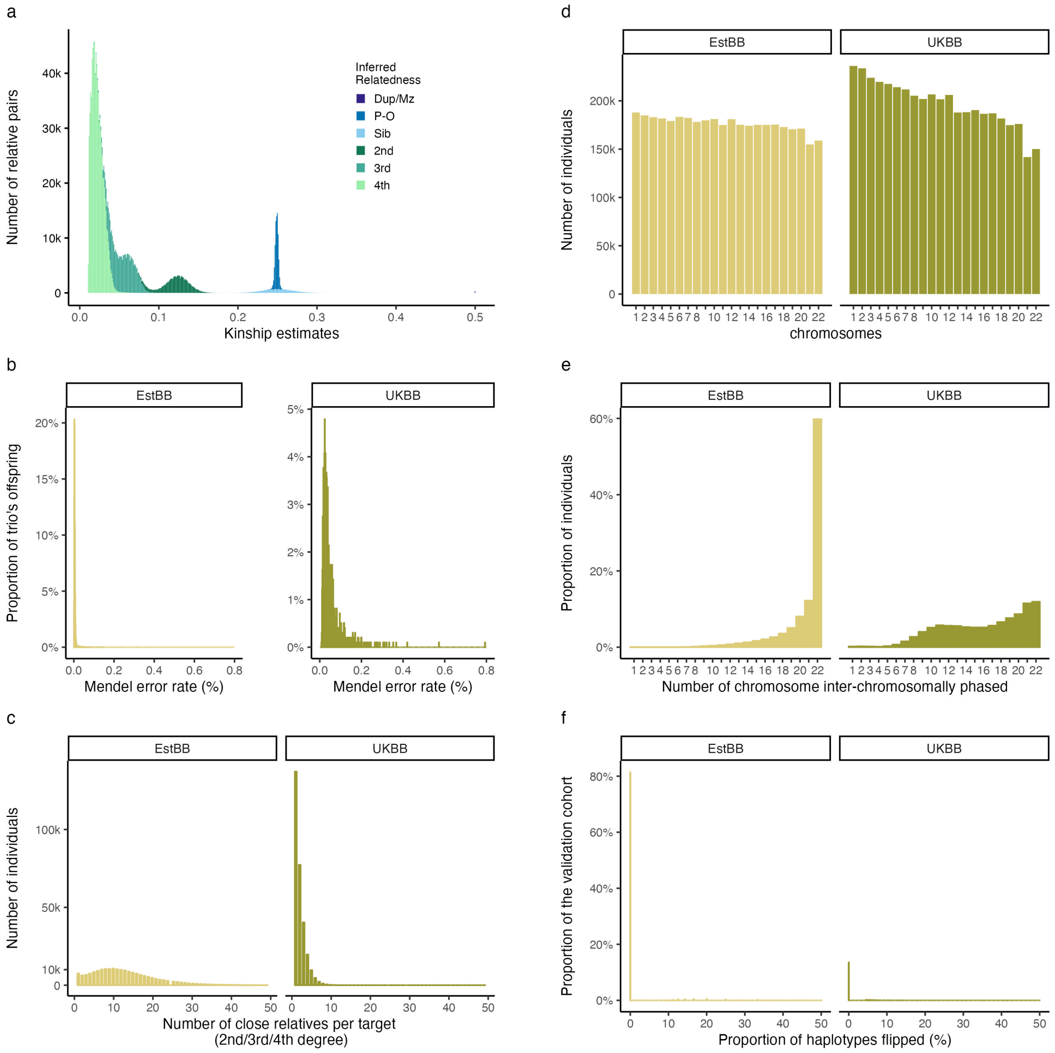

Supplementary Fig. 17 | Overview of relatedness metrics and inter-chromosomal phasing in the Estonian Biobank (EstBB), with comparisons to the UK Biobank (UKBB). a) Distribution of inferred relatedness across the EstBB cohort. Dup/MZ=monozygotic twins; P-O=parent-offspring; Sib=sibling; 2nd=2nd degree relative pairs; 3rd=3rd degree relative pairs; 4th=4th degree relative pairs. Relatedness was inferred using the KING software v2.2.7[30]. **b**) Distribution of Mendel error in trio's offspring for both the EstBB and UKBB cohorts. **c**) Distribution of the number of close relative per individual. **d**) Distribution of the number of individuals (y-axis) with a given chromosome (x-axis) successfully included in the inter-chromosomal phasing. **e**) Distribution of the number of individuals with N chromosomes (x-axis) successfully included in the inter-chromosomal phasing. **f**) Distribution of inter-chromosomal phasing error rate in the validation cohorts, calculated by comparing phasing results from surrogate parents to those obtained using parental genomes as the ground truth.

✉ Corresponding authors: Robin J. Hofmeister and Zoltán Kutalik

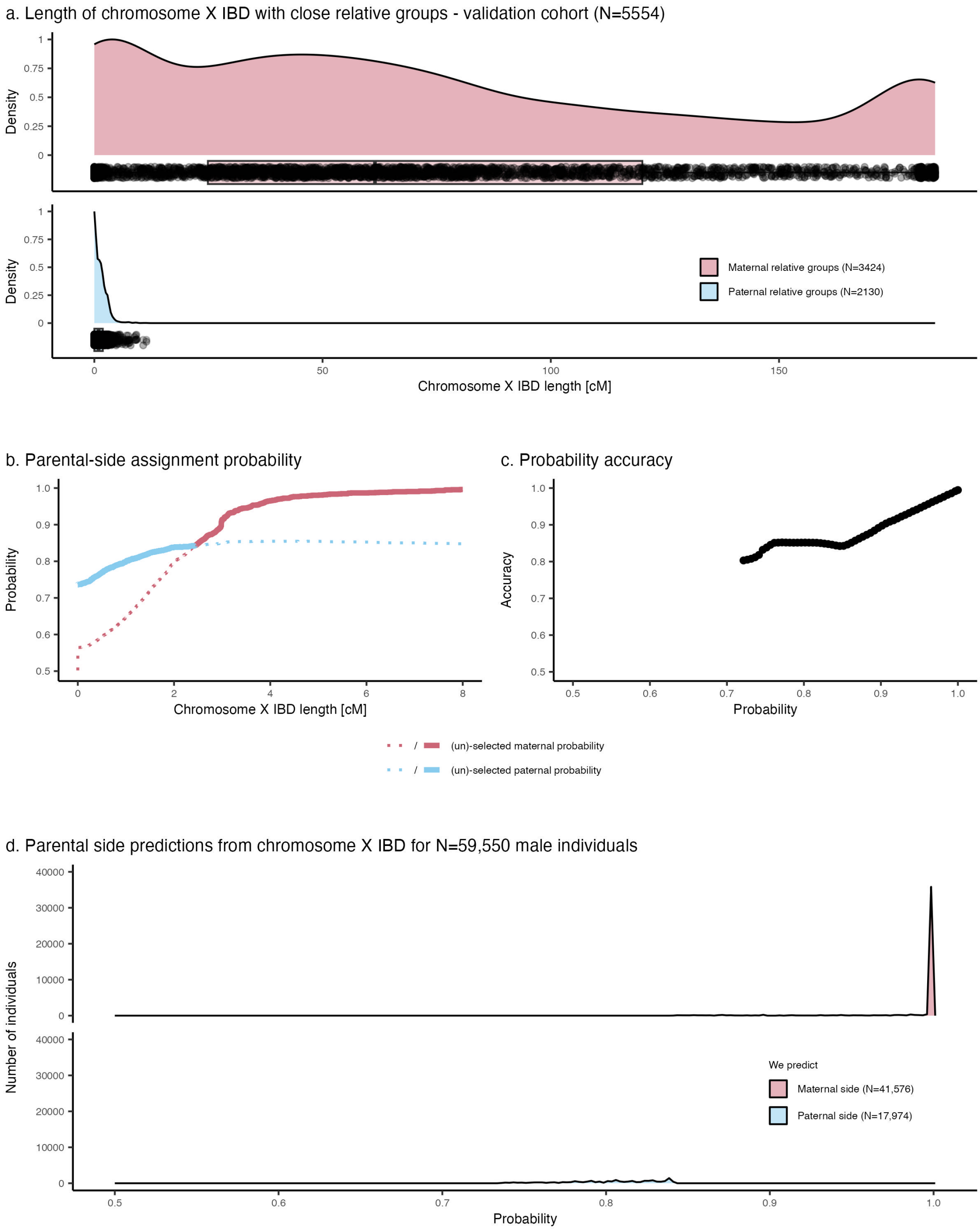

Supplementary Fig. 18 | Parental side assignment from chromosome X IBD analysis in male individuals in the Estonian Biobank. **a**) Density distribution of chromosome X IBD segment length in centimorgan (cM) between targets and surrogate father (blue) and targets and surrogate mother (red) across the validation cohort's male individuals. Boxes indicate the interquartile range (IQR), with the bottom and top of the box representing the 25th (Q1) and 75th (Q3) percentiles, respectively. The horizontal line within the box represents the median (50th percentile). Whiskers extend to the smallest and largest values within $Q1 - 1.5 \times IQR$ and $Q3 + 1.5 \times IQR$. Each point represent a target-relative pair. **b**) Parental side probabilities depending on chromosome X IBD segment length derived from the validation cohort. Selected and unselected maternal (red) and paternal (blue) probabilities for a given x-axis value are indicated by solid and dotted lines, respectively. **c**) Accuracy of parental side predictions (y-axis) as a function of the parental side probability (x-axis). **d**) Distribution of maternal (red) and paternal (blue) side assignment probabilities derived chromosome X IBD across 59,550 Estonian Biobank male individuals.

✉ Corresponding authors: Robin J. Hofmeister and Zoltán Kutalik

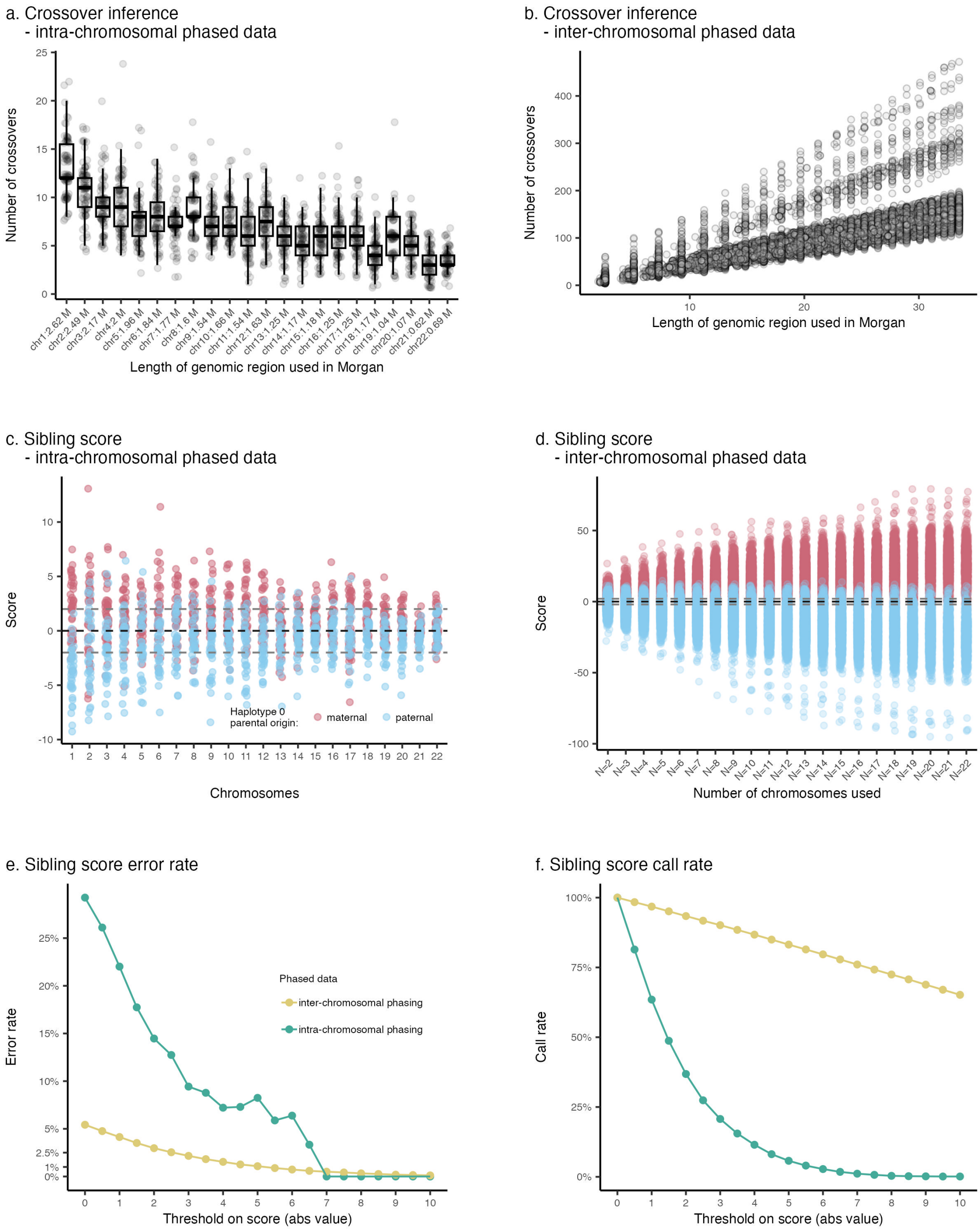

Supplementary Fig. 19 | Evaluation of sibling scores from intra- and inter-chromosomal phasing in the Estonian Biobank validation cohort. a) Distribution of the number of inferred crossovers (y-axis) per chromosome (x-axis) derived from intra-chromosomally phased data. **b**) Distribution of the number of inferred crossovers (y-axis) relative to the genomic length used (in Morgans, x-axis) derived from inter-chromosomally phased data. **c**) Distribution of sibling scores (y-axis) per chromosome (x-axis) using intra-chromosomally phased data. Maternal and paternal haplotypes are color-coded in red and blue, respectively. **d**) Distribution of sibling scores (y-axis) relative to the number of chromosomes included in inter-chromosomally phased data (x-axis). **e**) Error rates (y-axis) of sibling scores for varying score thresholds (absolute values, x-axis). **f**) Call rates (y-axis) of sibling scores for varying score thresholds (absolute values, x-axis), showing the proportion of individuals meeting the threshold criteria.

✉ Corresponding authors: Robin J. Hofmeister and Zoltán Kutalik

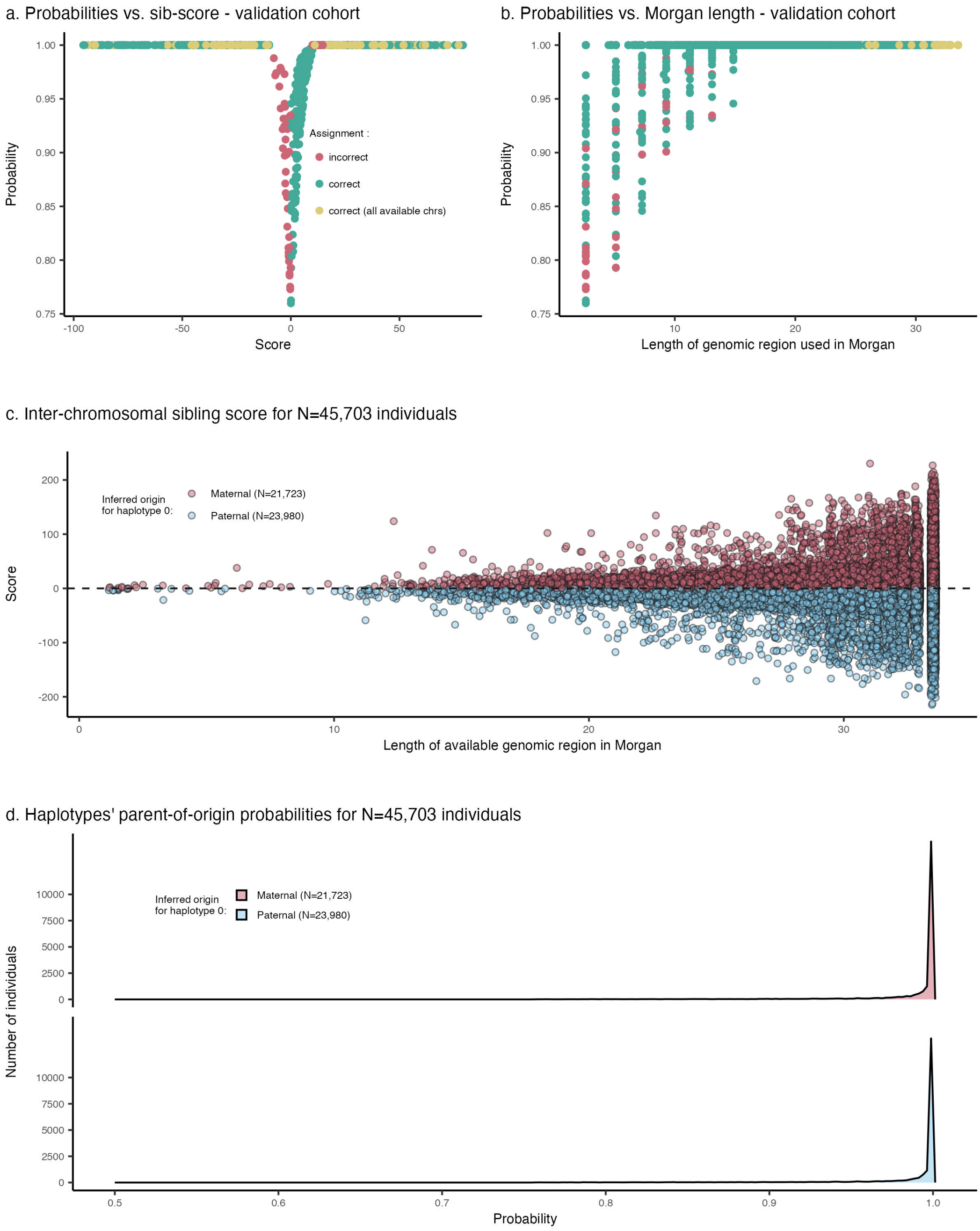

Supplementary Fig. 20 | Validation and derivation of parent-of-origin probabilities using sibling scores from inter-chromosomal phased data in the Estonian Biobank. a) PofO probabilities (y-axis) as a function of sibling scores (x-axis) in the validation cohort. Each dot represents a specific configuration (an individual with a given number of chromosomes used). We varied the number of chromosome used per individual to assess the accuracy of different configurations. Red dots indicate incorrect parental assignments, green dots indicate correct assignments, and yellow dots denote correct assignments using the maximum available chromosomes (N_{max}, corresponds to the full set of inter-chromosomally phased chromosome for a given individual). No errors were observed when N_{max} chromosomes were used. **b**) PofO probabilities (y-axis) plotted against the total available genomic length in Morgans (x-axis) for sibling score calculation in the validation cohort. Each configuration is represented as a dot (color-coded as in panel a). Increasing genomic length results in higher assignment accuracy, with perfect accuracy achieved at N_{max} (yellow dots). **c**) Sibling scores (y-axis) derived for N=45,703 individuals across available genomic lengths (x-axis). **d**) Distribution of PofO probabilities for 45,703 Estonian Biobank individuals.

✉ Corresponding authors: Robin J. Hofmeister and Zoltán Kutalik

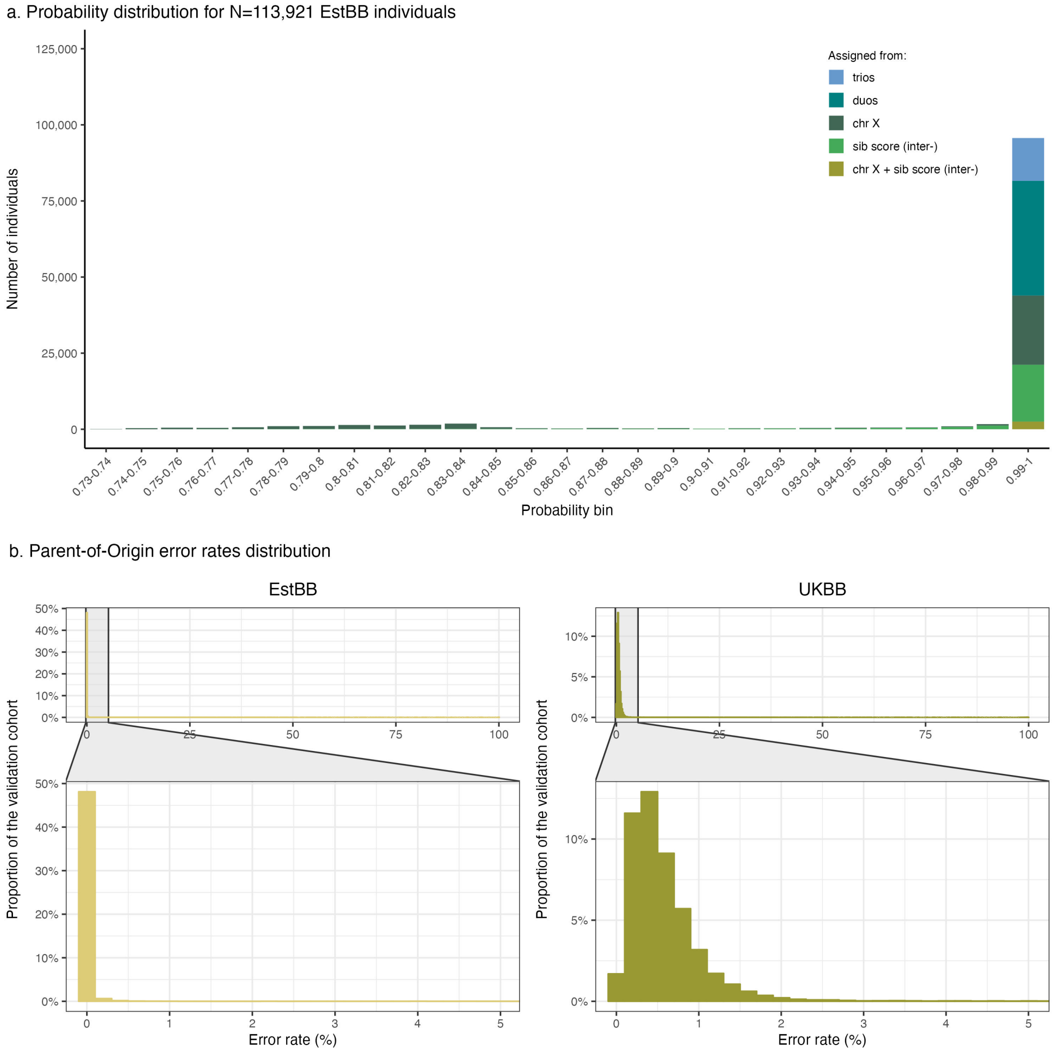

Supplementary Fig. 21 | Parent-of-origin probability distribution and error rates in the Estonian Biobank. a) Distribution of PofO probabilities for N=113,921 individuals in the Estonian Biobank, color-coded by the source of inference (e.g., chromosome X data, sibling score from inter-chromosomal phased data, duos, and trios). Most individuals exhibit high PofO probabilities, reflecting the accuracy of the approach. **b)** Comparison of PofO error rate distributions between the Estonian Biobank (EstBB) and UK Biobank (UKBB) validation cohorts. The EstBB shows consistently lower error rates, likely attributed to the higher number of close relatives per individual, which enhances IBD detection and improves inter-chromosomal phasing accuracy. Zoomed panels show the error rates below 5%, highlighting the robustness of PofO inference in both biobanks.

✉ Corresponding authors: Robin J. Hofmeister and Zoltán Kutalik

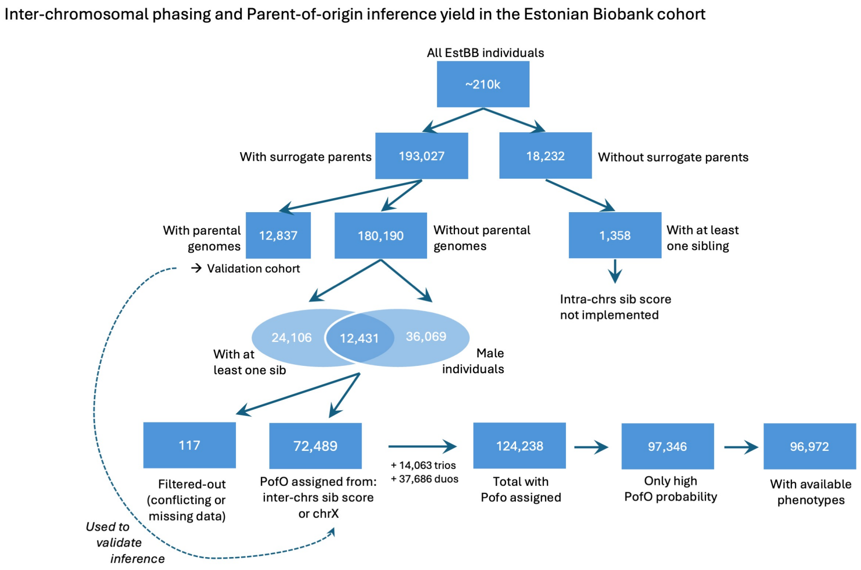

Supplementary Fig. 22 | Overview of inter-chromosomal phasing and parent-of-origin inference yield in the Estonian Biobank cohort. Flowchart depicting the stepwise selection of individuals for parent-of-origin inference. Of the approximately 210,000 Estonian Biobank participants, 193,027 had at least one identified surrogate parent, allowing PofO assignment through inter-chromosomal phasing combined with chromosome X sharing or sibling-based crossover inference. An additional 1,358 individuals without surrogate parents had at least one sibling, enabling PofO inference from intra-chromosomal sibling-based crossover scores. However, given the relatively small sample size, we chose to not implement this approach in the EstBB cohort. After quality control and filtering (e.g., missing or conflicting data), a total of 124,238 individuals had an assigned PofO, including 97,346 with high-confidence PofO probabilities (¿0.99). Of these, the final analysis cohort included 96,972 individuals for which phenotypic measurements were available.

✉ Corresponding authors: Robin J. Hofmeister and Zoltán Kutalik

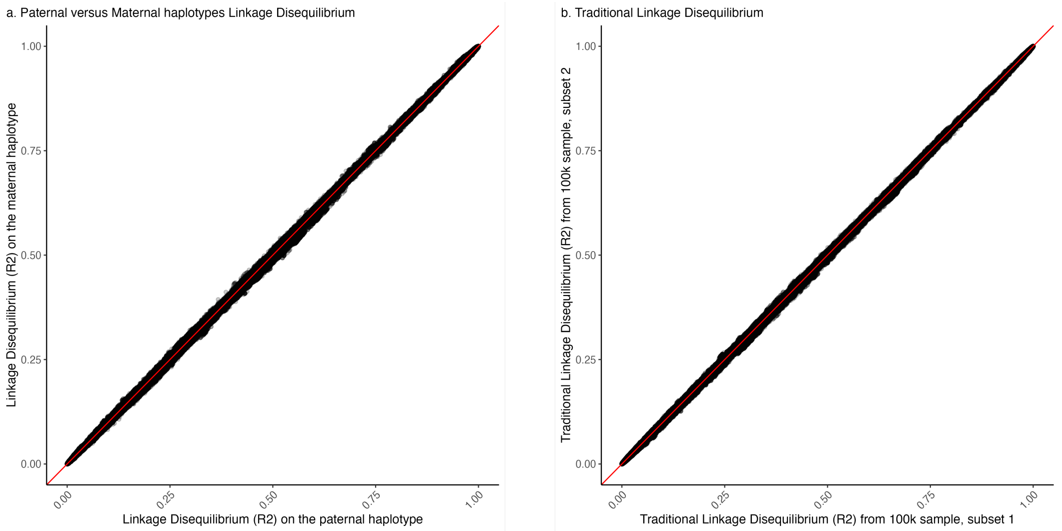

Supplementary Fig. 23 | Haplotype-based Linkage disequilibrium. **a**) Linkage Disequilibrium computed from maternal (y-axis) *vs.* paternal (x-axis) haplotypes for 109,385 white British individuals. Each point represent a genetic variant. **b**) Traditional Linkage Disequilibrium computed from the genotype data of two different subset of 100,000 white British individuals. Each point represent a genetic variant.

✉ Corresponding authors: Robin J. Hofmeister and Zoltán Kutalik

Supplementary Tables

TRAIT	N	N males	N females	Category
Standing height	109385	58572	50813	growth traits
Hip circumference	109385	58572	50813	growth traits
Waist circumference	109385	58572	50813	growth traits
Trunk fat-free mass	109385	58572	50813	growth traits
Trunk fat percentage	109385	58572	50813	growth traits
Arm fat-free mass	109385	58572	50813	growth traits
Arm fat percentage	109385	58572	50813	growth traits
Leg fat-free mass	109385	58572	50813	growth traits
Leg fat percentage	109385	58572	50813	growth traits
Whole body water mass	109385	58572	50813	growth traits
Body fat percentage	109385	58572	50813	growth traits
Body mass index	109385	58572	50813	growth traits
Birth weight	109385	58572	50813	growth traits
Vitamin D	109385	58572	50813	metabolism traits
Urate	109385	58572	50813	metabolism traits
Triglycerides	109385	58572	50813	metabolism traits
Total protein	109385	58572	50813	metabolism traits
Testosterone	109385	58572	50813	metabolism traits
SHBG	109385	58572	50813	metabolism traits
Phosphate	109385	58572	50813	metabolism traits
Oestradiol	109385	58572	50813	metabolism traits
Lipoprotein A	109385	58572	50813	metabolism traits
LDL direct	109385	58572	50813	metabolism traits
IGF-1	109385	58572	50813	metabolism traits
HDL cholesterol	109385	58572	50813	metabolism traits
Glycated haemoglobin	109385	58572	50813	metabolism traits
Glucose	109385	58572	50813	metabolism traits
Cystatin C	109385	58572	50813	metabolism traits
Cholesterol	109385	58572	50813	metabolism traits
Calcium	109385	58572	50813	metabolism traits
Urea	109385	58572	50813	metabolism traits
Apoliprotein B	109385	58572	50813	metabolism traits
Apoliprotein A	109385	58572	50813	metabolism traits
Basal metabolic rate	109385	58572	50813	metabolism traits
Type 2 diabetes	9189 (99007)	6265 (51513)	2925 (47495)	metabolism traits
Telomere length	109355	58552	50803	other traits
Sodium in urine	109372	58565	50807	other traits
Creatinine	109379	58568	50811	other traits
Microalbumin in urine	109379	58568	50811	other traits
Total bilirubin	109385	58572	50813	other traits
Rheumatoid factor	109385	58572	50813	other traits
Gamma glutamyltransferase	109385	58572	50813	other traits
C-reactive protein	109385	58572	50813	other traits
Creatinine	109385	58572	50813	other traits
Direct bilirubin	109385	58572	50813	other traits
Aspartate aminotransferase	109385	58572	50813	other traits
Alanine aminotransferase	109385	58572	50813	other traits
Alkaline phosphatase	109385	58572	50813	other traits
Albumin	109385	58572	50813	other traits
Reticulocyte count	109385	58572	50813	other traits
Nucleated red blood cell count	109385	58572	50813	other traits
Basophill count	109385	58572	50813	other traits
Eosinophill count	109385	58572	50813	other traits
Neutrophill count	109385	58572	50813	other traits
Monocyte count	109385	58572	50813	other traits
Lymphocyte count	109385	58572	50813	other traits
Platelet count	109385	58572	50813	other traits
Red blood cell	109385	58572	50813	other traits
White blood cell	109385	58572	50813	other traits

Supplementary Table 1 | UK Biobank traits selected for this study.
TRAIT: phenotype name; N: number of individuals (selected as (i) self-reported as white British, confirmed by PCA, according to UK Biobank field 22006; (ii) having a PofO probability > 0.99, as defined for the differential GWAS[2]). For type 2 diabetes, we indicated cases and controls. TS ratio: Relative leucocyte telomere length; SHBG: Sex Hormone-Binding Globulin.

✉ Corresponding authors: Robin J. Hofmeister and Zoltán Kutalik

				Published studies					Current study				
	TRAIT	RSID	ID	BETA PAT	P PAT	BETA MAT	P MAT	P DIFF	BETA PAT	P PAT	BETA MAT	P MAT	P DIFF
Kim et al., HGG Adv 2021	**HDL-C**	**rs12154627**	**7_130422934_T_C**	**0.0300**	**6.40E-02**	**0.1300**	**2.10E-16**	**3.10E-06**	**0.0139**	**2.76E-02**	**0.0463**	**1.93E-13**	**6.20E-04**
	**triglyceride**	**rs12154627**	**7_130422934_T_C**	**-0.0100**	**5.60E-01**	**-0.1000**	**1.40E-09**	**1.30E-04**	**-0.0151**	**2.69E-02**	**-0.0465**	**8.94E-12**	**1.00E-03**
	**platelets**	**rs10146962 [2]**	**14_101170540_T_C**	**0.0400**	**6.30E-02**	**-0.1000**	**2.30E-11**	**7.80E-10**	**-0.0180**	**9.17E-03**	**-0.0964**	**3.73E-44**	**9.22E-16**
Zoledziewska et al., Nat Genet 2015	**Height**	**rs143840904 [1]**	**11_2813322_C_T**	**0.0021**	**9.65E-01**	**-0.2740**	**3.98E-08**	**7.55E-05**	**-0.0226**	**1.37E-01**	**-0.1831**	**9.13E-32**	**5.21E-13**
	**Height**	**rs2075870 [1]**	**11_2790019_G_A**	**-0.0172**	**7.93E-01**	**-0.2730**	**6.97E-08**	**2.00E-04**	**-0.0246**	**8.31E-02**	**-0.1439**	**3.00E-23**	**3.89E-09**
	**Height**	**rs149658560 [1]**	**11_2767262_G_A**	**-0.0121**	**8.18E-01**	**-0.2970**	**2.93E-07**	**3.00E-04**	**-0.0066**	**6.13E-01**	**-0.0917**	**6.47E-12**	**7.09E-06**
Granot-Hershkovitz et al., EJHG 2020	Height	rs1042136	6_33048628_A_C	-0.0230	1.55E-08	0.0050	4.21E-01	1.39E-04	-0.0160	3.54E-03	-0.0062	2.57E-01	1.90E-01
Benonisdottir et al., Nat Comm 2016	**Height**	**rs147239461**	**11_1986402_G_T**	**-0.1200**	**5.90E-13**	**0.0560**	**9.40E-04**	**1.20E-13**	**-0.0484**	**2.80E-05**	**0.0395**	**4.92E-04**	**4.89E-08**
	Height	rs7482510	11_2190591_C_G	-0.0650	5.10E-11	0.0180	7.60E-02	4.70E-09	-0.0189	2.71E-04	0.0002	9.71E-01	4.15E-03
	**Height**	**rs143840904 [1]**	**11_2813322_C_T**	**0.0570**	**4.20E-02**	**-0.2600**	**2.00E-17**	**1.60E-14**	**-0.0226**	**1.37E-01**	**-0.1831**	**9.13E-32**	**5.21E-13**
	**Height**	**rs41286560**	**14_101349454_G_T**	**-0.1200**	**2.20E-08**	**0.0670**	**1.70E-03**	**7.40E-10**	**-0.0519**	**5.03E-05**	**0.0168**	**1.87E-01**	**1.46E-04**
Kong et al., Nature 2009	**T2D**	**rs2334499**	**11_1696849_C_T**	**1.35 (OR)**	**4.70E-10**	**0.86 (OR)**	**2.00E-03**	**4.10E-11**	**0.1391**	**2.19E-07**	**-0.0737**	**6.05E-03**	**2.16E-08**
Hofmeister et al., Nat Comm 2022	**telomere length**	**rs2735940**	**5_1296486_A_G**	**0.0088**	**4.60E-01**	**-0.1215**	**2.10E-19**	**4.30E-13**	**0.014**	**4.59E-02**	**-0.100**	**3.12E-49**	**2.84E-32**
	**platelets**	**rs59228823 [2]**	**14_101185187_G_C**	**-0.0110**	**5.80E-01**	**-0.1230**	**6.80E-16**	**2.30E-08**	**-0.015**	**4.68E-02**	**-0.104**	**2.14E-42**	**2.42E-16**
Juliusdottir et al., Nat Genet 2021 *	birth weight	rs9855896	3_14287150_A_G	0.039	4.90E-05	-0.017	1.30E-01	1.65E-04	-0.009	4.29E-01	0.019	8.74E-02	7.59E-02
	birth weight	rs6575803	14_101257755_C_T	0.094	8.30E-17	0.009	5.20E-01	6.00E-07	-0.049	2.03E-03	-0.014	3.75E-01	1.48E-01
	birth weight	rs2296528	20_57274151_G_C	0.037	3.80E-06	-0.012	2.10E-01	4.72E-05	0.032	1.64E-03	0.022	3.45E-02	4.68E-01
	birth weight	rs76094073	6_109288036_C_G	-0.007	5.20E-01	0.06	1.60E-05	2.80E-04	0.042	2.89E-03	0.039	5.84E-03	8.63E-01
	birth weight	rs2529415	7_50736662_C_T	-0.004	6.70E-01	0.039	1.50E-04	7.86E-04	-0.001	9.25E-01	-0.017	8.40E-02	2.39E-01
	birth weight	rs2901307	10_124128443_C_T	0.001	8.50E-01	0.044	2.20E-06	7.03E-05	0.026	5.52E-03	0.031	7.07E-04	6.63E-01
	birth weight	rs231848	11_2735557_A_G	-0.003	7.00E-01	0.051	8.10E-08	9.70E-06	0.005	6.19E-01	-0.019	4.41E-02	7.32E-02
	birth weight	rs2928148	15_41401550_G_A	0.002	8.00E-01	0.045	1.30E-06	1.62E-04	0.016	8.77E-02	0.026	7.09E-03	4.88E-01

Supplementary Table 2 | Replication of previously published parent-of-origin effects.
TRAIT: phenotype name; RSID: variant rs id; ID: chromosome_position(hg19)_reference allele_alternative allele; BETA and P denote effect sizes and P-values; PAT, MAT, and DIFF denote paternal, maternal and differential tests; bold: associations replicated in our study; [1] and [2]: represent the same association with standing height and platelet count, respectively; *: study for which P_D was computed from a z-score (see Methods).

Chromosome	Region start	Region end	Genes overlapping region
chr1	68011644	69016459	ARHI
chr2	80015483	81031487	LRRTM1
chr4	89117065	90118980	NAPIL5
chr6	56682421	57691161	PRIM2
chr6	143761436	144885734	PLAGL1,HYMAI
chr6	159890130	161376014	IGF2R,SLC22A2,SLC22A3
chr7	50154712	51361157	GRB10
chr7	93714535	95425726	SGCE,PEG10,PPP1R9A
chr7	96149701	97154142	DLX5
chr7	129432993	130918860	CPA4,MEST,KLF14
chr8	949568	2156642	DLGAP2
chr8	140113081	141215299	KCNK9
chr11	1516405	3686539	H19,IGF2,IGF2AS,INS,KCNQ1,KCNQ1DN,CDKN1C,SLC22A1LS,SLC22A1L,PHLDA2,OSBPL5
chr11	5911654	7479277	SMPD1,ZNF215
chr11	31909324	32957086	WT1
chr14	100693252	101827367	DLK1,MEG3
chr15	23310105	24432449	MKRN3,MAGEL2,NDN
chr15	24700069	26608348	SNURF,IPW,UBE3A,ATP10A
chr16	2986109	3993490	ZNF597
chr18	44054572	45056448	TCEB3C
chr19	56785919	57852075	ZIM2,PEG3
chr20	35649606	36652091	NNAT
chr20	41643052	42670534	L3MBTL
chr20	56914783	57986249	NESP55,GNAS1

Supplementary Table 3 | Imprinted regions.

Imprinted regions extracted from Kong *et al.*[6] and lifted over to hg19.

✉ Corresponding authors: Robin J. Hofmeister and Zoltán Kutalik

CHR	POS	SNP ID	A0	A1	A1FREQ	BETA PAT	SE PAT	P PAT	BETA MAT	SE MAT	P MAT	BETA DIFF	SE DIFF	P DIFF	BETA ADD	SE ADD	P ADD	TRAIT
11	1918083	rs7105710	T	C	0.546	0.001	0.005	8.18E-01	-0.013	0.005	8.00E-03	0.014	0.007	4.10E-02	-0.006	0.003	8.70E-02	Sitting height
11	2040272	rs77708343	A	G	0.039	-0.055	0.012	8.51E-06	0.046	0.012	1.21E-04	-0.101	0.017	4.17E-09	-0.003	0.009	6.95E-01	Sitting height
11	2813322	rs143840904	C	T	0.019	-0.044	0.018	1.30E-02	-0.131	0.018	6.61E-13	0.087	0.025	3.80E-04	-0.086	0.013	1.06E-11	Sitting height

Supplementary Table 4 | Replication of standing height association with sitting height.

CHR: chromosome; POS: genetic position (hg19); SNP ID: variant rs id; A0: reference allele; A1: assessed allele; A1FREQ: A1 allele frequency. BETA, SE and P denote effect sizes, standard errors and P-values; PAT, MAT, DIFF and ADD denote paternal, maternal, differential and additive tests. TRAIT: phenotype name.

CHR	POS	SNP ID	A0	A1	A1FREQ	BETA PAT	SE PAT	P PAT	BETA MAT	SE MAT	P MAT	BETA DIFF	SE DIFF	P DIFF	BETA ADD	SE ADD	P ADD	TRAIT	Status	Locus
2	54422548	rs7562908	A	G	0.026	0.148	0.021	1.06E-12	0.043	0.021	3.86E-02	-0.104	0.029	4.17E-04	-0.099	0.015	2.80E-11	TS ratio	novel	ACYP2
3	46058335	rs6809223	T	C	0.734	-0.049	0.007	1.62E-11	-0.015	0.007	4.20E-02	-0.034	0.010	8.80E-04	-0.032	0.005	5.24E-10	Basophill count	novel	CCR1, CCR3, XCR1
3	156795468	rs13322435	A	G	0.403	-0.072	0.009	1.05E-14	-0.038	0.009	3.15E-05	-0.033	0.013	1.12E-02	-0.055	0.007	4.24E-17	Birth weight	novel	TIPARP
5	72399063	rs10223082	T	C	0.667	-0.052	0.008	9.54E-12	-0.014	0.008	6.50E-02	-0.037	0.011	5.12E-04	-0.033	0.005	9.51E-10	Calcium	novel	FCHO2
7	73026378	rs17145750	C	T	0.162	0.015	0.009	9.68E-02	0.063	0.009	9.07E-13	-0.048	0.013	1.18E-04	0.039	0.006	4.60E-10	IGF-1	novel	MLXIPL
11	2157793	rs3213217	A	T	0.236	0.096	0.008	1.44E-34	0.058	0.008	2.01E-13	0.038	0.011	5.72E-04	0.077	0.006	7.74E-44	IGF-1	novel	IGF2

Supplementary Table 5 | Parent-of-origin effect within additively associated regions, per phenotype.
CHR: chromosome; POS: genetic position (hg19); SNP ID: variant rs id; A0: reference allele; A1: assessed allele; A1FREQ: A1 allele frequency. BETA, SE and P denote effect sizes, standard errors and P-values; PAT, MAT, DIFF and ADD denote paternal, maternal, differential and additive tests. TRAIT: phenotype name.

	CHR	POS	SNP ID	A0	A1	A1FREQ	BETA PAT	SE PAT	P PAT	BETA MAT	SE MAT	P MAT	BETA DIFF	SE DIFF	P DIFF	BETA ADD	SE ADD	P ADD	PHECODE	TRAIT
a	5	1296486	rs2735940	A	G	0.493	0.014	0.007	4.59E-02	-0.100	0.007	3.12E-49	0.113	0.010	2.84E-32	-0.043	0.005	1.71E-19	22191	TS ratio
	11	1702929	rs10838787	G	A	0.425	0.050	0.007	2.34E-14	-0.028	0.007	1.80E-05	0.078	0.009	2.77E-17	0.011	0.005	1.76E-02	30750	Glycated haemoglobin
	11	1998031	rs170102	G	A	0.346	-0.041	0.006	5.96E-11	0.026	0.006	3.19E-05	-0.067	0.009	5.98E-14	-0.007	0.004	9.47E-02	30720	Cystatin C
	11	2003944	2003944	T	G	0.656	0.030	0.006	2.53E-07	-0.024	0.006	2.68E-05	0.054	0.008	4.94E-11	0.003	0.004	5.04E-01	30700	Creatinine
	11	2813322	rs143840904	C	T	0.019	-0.023	0.015	1.37E-01	-0.183	0.016	9.13E-32	0.152	0.021	5.21E-13	-0.100	0.011	2.56E-20	50	Standing height
	14	101185187	rs59228823	G	C	0.245	-0.010	0.008	2.04E-01	-0.092	0.008	3.87E-34	0.082	0.011	1.92E-14	-0.051	0.005	2.36E-21	30080	Platelet count
b	7	130016470	rs62471721	G	A	0.351	-0.023	0.007	9.70E-04	0.033	0.007	1.57E-06	-0.057	0.010	6.51E-09	0.005	0.005	2.85E-01	30870	Triglycerides
	11	1702929	rs10838787	G	A	0.425	0.136	0.027	3.88E-07	-0.091	0.027	7.55E-04	0.228	0.038	1.87E-09	0.023	0.019	2.26E-01	130708	Type 2 diabetes
	11	2040272	rs77708343	A	G	0.040	-0.052	0.011	1.28E-06	0.033	0.011	2.06E-03	-0.085	0.015	1.80E-08	-0.009	0.008	2.29E-01	23113	Leg fat-free mass
	20	57216538	rs80116540	A	G	0.075	0.035	0.010	4.38E-04	-0.043	0.010	2.04E-05	0.078	0.014	3.39E-08	-0.004	0.007	6.09E-01	23119	Arm fat percentage
	1	40593883	rs145976519	A	C	0.022	0.096	0.031	2.00E-03	-0.155	0.031	5.96E-07	0.251	0.043	3.96E-09	-0.030	0.022	1.78E-01	20022	Birth weight
	1	81185936	rs2152857	A	G	0.024	-0.074	0.018	3.08E-05	0.066	0.018	1.96E-04	0.143	0.025	1.58E-08	0.007	0.014	6.03E-01	30840	Total bilirubin
	2	863453	rs73161490	T	G	0.162	-0.035	0.009	4.70E-05	0.031	0.009	2.40E-04	-0.066	0.012	4.89E-08	-0.002	0.006	7.99E-01	30770	IGF-1
	3	108030034	rs17241163	A	G	0.091	0.087	0.026	6.55E-04	-0.116	0.026	7.34E-06	0.202	0.036	2.08E-08	-0.014	0.018	4.58E-01	30800	Oestradiol
	5	127663392	5 127663392 G A	G	A	0.139	0.024	0.010	1.28E-02	-0.052	0.010	1.33E-07	0.076	0.014	4.65E-08	-0.014	0.007	4.82E-02	30140	Neutrophill count
	11	114407458	rs138298109	A	C	0.024	-0.082	0.023	3.87E-04	0.091	0.023	7.28E-05	-0.169	0.031	4.03E-08	0.005	0.016	7.52E-01	30510	Creatinine
	14	83180060	14 83180060 A C	A	C	0.120	0.059	0.020	3.43E-03	-0.099	0.020	7.11E-07	0.156	0.028	3.58E-08	-0.021	0.014	1.40E-01	30500	Microalbumin in urine

Supplementary Table 6 | Parent-of-origin effect genome-wide.

CHR: chromosome; POS: genetic position (hg19); SNP ID: variant rs id; A0: reference allele; A1: assessed allele; A1FREQ: A1 allele frequency. BETA, SE and P denote effect sizes, standard errors and P-values; PAT, MAT, DIFF and ADD denote paternal, maternal, differential and additive tests. TRAIT: phenotype name. **a**) POEs robust when correcting for the number of traits tested ($P_D < 1 \times 10^{-9}$). **b**) POEs genome-wide significant at the single trait level ($1 \times 10^{-9} < P_D < 5 \times 10^{-8}$).

CHR	POS	SNP ID	A0	A1	BETA_PAT	SE_PAT	P_PAT	BETA_MAT	SE_MAT	P_MAT	P_DIFF	TRAIT	INITIAL TRAITS WITH POE AT THIS LOCUS
**11**	**1914139**	**rs576603**	**C**	**T**	**-0.048**	**0.015**	**1.70E-03**	**0.041**	**0.015**	**7.26E-03**	**3.29E-05**	**CHSA10**	**Standing height**
**11**	**2040272**	**rs77708343**	**A**	**G**	**-0.118**	**0.039**	**2.29E-03**	**0.071**	**0.038**	**6.32E-02**	**4.40E-04**	**CHSA10**	**Standing height, Basal metabolic rate, Leg fat-free mass, Whole body water mass**
11	2813322	rs143840904	C	T	-0.070	0.056	2.10E-01	-0.321	0.057	2.07E-08	2.70E-03	CHSA10	Standing height
7	130017940	rs4731690	A	G	-0.014	0.009	1.23E-01	-0.010	0.009	2.57E-01	8.13E-01	Birth weight	HDL cholesterol, Triglycerides, SHBG
7	130463192	rs6467315	C	G	0.002	0.009	8.61E-01	0.004	0.009	6.25E-01	8.24E-01	Birth weight	Hip circumference
11	1702929	rs10838787	G	A	-0.029	0.009	2.31E-03	0.007	0.009	4.72E-01	8.04E-03	Birth weight	Type 2 diabetes, Glycated haemoglobin, Glucose
11	2040272	rs77708343	A	G	-0.057	0.024	1.88E-02	0.030	0.024	1.98E-01	9.35E-03	Birth weight	Standing height, Basal metabolic rate, Leg fat-free mass, Whole body water mass
11	2858295	rs2299620	C	T	0.040	0.026	1.19E-01	0.036	0.025	1.47E-01	9.03E-01	Birth weight	Glycated haemoglobin
20	57226079	rs6026426	C	G	-0.020	0.019	3.02E-01	0.030	0.019	1.16E-01	6.16E-02	Birth weight	Trunk fat percentage, Arm fat percentage, Body fat percentage, Leg fat percentage
7	130017940	rs4731690	A	G	0.032	0.013	1.34E-02	0.001	0.013	9.16E-01	1.01E-01	CBSA10	HDL cholesterol, Triglycerides, SHBG
7	130463192	rs6467315	C	G	0.013	0.013	3.16E-01	0.008	0.013	5.41E-01	7.73E-01	CBSA10	Hip circumference
11	1702929	rs10838787	G	A	-0.002	0.013	8.82E-01	-0.013	0.013	3.40E-01	6.03E-01	CBSA10	Type 2 diabetes, Glycated haemoglobin, Glucose
11	2040272	rs77708343	A	G	-0.026	0.034	4.42E-01	0.026	0.033	4.42E-01	2.90E-01	CBSA10	Standing height, Basal metabolic rate, Leg fat-free mass, Whole body water mass
11	2858295	rs2299620	C	T	0.049	0.035	1.62E-01	0.071	0.035	4.32E-02	7.05E-01	CBSA10	Glycated haemoglobin
20	57226079	rs6026426	C	G	0.032	0.027	2.36E-01	-0.043	0.027	1.10E-01	5.15E-02	CBSA10	Trunk fat percentage, Arm fat percentage, Body fat percentage, Leg fat percentage

Supplementary Table 7 | Replication of associations with early childhood traits in the UK Biobank cohort. CHR: chromosome; POS: genetic position (hg19); SNP ID: variant rs id; A0: reference allele; A1: assessed allele; A1FREQ: A1 allele frequency. BETA, SE and P denote effect sizes, standard errors and P-values; PAT, MAT, DIFF and ADD denote paternal, maternal, differential and additive tests. TRAIT: phenotype name. Bold indicate significant POEs; BHSA10: comparative height size at age 10; CBSA10: comparative body size at age 10.

time point	N
6 Weeks	29791
3 Months	41865
6 Months	42346
8 Months	36790
1 Year	36721
16 Months	27595
2 Years	27435
3 Years	27985
5 Years	22667
7 Years	23280
8 Years	17888

Supplementary Table 8 | Number of individuals per time points in the MoBa cohort.

✉ Corresponding authors: Robin J. Hofmeister and Zoltán Kutalik

ID	BMI timepoints	BETA PAT	SE PAT	P PAT	BETA MAT	SE MAT	P MAT	BETA MAT NT	SE MAT NT	P MAT NT	P DIFF	P DIFF MAT	INITIAL TRAITS WITH POE AT THIS LOCUS
rs6467315	6 weeks	-0.019	0.015	2.04E-01	0.071	0.015	1.26E-06	0.028	0.014	4.74E-02	**1.51E-05**	3.47E-02	Hip circumference
	3 months	0.001	0.012	9.62E-01	0.085	0.012	9.46E-12	0.003	0.012	7.74E-01	**1.68E-06**	2.50E-06	
	6 months	0.003	0.012	8.12E-01	0.089	0.012	7.72E-13	0.016	0.012	1.96E-01	**9.43E-07**	2.02E-05	
	8 months	0.012	0.013	3.75E-01	0.087	0.013	6.15E-11	0.009	0.013	4.97E-01	**6.30E-05**	2.23E-05	
	12 months	-0.007	0.013	5.75E-01	0.069	0.013	2.20E-07	0.000	0.013	9.88E-01	**4.85E-05**	1.82E-04	
	16 months	-0.011	0.015	4.51E-01	0.066	0.015	1.24E-05	0.011	0.015	4.39E-01	**2.90E-04**	9.05E-03	
	2 years	-0.017	0.015	2.67E-01	0.057	0.015	1.81E-04	0.017	0.015	2.47E-01	**5.95E-04**	5.81E-02	
	3 years	0.012	0.015	4.17E-01	0.058	0.015	9.60E-05	0.003	0.014	8.61E-01	2.89E-02	7.20E-03	
	5 years	-0.013	0.016	4.38E-01	0.031	0.016	5.77E-02	-0.013	0.016	4.09E-01	5.86E-02	5.24E-02	
	7 years	-0.023	0.016	1.54E-01	0.030	0.016	6.63E-02	-0.008	0.016	6.24E-01	2.10E-02	9.68E-02	
	8 years	-0.004	0.019	8.43E-01	0.026	0.019	1.69E-01	0.024	0.018	1.81E-01	2.66E-01	9.48E-01	
rs10838787	6 weeks	-0.006	0.015	6.76E-01	0.028	0.015	5.61E-02	0.016	0.014	2.57E-01	9.96E-02	5.51E-01	Type 2 diabetes, Glycated haemoglobin, Glucose
	3 months	-0.020	0.013	1.16E-01	0.019	0.013	1.28E-01	0.025	0.012	3.68E-02	2.88E-02	7.28E-01	
	6 months	-0.028	0.013	2.75E-02	0.017	0.013	1.82E-01	0.024	0.012	4.81E-02	1.23E-02	6.86E-01	
	8 months	-0.046	0.013	6.09E-04	0.011	0.013	4.20E-01	0.030	0.013	2.12E-02	2.74E-03	3.12E-01	
	12 months	-0.042	0.013	1.96E-03	0.009	0.013	5.22E-01	0.030	0.013	1.83E-02	8.24E-03	2.42E-01	
	16 months	-0.044	0.015	3.58E-03	0.008	0.015	5.91E-01	0.007	0.015	6.52E-01	1.47E-02	9.40E-01	
	2 years	-0.029	0.015	5.51E-02	0.003	0.015	8.63E-01	-0.001	0.015	9.56E-01	1.39E-01	8.70E-01	
	3 years	-0.038	0.015	1.15E-02	-0.002	0.015	8.84E-01	0.015	0.014	2.92E-01	9.21E-02	4.04E-01	
	5 years	-0.021	0.016	2.11E-01	-0.010	0.016	5.37E-01	0.013	0.016	4.12E-01	6.54E-01	3.11E-01	
	7 years	-0.018	0.017	2.71E-01	-0.004	0.017	8.24E-01	0.024	0.016	1.32E-01	5.35E-01	2.29E-01	
	8 years	-0.001	0.019	9.76E-01	-0.007	0.019	6.91E-01	0.036	0.018	4.67E-02	7.95E-01	9.61E-02	
rs2299620	6 weeks	-0.040	0.040	3.17E-01	-0.025	-0.040	5.36E-01	0.013	0.037	7.30E-01	2.53E-01	4.91E-01	Glycated haemoglobin
	3 months	0.017	0.034	6.17E-01	-0.045	-0.034	1.79E-01	0.034	0.032	2.92E-01	5.60E-01	8.92E-02	
	6 months	0.043	0.034	2.13E-01	-0.015	-0.034	6.67E-01	-0.013	0.032	6.96E-01	5.59E-01	9.65E-01	
	8 months	0.044	0.037	2.35E-01	0.016	-0.036	6.62E-01	0.007	0.034	8.36E-01	2.47E-01	8.62E-01	
	12 months	0.021	0.037	5.71E-01	0.034	-0.036	3.41E-01	0.010	0.034	7.81E-01	2.85E-01	6.20E-01	
	16 months	0.030	0.042	4.71E-01	0.050	-0.041	2.22E-01	-0.015	0.039	7.05E-01	1.71E-01	2.51E-01	
	2 years	0.036	0.042	3.83E-01	0.035	-0.041	3.92E-01	0.046	0.039	2.35E-01	2.21E-01	8.55E-01	
	3 years	0.008	0.041	8.49E-01	0.055	-0.041	1.78E-01	0.019	0.038	6.22E-01	2.80E-01	5.18E-01	
	5 years	-0.004	0.045	9.32E-01	0.020	-0.044	6.47E-01	0.012	0.041	7.79E-01	7.94E-01	8.87E-01	
	7 years	0.066	0.045	1.45E-01	0.082	-0.045	6.65E-02	0.005	0.042	8.96E-01	1.99E-02	2.10E-01	
	8 years	-0.007	0.051	8.88E-01	0.074	-0.050	1.40E-01	0.011	0.047	8.13E-01	3.53E-01	3.61E-01	
rs4731690	6 weeks	0.008	0.015	5.82E-01	0.025	0.015	8.35E-02	0.016	0.014	2.52E-01	4.04E-01	6.49E-01	HDL cholesterol, Triglycerides, SHBG
	3 months	-0.001	0.012	9.14E-01	0.034	0.012	6.90E-03	0.019	0.012	1.19E-01	4.70E-02	3.88E-01	
	6 months	0.006	0.012	6.12E-01	0.039	0.012	1.72E-03	0.007	0.012	5.86E-01	6.33E-02	6.04E-02	
	8 months	0.008	0.013	5.41E-01	0.031	0.013	2.10E-02	0.004	0.013	7.39E-01	2.30E-01	1.53E-01	
	12 months	0.004	0.013	7.40E-01	0.037	0.013	6.00E-03	-0.002	0.013	8.59E-01	8.76E-02	3.56E-02	
	16 months	0.017	0.015	2.58E-01	0.010	0.015	4.99E-01	-0.002	0.015	9.18E-01	7.48E-01	5.76E-01	
	2 years	0.036	0.015	1.87E-02	0.005	0.015	7.39E-01	-0.007	0.015	6.41E-01	1.53E-01	5.73E-01	
	3 years	0.021	0.015	1.57E-01	-0.007	0.015	6.18E-01	-0.019	0.014	1.85E-01	1.76E-01	5.75E-01	
	5 years	0.041	0.016	1.23E-02	-0.012	0.016	4.69E-01	-0.002	0.016	8.89E-01	2.25E-02	6.71E-01	
	7 years	-0.003	0.016	8.60E-01	0.015	0.016	3.54E-01	-0.020	0.016	2.07E-01	4.35E-01	1.23E-01	
	8 years	0.019	0.019	3.17E-01	0.007	0.019	7.13E-01	-0.042	0.018	1.86E-02	6.55E-01	5.77E-02	
rs6026426	6 weeks	-0.069	-0.028	1.20E-02	0.021	0.027	4.39E-01	0.006	0.026	8.33E-01	2.06E-01	4.84E-01	Trunk fat percentage, Arm, Leg and Body fat percentages
	3 months	-0.059	-0.023	1.06E-02	-0.006	0.023	7.95E-01	-0.012	0.022	5.90E-01	4.49E-02	5.74E-01	
	6 months	-0.063	-0.023	6.68E-03	-0.005	0.023	8.37E-01	-0.026	0.022	2.40E-01	3.76E-02	3.33E-01	
	8 months	-0.055	-0.025	2.57E-02	-0.008	0.024	7.39E-01	-0.034	0.024	1.54E-01	6.79E-02	2.18E-01	
	12 months	-0.058	-0.025	1.90E-02	-0.024	0.024	3.32E-01	-0.026	0.023	2.76E-01	1.85E-02	1.46E-01	
	16 months	-0.035	-0.028	2.21E-01	-0.034	0.027	2.08E-01	-0.028	0.027	3.03E-01	7.92E-02	1.05E-01	
	2 years	-0.043	-0.028	1.29E-01	-0.020	0.027	4.73E-01	-0.002	0.027	9.50E-01	1.12E-01	5.77E-01	
	3 years	-0.005	-0.028	8.60E-01	-0.036	0.027	1.76E-01	-0.002	0.026	9.37E-01	2.86E-01	3.05E-01	
	5 years	-0.065	-0.031	3.29E-02	-0.030	0.029	3.05E-01	-0.041	0.029	1.57E-01	2.44E-02	8.46E-02	
	7 years	-0.063	-0.031	3.87E-02	-0.013	0.030	6.61E-01	-0.013	0.029	6.47E-01	7.40E-02	5.26E-01	
	8 years	-0.033	-0.035	3.39E-01	-0.006	0.033	8.50E-01	-0.038	0.033	2.43E-01	4.11E-01	3.42E-01	
rs77708343	6 weeks	-0.004	0.031	8.97E-01	0.057	0.031	6.87E-02	0.017	0.030	5.61E-01	1.68E-01	3.62E-01	Standing height, Basal metabolic rate, Leg fat-free mass
	3 months	-0.055	0.027	3.88E-02	0.021	0.027	4.24E-01	0.023	0.026	3.58E-01	4.29E-02	9.54E-01	
	6 months	-0.057	0.027	3.18E-02	0.033	0.027	2.13E-01	0.025	0.026	3.21E-01	1.66E-02	8.29E-01	
	8 months	-0.077	0.029	7.56E-03	0.040	0.029	1.62E-01	0.043	0.027	1.11E-01	4.03E-03	9.40E-01	
	12 months	-0.039	0.029	1.70E-01	0.050	0.029	8.59E-02	0.043	0.027	1.13E-01	2.88E-02	8.71E-01	
	16 months	-0.043	0.032	1.88E-01	0.015	0.033	6.52E-01	0.037	0.031	2.24E-01	2.14E-01	6.23E-01	
	2 years	-0.028	0.033	3.88E-01	0.044	0.033	1.82E-01	0.003	0.030	9.18E-01	1.20E-01	3.62E-01	
	3 years	-0.014	0.032	6.67E-01	0.044	0.033	1.82E-01	0.011	0.030	7.04E-01	2.09E-01	4.65E-01	
	5 years	-0.049	0.035	1.57E-01	0.061	0.036	8.70E-02	0.038	0.033	2.48E-01	2.69E-02	6.41E-01	
	7 years	-0.038	0.035	2.82E-01	0.080	0.036	2.50E-02	0.089	0.034	8.60E-03	1.87E-02	8.63E-01	
	8 years	-0.023	0.041	5.68E-01	0.047	0.040	2.45E-01	0.060	0.038	1.11E-01	2.22E-01	8.06E-01	
rs3847104	6 weeks	0.012	0.023	5.94E-01	-0.031	0.023	1.78E-01	0.009	0.022	6.97E-01	1.85E-01	2.16E-01	Hip circumference
	3 months	-0.013	0.019	4.88E-01	-0.036	0.019	6.25E-02	-0.017	0.019	3.70E-01	4.06E-01	4.74E-01	
	6 months	0.003	0.019	8.71E-01	-0.031	0.019	1.09E-01	-0.005	0.019	7.74E-01	2.10E-01	3.40E-01	
	8 months	-0.018	0.021	3.75E-01	-0.020	0.020	3.34E-01	0.015	0.020	4.43E-01	9.57E-01	2.20E-01	
	12 months	0.004	0.021	8.42E-01	-0.010	0.021	6.33E-01	0.004	0.020	8.34E-01	6.32E-01	6.25E-01	
	16 months	0.004	0.023	8.78E-01	0.006	0.023	8.09E-01	0.024	0.023	2.93E-01	9.50E-01	5.76E-01	
	2 years	-0.001	0.023	9.63E-01	-0.037	0.023	1.16E-01	0.024	0.023	2.89E-01	2.81E-01	6.21E-02	
	3 years	0.016	0.023	4.91E-01	-0.016	0.023	4.92E-01	0.017	0.022	4.58E-01	3.31E-01	3.12E-01	
	5 years	0.016	0.025	5.32E-01	0.005	0.025	8.49E-01	0.032	0.025	1.88E-01	7.57E-01	4.33E-01	
	7 years	-0.024	0.025	3.34E-01	-0.018	0.025	4.70E-01	0.034	0.025	1.68E-01	8.61E-01	1.39E-01	
	8 years	0.029	0.029	3.03E-01	-0.025	0.028	3.89E-01	0.008	0.028	7.69E-01	1.81E-01	4.10E-01	

Supplementary Table 9 | Replication of associations with infancy and childhood BMI in the MoBa cohort. SNP ID: variant rs id; BETA, SE and P denote effect sizes, standard errors and P-values; PAT, MAT and MAT NT denote paternal transmitted, maternal transmitted, and maternal untransmitted alleles association coefficients; P DIFF: Z-score P-value for the differential effect between paternal and maternal transmitted alleles; P DIFF MAT: Z-score P-value for the differential effect between maternal transmitted and maternal untransmitted alleles. Bold P DIFF denote significant POEs.

✉ Corresponding authors: Robin J. Hofmeister and Zoltán Kutalik

ID	BMI timepoints	BETA PAT	SE PAT	P PAT	BETA MAT	SE MAT	P MAT	BETA MAT NT	SE MAT NT	P MAT NT	P DIFF	P DIFF MAT	INITIAL TRAITS WITH POE AT THIS LOCUS
rs576603	6 weeks	-0.020	0.015	1.87E-01	0.006	0.015	6.76E-01	0.009	0.014	5.43E-01	2.19E-01	9.03E-01	
	3 months	-0.012	0.013	3.47E-01	0.021	0.013	9.49E-02	0.005	0.012	6.85E-01	6.49E-02	3.58E-01	
	6 months	-0.035	0.013	5.35E-03	0.004	0.013	7.61E-01	0.008	0.012	5.25E-01	2.87E-02	8.23E-01	
	8 months	-0.013	0.014	3.49E-01	0.010	0.014	4.57E-01	0.010	0.013	4.65E-01	2.35E-01	9.79E-01	
	12 months	-0.019	0.014	1.71E-01	0.007	0.014	6.02E-01	-0.001	0.013	9.56E-01	1.81E-01	6.80E-01	
	16 months	-0.021	0.015	1.72E-01	0.007	0.015	6.56E-01	-0.003	0.015	8.20E-01	2.00E-01	6.32E-01	
	2 years	-0.006	0.015	6.78E-01	0.011	0.015	4.75E-01	-0.001	0.015	9.26E-01	4.24E-01	5.63E-01	
	3 years	-0.015	0.015	3.27E-01	0.015	0.015	3.25E-01	-0.006	0.015	6.80E-01	1.65E-01	3.20E-01	
	5 years	-0.001	0.017	9.62E-01	0.017	0.017	3.11E-01	-0.016	0.016	3.32E-01	4.54E-01	1.61E-01	
	7 years	-0.010	0.017	5.35E-01	0.006	0.017	7.35E-01	0.011	0.016	4.88E-01	4.98E-01	8.11E-01	
	8 years	0.004	0.019	8.34E-01	0.027	0.019	1.44E-01	0.002	0.018	9.22E-01	3.78E-01	3.25E-01	
rs77708343	6 weeks	-0.102	0.031	1.00E-03	0.143	0.031	3.70E-06	0.021	0.030	4.85E-01	**2.15E-08**	1.31E-04	
	3 months	-0.121	0.027	4.79E-06	0.068	0.027	1.04E-02	0.027	0.025	2.82E-01	**4.56E-07**	9.44E-03	
	6 months	-0.120	0.027	5.95E-06	0.077	0.027	4.09E-03	0.016	0.025	5.38E-01	**1.71E-07**	1.23E-02	
	8 months	-0.141	0.029	9.09E-07	0.071	0.029	1.42E-02	0.008	0.027	7.81E-01	**1.99E-07**	4.81E-02	
	12 months	-0.175	0.029	9.68E-10	0.094	0.029	1.16E-03	0.008	0.027	7.68E-01	**3.70E-11**	1.02E-02	
	16 months	-0.169	0.032	1.73E-07	0.112	0.033	6.62E-04	-0.013	0.030	6.68E-01	**1.10E-09**	2.71E-02	Standing height
	2 years	-0.156	0.032	1.68E-06	0.116	0.033	4.19E-04	-0.051	0.030	9.59E-02	**4.21E-09**	1.44E-01	
	3 years	-0.143	0.032	8.01E-06	0.103	0.033	1.74E-03	-0.034	0.030	2.60E-01	**8.21E-08**	1.21E-01	
	5 years	-0.117	0.034	6.61E-04	0.103	0.035	3.41E-03	-0.021	0.033	5.20E-01	**7.65E-06**	8.91E-02	
	7 years	-0.091	0.035	9.15E-03	0.079	0.036	2.75E-02	-0.065	0.034	5.22E-02	**6.76E-04**	7.85E-01	
	8 years	-0.116	0.040	3.82E-03	0.097	0.039	1.33E-02	-0.050	0.037	1.77E-01	**1.45E-04**	3.76E-01	
rs67004488 *(proxy rs143840904)*	6 weeks	0.039	0.044	3.72E-01	-0.113	0.045	1.15E-02	-0.012	0.042	7.69E-01	1.50E-02	1.01E-01	
	3 months	-0.020	0.038	6.03E-01	-0.096	0.039	1.34E-02	0.011	0.036	7.50E-01	1.60E-01	4.21E-02	
	6 months	0.002	0.038	9.68E-01	-0.118	0.039	2.31E-03	-0.003	0.036	9.38E-01	2.72E-02	2.92E-02	
	8 months	-0.036	0.041	3.74E-01	-0.079	0.041	5.66E-02	-0.006	0.038	8.69E-01	4.61E-01	1.99E-01	
	12 months	-0.043	0.041	2.85E-01	-0.076	0.041	6.59E-02	-0.010	0.038	7.99E-01	5.74E-01	2.41E-01	
	16 months	-0.051	0.047	2.70E-01	-0.082	0.047	7.92E-02	-0.001	0.044	9.87E-01	6.47E-01	2.04E-01	
	2 years	-0.051	0.046	2.72E-01	-0.089	0.047	5.60E-02	-0.021	0.043	6.31E-01	5.63E-01	2.84E-01	
	3 years	-0.055	0.046	2.35E-01	-0.134	0.046	3.62E-03	-0.043	0.043	3.10E-01	2.21E-01	1.49E-01	
	5 years	-0.031	0.050	5.36E-01	-0.099	0.050	4.71E-02	-0.043	0.047	3.62E-01	3.33E-01	4.15E-01	
	7 years	0.024	0.051	6.30E-01	-0.092	0.052	7.59E-02	-0.034	0.047	4.70E-01	1.08E-01	4.08E-01	
	8 years	0.029	0.058	6.13E-01	-0.079	0.055	1.52E-01	-0.027	0.054	6.19E-01	1.75E-01	4.99E-01	

Supplementary Table 10 | Replication of associations with infancy and childhood height in the MoBa cohort. SNP ID: variant rs id; BETA, SE and P denote effect sizes, standard errors and P-values; PAT, MAT and MAT NT denote paternal transmitted, maternal transmitted, and maternal untransmitted alleles association coefficients; P DIFF: Z-score P-value for the differential effect between paternal and maternal transmitted alleles; P DIFF MAT: Z-score P-value for the differential effect between maternal transmitted and maternal untransmitted alleles. Bold P DIFF denote significant POEs.

✉ Corresponding authors: Robin J. Hofmeister and Zoltán Kutalik

CHR	POS	SNP ID	A0	A1	P DIFF COMB	P DIFF MALES	P DIFF FEMALES	Pz	TRAIT
11	1914139	rs576603	C	T	3.86E-08	3.53E-04	1.77E-04	7.34E-01	Standing height
11	2040272	rs77708343	A	G	2.18E-09	2.61E-03	2.88E-08	4.51E-02	Standing height
11	2813322	rs143840904	C	T	5.21E-13	4.48E-06	1.15E-08	2.53E-01	Standing height
11	2040272	rs77708343	A	G	7.53E-08	1.43E-05	1.24E-03	6.68E-01	Basal metabolic rate
11	2040272	rs77708343	A	G	1.80E-08	1.05E-05	4.44E-04	8.22E-01	Leg fat-free mass
11	2040272	rs77708343	A	G	1.63E-07	1.11E-05	2.99E-03	4.94E-01	Whole body water mass
11	2041348	rs78507815	G	T	3.61E-07	4.57E-06	9.55E-03	2.71E-01	Trunk fat-free mass
**11**	**1703564**	**rs4417225**	**C**	**T**	**7.40E-08**	**4.79E-09**	**1.93E-01**	**1.38E-03**	**Glucose**
11	1702929	rs10838787	G	A	1.87E-09	7.56E-06	4.47E-05	4.49E-01	Type 2 diabetes
11	1702929	rs10838787	G	A	2.77E-17	2.29E-10	8.53E-08	4.03E-01	Glycated haemoglobin
11	2858295	rs2299620	C	T	2.23E-06	1.52E-04	4.23E-03	4.48E-01	Glycated haemoglobin
11	1920285	rs4264135	A	G	2.22E-07	1.42E-04	1.72E-04	8.67E-01	Urate
11	1998031	rs170102	G	A	5.98E-14	2.98E-09	1.69E-06	8.34E-01	Cystatin C
11	2003944	rs217215	T	G	4.94E-11	4.15E-07	2.00E-05	9.43E-01	Creatinine
7	130009312	rs10239342	T	G	1.87E-06	2.42E-06	9.73E-03	5.34E-01	SHBG
7	130016470	rs62471721	G	A	6.51E-09	2.03E-07	1.52E-03	1.54E-01	Triglycerides
7	130017940	rs4731690	A	G	1.92E-06	2.62E-04	1.89E-03	8.51E-01	HDL cholesterol
7	130400698	rs3847104	G	A	3.81E-07	3.56E-03	4.89E-04	2.55E-01	Hip circumference
7	130463192	rs6467315	C	G	2.20E-06	5.05E-02	5.03E-06	9.52E-03	Hip circumference
20	57216538	rs80116540	A	G	3.39E-08	4.70E-03	1.36E-06	3.42E-02	Arm fat percentage
20	57216538	rs80116540	A	G	2.06E-07	5.35E-03	7.47E-06	9.69E-02	Body fat percentage
20	57226079	rs6026426	C	G	1.29E-06	5.63E-03	6.97E-05	1.51E-01	Trunk fat percentage
20	57484934	rs3730173	C	T	5.32E-07	9.03E-04	1.59E-04	5.21E-01	Leg fat percentage
6	144274210	rs12528876	T	C	2.96E-06	2.68E-04	2.62E-03	8.62E-01	IGF-1
16	3412861	rs7188903	A	G	6.14E-07	6.83E-05	2.00E-03	7.25E-01	IGF-1
15	25983333	rs146982369	C	G	2.59E-06	1.12E-03	6.15E-04	7.00E-01	Total protein
14	101185187	rs59228823	G	C	1.92E-14	5.83E-08	5.43E-08	7.05E-01	Platelet count
3	169482335	rs2293607	T	C	5.70E-07	3.50E-04	4.22E-04	8.44E-01	TS ratio
4	164012901	rs11100479	C	T	6.25E-07	3.00E-04	5.41E-04	9.20E-01	TS ratio
5	1285974	rs7705526	C	A	5.82E-11	5.87E-05	1.33E-07	2.48E-01	TS ratio

Supplementary Table 11 | Sex-specificity of parent-of-origin effects.

CHR: chromosome; POS: genetic position (hg19); SNP ID: variant rs id; A0: reference allele; A1: assessed allele; P DIFF denote P-values for the differential test; COMB, MALES, and FEMALES denote combined sex, males only and females only. P_Z: denotes the P-values for the comparison tests between males-specific and females-specific coefficients using a Z-score approach; TRAIT: phenotype name. Bold indicates the significant sex-specific POE.

CHR	POS (hg38)	UKB SNP	ASSESSED SNP	LD	A0	A1	N	BETA PAT	SE PAT	P PAT	BETA MAT	SE MAT	P MAT	BETA DIFF	SE DIFF	P DIFF	TRAIT
**14**	**100718850**	**rs59228823**	**rs59228823**		**G**	**C**	**67266**	**-0.007**	**0.008**	**3.55E-01**	**-0.087**	**0.008**	**4.49E-30**	**0.078**	**0.011**	**2.97E-13**	**Platelet count**
**11**	**1899055**	**rs217215**	**rs4264135**	**0.613**	**A**	**G**	**77270**	**-0.028**	**0.006**	**2.21E-06**	**0.030**	**0.006**	**4.27E-07**	**-0.058**	**0.008**	**4.40E-12**	**Creatinine**
**11**	**2792092**	**rs143840904**	**rs143840904**		**C**	**T**	**61630**	**-0.005**	**0.020**	**7.98E-01**	**-0.175**	**0.021**	**1.70E-17**	**0.162**	**0.028**	**7.94E-09**	**Standing height**
**7**	**130378099**	**rs4731690**	**rs4731690**		**A**	**G**	**81884**	**0.009**	**0.002**	**1.18E-05**	**-0.007**	**0.002**	**1.01E-03**	**0.016**	**0.003**	**6.86E-08**	**HDL cholesterol**
**11**	**1921890**	**rs576603**	**rs735955**	**0.881**	**G**	**A**	**61630**	**-0.027**	**0.005**	**1.39E-07**	**0.005**	**0.005**	**3.11E-01**	**-0.033**	**0.007**	**6.40E-06**	**Standing height**
**11**	**1694102**	**rs10838787**	**rs7130416**	**0.921**	**T**	**C**	**20203**	**0.054**	**0.013**	**2.17E-05**	**-0.027**	**0.013**	**3.46E-02**	**0.079**	**0.018**	**9.51E-06**	**Glycated haemoglobin**
**7**	**130376629**	**rs62471721**	**rs62471721**		**G**	**A**	**81884**	**-0.012**	**0.006**	**5.21E-02**	**0.024**	**0.006**	**4.74E-05**	**-0.036**	**0.008**	**1.91E-05**	**Triglycerides**
**11**	**1682334**	**rs10838787**	**rs4417225**	**0.998**	**C**	**T**	**85050**	**0.092**	**0.035**	**9.60E-03**	**-0.101**	**0.035**	**4.17E-03**	**0.189**	**0.050**	**1.47E-04**	**Type 2 diabetes**
**11**	**1682334**	**rs4417225**	**rs4417225**		**C**	**T**	**80403**	**0.030**	**0.009**	**5.87E-04**	**-0.017**	**0.009**	**5.81E-02**	**0.046**	**0.012**	**1.92E-04**	**Glucose**
**11**	**1904723**	**rs170102**	**rs61868802**	**0.608**	**A**	**G**	**21851**	**0.029**	**0.011**	**6.33E-03**	**-0.015**	**0.011**	**1.66E-01**	**0.045**	**0.015**	**3.15E-03**	**Cystatin C**
11	2837065	rs2299620	rs2299620		C	T	20203	0.060	0.034	8.07E-02	-0.070	0.034	3.83E-02	0.130	0.048	6.79E-03	Glycated haemoglobin
11	1902116	rs77708343	rs145861779	0.479	C	T	61630	-0.044	0.012	2.12E-04	-0.006	0.012	6.36E-01	-0.040	0.016	1.56E-02	Standing height
7	130715870	rs3847104	rs3847104		G	A	55874	-0.010	0.010	3.29E-01	0.010	0.010	3.55E-01	-0.020	0.015	1.70E-01	Hip circumference
7	130778999	rs6467315	rs10954284	0.999	T	A	55874	0.001	0.007	8.78E-01	0.009	0.007	1.81E-01	-0.008	0.010	4.07E-01	Hip circumference

Supplementary Table 12 | Replication of parent-of-origin effects in the Estonian Biobank cohort.
CHR: chromosome; POS: genetic position (hg38); UKBB SNP: variant exhibiting POE on the trait in the UK Biobank (UKBB); ASSESSED SNP: variant tested for POE in the Estonian Biobank (EstBB); LD: Linkage Disequilibrium (LD) between the UKBB variant and the assessed variant, if not the same variant. LD was computed in the UK Biobank cohort; A0: reference allele of the assessed variant in the EstBB; A1: assessed allele of the assessed variant in the EstBB; N: number of individuals with available trait levels (includes individuals both homozygous and heterozygous for the assessed variant); BETA, SE and P denote effect sizes, standard errors and P-values; PAT, MAT and DIFF denote paternal, maternal and differential tests. TRAIT: phenotype name. Bold indicate replicated associations.

CHR	POS	SNP ID	A0	A1	TRAIT	Assessed	Replicated
11	1914139	rs576603	C	T	Standing height* (c)	Moba-height/EstBB	EstBB
11	2813322	rs143840904	C	T	Standing height*	Moba-height/EstBB	EstBB
11	2040272	rs77708343	A	G	Standing height*	Moba-height/EstBB	Moba
11	2040272	rs77708343	A	G	Basal metabolic rate	Moba-bmi	
11	2040272	rs77708343	A	G	Leg fat-free mass*		
11	2040272	rs77708343	A	G	Whole body water mass		
11	2041348	rs78507815	G	T	Trunk fat-free mass		
11	1920285	rs4264135	A	G	Urate		
11	1998031	rs170102	G	A	Cystatin C*	EstBB	EstBB
11	2003944	rs217215	T	G	Creatinine*	EstBB	EstBB
11	1703564	rs4417225	C	T	Glucose	EstBB	EstBB
11	1702929	rs10838787	G	A	Type 2 diabetes*	Moba-bmi/EstBB	EstBB
11	1702929	rs10838787	G	A	Glycated haemoglobin*	Moba-bmi/EstBB	EstBB
11	2858295	rs2299620	C	T	Glycated haemoglobin	Moba-bmi/EstBB	
7	130009312	rs10239342	T	G	SHBG		
7	130016470	rs62471721	G	A	Triglycerides*	EstBB	EstBB
7	130017940	rs4731690	A	G	HDL cholesterol	Moba-bmi/EstBB	EstBB
7	130463192	rs6467315	C	G	Hip circumference	Moba-bmi/EstBB	Moba
7	130400698	rs3847104	G	A	Hip circumference (c)	Moba-bmi/EstBB	EstBB
20	57216538	rs80116540	A	G	Arm fat percentage*		
20	57216538	rs80116540	A	G	Body fat percentage		
20	57226079	rs6026426	C	G	Trunk fat percentage	Moba-bmi/EstBB	
20	57484934	rs3730173	C	T	Leg fat percentage		
6	144274210	rs12528876	T	C	IGF-1		
16	3412861	rs7188903	A	G	IGF-1		
15	25983333	rs146982369	C	G	Total protein		
14	101185187	rs59228823	G	C	Platelet count*	EstBB	EstBB
3	169482335	rs2293607	T	C	TS ratio		
4	164012901	rs11100479	C	T	TS ratio		
5	1285974	rs7705526	C	A	TS ratio		

Supplementary Table 13 | Summary of POEs replication.

CHR: chromosome; POS: genetic position (hg19); SNP ID: variant rs id; A0: reference allele; A1: assessed allele; A1FREQ: A1 allele frequency; TRAIT: phenotype name; Assessed: tested for replication, specify in which cohort ; Replicated: specify in which cohort it was replicated. Highlighted colors denote clusters of POEs associated with traits sharing substantial similarities, as in Table 1.

References

[1] Robin J. Hofmeister et al. "Accurate rare variant phasing of whole-genome and whole-exome sequencing data in the UK Biobank". In: *Nature Genetics* 55.7 (July 2023). Publisher: Nature Publishing Group, pp. 1243–1249. ISSN: 1546-1718. DOI: 10.1038/s41588-023-01415-w. URL: https://www.nature.com/articles/s41588-023-01415-w (visited on 08/08/2024).

[2] Robin J. Hofmeister et al. "Parent-of-Origin inference for biobanks". In: *Nature Communications* 13.1 (Nov. 5, 2022). Publisher: Nature Publishing Group, p. 6668. ISSN: 2041-1723. DOI: 10.1038/s41467-022-34383-6. URL: https://www.nature.com/articles/s41467-022-34383-6 (visited on 08/07/2024).

[3] Thorhildur Juliusdottir et al. "Distinction between the effects of parental and fetal genomes on fetal growth". In: *Nature Genetics* 53.8 (Aug. 2021), pp. 1135–1142. ISSN: 1546-1718. DOI: 10.1038/s41588-021-00896-x.

[4] THE GTEX CONSORTIUM. "The GTEx Consortium atlas of genetic regulatory effects across human tissues". In: *Science* 369.6509 (Sept. 11, 2020). Publisher: American Association for the Advancement of Science, pp. 1318–1330. DOI: 10.1126/science.aaz1776. URL: https://www.science.org/doi/10.1126/science.aaz1776 (visited on 10/25/2024).

[5] Hye In Kim et al. "Genome-wide survey of parent-of-origin-specific associations across clinical traits derived from electronic health records". In: *Human Genetics and Genomics Advances* 2.3 (June 11, 2021), p. 100039. ISSN: 2666-2477. DOI: 10.1016/j.xhgg.2021.100039. URL: https://www.ncbi.nlm.nih.gov/pmc/articles/PMC8756508/ (visited on 08/08/2024).

[6] Augustine Kong et al. "Parental origin of sequence variants associated with complex diseases". In: *Nature* 462.7275 (Dec. 2009). Publisher: Nature Publishing Group, pp. 868–874. ISSN: 1476-4687. DOI: 10.1038/nature08625. URL: https://www.nature.com/articles/nature08625 (visited on 08/07/2024).

[7] Stefania Benonisdottir et al. "Epigenetic and genetic components of height regulation". In: *Nature Communications* 7.1 (Nov. 16, 2016). Publisher: Nature Publishing Group, p. 13490. ISSN: 2041-1723. DOI: 10.1038/ncomms13490. URL: https://www.nature.com/articles/ncomms13490 (visited on 10/25/2024).

[8] Einat Granot-Hershkovitz et al. "Searching for parent-of-origin effects on cardiometabolic traits in imprinted genomic regions". In: *European Journal of Human Genetics* 28.5 (May 2020). Publisher: Nature Publishing Group, pp. 646–655. ISSN: 1476-5438. DOI: 10.1038/s41431-019-0568-1. URL: https://www.nature.com/articles/s41431-019-0568-1 (visited on 10/25/2024).

[9] Magdalena Zoledziewska et al. "Height-reducing variants and selection for short stature in Sardinia". In: *Nature Genetics* 47.11 (Nov. 2015). Publisher: Nature Publishing Group, pp. 1352–1356. ISSN: 1546-1718. DOI: 10.1038/ng.3403. URL: https://www.nature.com/articles/ng.3403 (visited on 10/25/2024).

✉ Corresponding authors: Robin J. Hofmeister and Zoltán Kutalik

[10] Xiaoying Wang et al. "Diabetes knowledge predicts HbA1c levels of people with type 2 diabetes mellitus in rural China: a ten-month follow-up study". In: *Scientific Reports* 13.1 (Oct. 25, 2023). Publisher: Nature Publishing Group, p. 18248. ISSN: 2045-2322. DOI: 10.1038/s41598-023-45312-y. URL: https://www.nature.com/articles/s41598-023-45312-y (visited on 11/18/2024).

[11] Urmo Võsa et al. "Large-scale cis- and trans-eQTL analyses identify thousands of genetic loci and polygenic scores that regulate blood gene expression". In: *Nature Genetics* 53.9 (Sept. 2021). Publisher: Nature Publishing Group, pp. 1300–1310. ISSN: 1546-1718. DOI: 10.1038/s41588-021-00913-z. URL: https://www.nature.com/articles/s41588-021-00913-z (visited on 10/25/2024).

[12] Yongtao Guan and Daniel Levy. *Abundant Parent-of-origin Effect eQTL in Humans: The Framingham Heart Study.* Pages: 2024.06.05.597677 Section: New Results. June 10, 2024. DOI: 10.1101/2024.06.05.597677. URL: https://www.biorxiv.org/content/10.1101/2024.06.05.597677v1 (visited on 11/15/2024).

[13] Eleonora Porcu et al. "Limited evidence for blood eQTLs in human sexual dimorphism". In: *Genome Medicine* 14.1 (Aug. 11, 2022), p. 89. ISSN: 1756-994X. DOI: 10.1186/s13073-022-01088-w. URL: https://doi.org/10.1186/s13073-022-01088-w (visited on 11/01/2024).

[14] Elena Bernabeu et al. "Sex differences in genetic architecture in the UK Biobank". In: *Nature Genetics* 53.9 (Sept. 2021). Publisher: Nature Publishing Group, pp. 1283–1289. ISSN: 1546-1718. DOI: 10.1038/s41588-021-00912-0. URL: https://www.nature.com/articles/s41588-021-00912-0 (visited on 10/31/2024).

[15] Per Magnus et al. "Cohort Profile Update: The Norwegian Mother and Child Cohort Study (MoBa)". In: *International Journal of Epidemiology* 45.2 (Apr. 2016), pp. 382–388. ISSN: 1464-3685. DOI: 10.1093/ije/dyw029.

[16] Øyvind Helgeland et al. "Characterization of the genetic architecture of infant and early childhood body mass index". In: *Nature Metabolism* 4.3 (Mar. 2022). Publisher: Nature Publishing Group, pp. 344–358. ISSN: 2522-5812. DOI: 10.1038/s42255-022-00549-1. URL: https://www.nature.com/articles/s42255-022-00549-1 (visited on 12/03/2024).

[17] Benjamin B. Sun et al. "Plasma proteomic associations with genetics and health in the UK Biobank". In: *Nature* 622.7982 (Oct. 2023). Publisher: Nature Publishing Group, pp. 329–338. ISSN: 1476-4687. DOI: 10.1038/s41586-023-06592-6. URL: https://www.nature.com/articles/s41586-023-06592-6 (visited on 10/25/2024).

[18] M. Kamiya et al. "The cell cycle control gene ZAC/PLAGL1 is imprinted–a strong candidate gene for transient neonatal diabetes". In: *Human Molecular Genetics* 9.3 (Feb. 12, 2000), pp. 453–460. ISSN: 0964-6906. DOI: 10.1093/hmg/9.3.453.

[19] Deborah Mackay et al. "Clinical utility gene card for: Transient Neonatal Diabetes Mellitus, 6q24-related". In: *European Journal of Human Genetics* 22.9 (Sept. 2014). Publisher: Nature Publishing Group, pp. 1153–1153. ISSN: 1476-5438. DOI: 10.1038/ejhg.2014.27. URL: https://www.nature.com/articles/ejhg201427 (visited on 10/30/2024).

✉ Corresponding authors: Robin J. Hofmeister and Zoltán Kutalik

[20] Hao Hong et al. "Central IGF1 improves glucose tolerance and insulin sensitivity in mice". In: *Nutrition & Diabetes* 7.12 (Dec. 19, 2017). Publisher: Nature Publishing Group, pp. 1–10. ISSN: 2044-4052. DOI: 10.1038/s41387-017-0002-0. URL: https://www.nature.com/articles/s41387-017-0002-0 (visited on 11/01/2024).

[21] D. S. Matassa et al. "Oxidative metabolism drives inflammation-induced platinum resistance in human ovarian cancer". In: *Cell Death & Differentiation* 23.9 (Sept. 2016). Publisher: Nature Publishing Group, pp. 1542–1554. ISSN: 1476-5403. DOI: 10.1038/cdd.2016.39. URL: https://www.nature.com/articles/cdd201639 (visited on 10/25/2024).

[22] Damian Szklarczyk et al. "The STRING database in 2023: protein-protein association networks and functional enrichment analyses for any sequenced genome of interest". In: *Nucleic Acids Research* 51 (D1 Jan. 6, 2023), pp. D638–D646. ISSN: 1362-4962. DOI: 10.1093/nar/gkac1000.

[23] Wende Zhu et al. "Meg3-DMR, not the Meg3 gene, regulates imprinting of the Dlk1-Dio3 locus". In: *Developmental Biology* 455.1 (Nov. 1, 2019), pp. 10–18. ISSN: 1095-564X. DOI: 10.1016/j.ydbio.2019.07.005.

[24] Tomohiko Kayashima et al. "The novel imprinted carboxypeptidase A4 gene (CPA4) in the 7q32 imprinting domain". In: *Human Genetics* 112.3 (Mar. 2003), pp. 220–226. ISSN: 0340-6717. DOI: 10.1007/s00439-002-0891-3.

[25] Bharati Jadhav et al. "RNA-Seq in 296 phased trios provides a high-resolution map of genomic imprinting". In: *BMC Biology* 17.1 (June 24, 2019), p. 50. ISSN: 1741-7007. DOI: 10.1186/s12915-019-0674-0. URL: https://doi.org/10.1186/s12915-019-0674-0 (visited on 10/25/2024).

[26] Florian Zink et al. "Insights into imprinting from parent-of-origin phased methylomes and transcriptomes". In: *Nature Genetics* 50.11 (Nov. 2018). Publisher: Nature Publishing Group, pp. 1542–1552. ISSN: 1546-1718. DOI: 10.1038/s41588-018-0232-7. URL: https://www.nature.com/articles/s41588-018-0232-7 (visited on 10/25/2024).

[27] Yanni Zeng et al. "Parent of origin genetic effects on methylation in humans are common and influence complex trait variation". In: *Nature Communications* 10.1 (Mar. 27, 2019). Publisher: Nature Publishing Group, p. 1383. ISSN: 2041-1723. DOI: 10.1038/s41467-019-09301-y. URL: https://www.nature.com/articles/s41467-019-09301-y (visited on 03/07/2025).

[28] Nicola Pirastu et al. "Genetic analyses identify widespread sex-differential participation bias". In: *Nature Genetics* 53.5 (May 2021). Publisher: Nature Publishing Group, pp. 663–671. ISSN: 1546-1718. DOI: 10.1038/s41588-021-00846-7. URL: https://www.nature.com/articles/s41588-021-00846-7 (visited on 11/20/2024).

[29] Heather A. Lawson, James M. Cheverud, and Jason B. Wolf. "Genomic imprinting and parent-of-origin effects on complex traits". In: *Nature reviews. Genetics* 14.9 (Sept. 2013), pp. 609–617. ISSN: 1471-0056. DOI: 10.1038/nrg3543. URL: https://www.ncbi.nlm.nih.gov/pmc/articles/PMC3926806/ (visited on 08/08/2024).

✉ Corresponding authors: Robin J. Hofmeister and Zoltán Kutalik

[30] Ani Manichaikul et al. "Robust relationship inference in genome-wide association studies". In: *Bioinformatics* 26.22 (Nov. 15, 2010), pp. 2867–2873. ISSN: 1367-4811, 1367-4803. DOI: 10.1093/bioinformatics/btq559. URL: https://academic.oup.com/bioinformatics/article/26/22/2867/228512 (visited on 08/08/2024).

[31] Joelle Mbatchou et al. "Computationally efficient whole-genome regression for quantitative and binary traits". In: *Nature Genetics* 53.7 (July 2021). Publisher: Nature Publishing Group, pp. 1097–1103. ISSN: 1546-1718. DOI: 10.1038/s41588-021-00870-7. URL: https://www.nature.com/articles/s41588-021-00870-7 (visited on 10/25/2024).

✉ Corresponding authors: Robin J. Hofmeister and Zoltán Kutalik

Figure 1 Inter-chromosomal phasing and parent-of-origin methods overview.

a) Inter-chromosomal phasing and parent-of-origin inference. We initially clustered relatives (2nd, 3rd and 4th degree) into surrogate parent groups, segregating the relatives into "one family side" (green) *vs* "the other family side" (orange). We then combined two steps to infer the PofO of a focal individual: on the one hand, we leveraged IBD sharing with surrogate parent groups to perform inter-chromosomal phasing. For instance, green IBD tracks were forced to align on the first haplotype, simultaneously correcting for intra-chromosomal phasing errors (see Supplementary Note 1); on the other hand, we examined genetic similarities on chromosome X and mtDNA between the focal individual and its surrogate parents, to assign surrogate parent groups to a parental side. Finally, we deduce the PofO of inter-chromosomally phased haplotypes from the parental side of the surrogate parent in IBD (see Methods for details). **b**) To determine the PofO of siblings, we first inferred crossover events using IBD haplotypes. We then overlapped inferred crossovers with sex-specific genetic map to derive the likelihood of a crossover originating from the mother or the father. This subsequently allowed us to deduce the PofO of the haplotype carrying the crossover.

Figure 2 Significant parent-of-origin effects.

a) Heatmap summarizing the differential P-value for all significant POEs identified in this study, computed using REGENIE[31] (two-sided test). Rows correspond to genetic variants ordered by genetic position and annotated with the genes and chromosome bands, while columns represent phenotypes. The color intensity represents the magnitude of the differential Z-scores, with darker shades indicating stronger differential effects. Cells with black rectangles indicate significant POEs identified in this SNP-trait pair and reported in Table 1. White annotations within cells specify the type of approach that led to the identification of the POE (also shown in Table 1): "I" for imprinted region-focused analysis, "A" for additively associated region-focused analysis, "G" for genome-wide analysis. The LD heatmap on the right shows linkage disequilibrium (LD; r^2) between the variants. **b**) Scatter plot illustrating the parental Z-scores for all significant POEs identified in this study and reported in Table 1. Each point represents a significant POE, colored and shaped by phenotype. The dashed red line represents the line of equality, and dashed white lines represent zero values for paternal and maternal Z-scores, respectively. Areas are filled according to the POE classification, also shown in Table 1 (see Methods for details on the classification). Labeled points correspond to POE classified as paternal or maternal effects. Unlabeled points correspond to bi-polar POEs.

Figure 3 Significant bi-polar parent-of-origin effect on triglycerides.

a) Differential GWAS shows the association strength ($-log_{10}(p - value)$, y-axis) against the genomic position (x-axis) on triglycerides. Each point represents a genetic variant. Diamond indicates the variant with independent POE found in this study and listed in Table 1. Red and orange lines represent the significance threshold used to identify significant

✉ Corresponding authors: Robin J. Hofmeister and Zoltán Kutalik

and suggestively significant POEs (see Methods). Linkage Disequilibrium (LD) pattern (middle) ranges from LD=0 (white) to LD=1 (red). Gene positions (bottom) are shown along the genomic positions (x-axis). Horizontal lines show the gene start and end positions. Vertical lines show the exons start and end positions. Gene names are shown below the corresponding gene coordinates. Imprinted genes mentioned in the main text are highlighted in red (maternally expressed) and blue (paternally expressed). **b**) Phenotypic effect (y-axis) of the lead POE-variant's allele and parent-of-origin (x-axis) on triglycerides. Dots show mean values, and error bars show 95% confidence intervals computed as $mean \pm 1.96 \times SE$. Grey dashed lines represent the mean value of individuals carrying no alternative allele (i.e., genotype 0). Dots and bars colors indicate maternal heterozygotes (red), paternal heterozygotes (blue), and homozygotes (dark and light grey). **c**) We hypothesize that bipolar POEs at this locus may arise because SNP rs62471721 is an eQTL for two imprinted genes. Specifically, the paternal copy of the A allele affects expression of the paternally expressed gene *MEST*, while the maternal copy influences expression of the maternally expressed gene *KLF14*.

Figure 4 Parent-of-origin associations on telomere length.

a) Genome-wide associations for additive (grey), paternal (blue), maternal (red), and differential (green) effects represented as association strength ($-log_{10}(p-value)$, y-axis) against the genomic position (x-axis). Each point represents a genetic variant. Yellow dots indicate additive genome-wide significant variants (those then tested for POE). Orange diamonds indicate variants exhibiting significant POE among the lead additive effect variants. Light green diamond indicate the lead POE-variant identified in the genome-wide scan. Orange lines indicate significant thresholds (1×10^{-9} for the additive effects and 2.75×10^{-5} for POEs). **b-d**) Genotypes and PofO of alleles (x-axis) effects on telomere length (y-axis). Red and blue alleles indicate maternal and paternal alleles, respectively. Dots show mean values, and error bars show 95% confidence intervals computed as $\beta \pm 1.96 \times SE$ and derived from n=109,385 individuals. Grey dashed lines represent the mean value of individuals carrying no alternative allele (i.e., genotype 0).

✉ Corresponding authors: Robin J. Hofmeister and Zoltán Kutalik

Table 1 Significant Parent-of-origin effects identified in this study.

CHR: chromosome; POS: genetic position (hg19); SNP ID: variant rs id; A0: reference allele; A1: assessed allele; A1FREQ: A1 allele frequency. BETA, SE and P denote effect sizes, standard errors and P-values, computed using REGENIE[31] (two-sided test); PAT, MAT, DIFF and ADD denote paternal, maternal, differential and additive tests. TRAIT: phenotype name; *: POE indentified using the imprinted region-focused approach that are also robust when correcting for the number of phenotype tested (see Methods); (c) denote POE identified in a conditional analysis. For these, the conditional estimates are reported. For these, reported coefficients are those from the conditional analysis; SCAN indicate the approach (I for imprinted region-focus, A for additively associated region-focused, G for genome-wide scan); POE: indicate the POE pattern (M=paternal; P=paternal; B=bi-polar; PA=paternal asymmetric; see Methods); bold ID: bi-polar POEs. Highlighted colors denote clusters of POEs associated with traits sharing substantial similarities (i.e 18 non-independent snp-trait pair); SHBG: Sex Hormone-Binding Globulin. TS ratio: Relative leucocyte telomere length.

Extended data Figure 1 Conditional analysis on hip circumference at 7q32.2.

a) Original differential GWAS on hip circumference. **b**) Genotypes and PofO of alleles (x-axis) effects of rs6467315 on hip circumference. **c**) Conditional differential GWAS on hip circumference. **d**) Genotypes and PofO of alleles (x-axis) effects of rs3847104 on hip circumference. **a**) and **c**) show the association strength ($-log_{10}(p-value)$, y-axis) against the genomic position (x-axis) at the 7q32.2 region. Each point represents a genetic variant. The diamond indicates the variant with significant POE on hip circumference found in this study in the primary GWAS scan. The square indicates the variant with significant and independent POE identified in the conditional analysis. Red and orange lines represent the significance threshold used to identify significant and suggestive POEs (see Methods). Bottom panel shows the Linkage Disequilibrium (LD) pattern, ranging from LD=0 (white) to LD=1 (red). **b**) and **d**) show the genotypes and PofO of alleles (x-axis) effects on normalized hip circumference (y-axis). Red and blue alleles indicate maternal and paternal alleles, respectively. Dots show mean values, error bars show 95% confidence intervals computed as $\beta \pm 1.96 \times SE$ and derived from n=109,385 individuals. Grey dashed lines represent the mean value of individuals carrying no alternative allele (i.e., genotype 0). Dots and bars colors indicate maternal heterozygotes (red), paternal heterozygotes (blue), and homozygotes (dark and light grey).

Extended data Figure 2 Parent-of-Origin associations on standing height.

a) The two top panels show the original and conditional differential GWAS (top) locuszoom plot shows the association strength ($-log_{10}(p-value)$, y-axis) against the genomic position (x-axis). Each point represents a genetic variant. Diamonds indicate the variants with independent, significant POE on standing height found in this study in the primary GWAS

✉ Corresponding authors: Robin J. Hofmeister and Zoltán Kutalik

scan. The square represents the variant with significant and independent POE identified in the conditional analysis.. Red and orange lines represent the significance threshold used to identify significant and suggestive POEs (see Methods). Linkage Disequilibrium (LD) pattern (middle) ranges from LD=0 (white) to LD=1 (red). Gene positions (bottom) along the 11p15.5 imprinted region (x-axis). Horizontal lines show the gene start and end positions. Vertical lines show the exons start and end positions. Gene names are shown below the corresponding gene coordinates. **b-d**) Additive (b, grey), Paternal (c, blue) and Maternal (d, red) associations on standing height shown as association strength ($-log_{10}(p-value)$, y-axis) against the genomic position (x-axis). Green diamonds indicate POEs selected in the differential scan. Orange lines represent the significance threshold used in this study when focusing on imprinted regions (3.1×10^{-06}). **e-g**) Genotypes and PofO of alleles (x-axis) effects on standing height (y-axis). Red and blue alleles indicate maternal and paternal alleles, respectively. Dots show mean values, error bars show 95% confidence intervals computed as $\beta \pm 1.96 \times SE$ and derived from n=109,385 individuals. Grey dashed lines represent the mean value of individuals carrying no alternative allele (i.e., genotype 0). Dots and bars colors indicate maternal heterozygotes (red), paternal heterozygotes (blue), and homozygotes (dark and light grey).

Extended data Figure 3 Co-localization analyses at 11p15.5.

a) Parent-of-origin associations across traits at 11p15.5 show the differential GWAS association strength ($-log_{10}(p-value)$, y-axis) against the genomic position (x-axis). Red and orange lines represent the significance threshold used to identify significant and suggestive POEs (see Methods). Each point represents a genetic variant. Light green diamonds shows lead POE reported in Table 1. **b**) Co-localization probability heatmap (i.e, shared causal variant probability H_4).

Extended data Figure 4 Parent-of-Origin associations with type 2 diabetes.

Differential GWAS show the association strength ($-log_{10}(p-value)$, y-axis) against the genomic position (x-axis) on **a**) type 2 diabetes, **c**) HbA1c and **d**) glucose. Each point represents a genetic variant. Diamonds indicate variants with independent POE found in this study and listed in Table 1. Red and orange lines represent the significance threshold used to identify significant and suggestive POEs (see Methods). In **a**), linkage Disequilibrium (LD) pattern (middle) ranges from LD=0 (white) to LD=1 (red). Gene positions (bottom) are shown along the genomic positions (x-axis). Horizontal lines show the gene start and end positions. Vertical lines show the exons start and end positions. Gene names are shown below the corresponding gene coordinates. **b**) Genotypes and PofO of alleles (x-axis) effects on type 2 diabetes incidence (y-axis). Red and blue alleles indicate maternal and paternal alleles, respectively. Dots show mean values, error bars show 95% confidence intervals computed as $\beta \pm 1.96 \times SE$ and derived from n=108,196 individuals. Grey dashed lines represent the mean value of individuals carrying no alternative allele (i.e., genotype 0). Dots and bars colors indicate maternal heterozygotes (red), paternal heterozygotes (blue),

✉ Corresponding authors: Robin J. Hofmeister and Zoltán Kutalik

and homozygotes (dark and light grey).

Extended data Figure 5 Sex-specific POE of rs4417225 on glucose levels.

Effects of genotypes and PofO of alleles (x-axis) on glucose level (y-axis) for (**a**) both sexes combined, (**b**) females only, and (**c**) males only. Red markers represent maternal heterozygotes, blue markers represent paternal heterozygotes, and dark and light grey represent homozygotes. The data points show the mean glucose levels, with error bars indicating the 95% confidence intervals. A grey dashed line represents the mean glucose level for individuals with the reference genotype (C/C). Significance values from the differential GWAS tests are annotated within each panel.

Extended data Figure 6 Parent-of-origin effects in early life.

Dots show beta estimates and error bars show 95% confidence intervals computed as $\beta \pm 1.96 \times SE$ (y-axis) of paternal transmitted (blue), maternal transmitted (red) and maternal untransmitted (pink) alleles effects on **a**) height and **b**) BMI time points (x-axis). Beta and 95% confidence intervals estimates were derived using REGENIE[31] and using a different number of individual at each time point, as indicated in Supplementary Table 8: n=29,791; 41,865; 42,346; 36,790; 36,721; 27,595; 27,435; 27,985; 22,667; 23,280 and 17,888 for 6 weeks, 3 months, 6 months, 8 months, 1 year, 16 months, and 2 to 8 years, respectively.

Extended data Figure 7 SNPs heritability.

a Parent-of-Origin SNPs heritability h^2_{POE} was estimated within and outside imprinted regions. Stars and p-values indicate nominal significant differences between h^2_{POE} within imprinted regions *vs* outside imprinted regions. Paternal (blue) and maternal (red) h^2 was estimated (**b**) genome-wide and (**c**) within imprinted regions. Stars and p-values indicate nominal significant differences between paternal and maternal h^2. Three out of the 59 selected traits were not represented here due to disproportionate standard errors: lipoprotein A, rheumatoid factor, and oestradiol.

Extended data Figure 8 Significant Parent-of-Origin pQTLs.

Association strength ($-log_{10}(p - value)$, y-axis) against the genomic position (x-axis) for additive (grey), paternal (blue), maternal (red) and differential (green) GWAS on (**a**) DLK1, (**b**) CPA4, (**c**) ADAM23 and (**d**) PER3. Diamonds show the lead additive association previously reported[17] (grey) and the lead POE-pQTL detected in this study (green). Orange line indicate the significance threshold ($P_D < 0.05/14285 = 3.5 \times 10^{-6}$). **Bottom panel** show the effects of alleles and PofO on normalized protein levels. Dots show beta estimates and error bars show 95% confidence intervals estimates that were derived using REGENIE[31] and using n=9,168; 7,488; 9,151 and 7,614 individuals for DLK1, CPA1, ADAM23 and PER3, respectively.

✉ Corresponding authors: Robin J. Hofmeister and Zoltán Kutalik

Extended data Table 1 Significant Parent-of-Origin pQTLs.

CHR: chromosome; GENPOS: genetic position (hg19); ID: variant rs id; A0: reference allele; A1: assessed allele; A1FREQ: A1 allele frequency. BETA, SE and P denote effect sizes, standard errors and P-values, computed using REGENIE[31] (two-sided test); PAT, MAT, DIFF and ADD denote paternal, maternal, differential and additive tests. PHENAME: phenotype name.
