# Supplementary material for: Parent-of-origin effects on complex traits in up to 236,781 individuals
Source: Nature. 2025 Aug 6;646(8085):647–56. doi: 10.1038/s41586-025-09357-5 (PMC12527933; doi:10.1038/s41586-025-09357-5)
Supplement: Supplementary file 2 — Reporting Summary [file 41586_2025_9357_MOESM2_ESM.pdf]

nature portfolio

Corresponding author(s): Robin J Hofmeister
Zoltán Kutalik

Last updated by author(s): 13 June 2025

Reporting Summary

Nature Portfolio wishes to improve the reproducibility of the work that we publish. This form provides structure for consistency and transparency in reporting. For further information on Nature Portfolio policies, see our Editorial Policies and the Editorial Policy Checklist.

Statistics

For all statistical analyses, confirm that the following items are present in the figure legend, table legend, main text, or Methods section.

n/a	Confirmed	
☐	☒	The exact sample size (*n*) for each experimental group/condition, given as a discrete number and unit of measurement
☒	☐	A statement on whether measurements were taken from distinct samples or whether the same sample was measured repeatedly
☐	☒	The statistical test(s) used AND whether they are one- or two-sided<br>*Only common tests should be described solely by name; describe more complex techniques in the Methods section.*
☐	☒	A description of all covariates tested
☐	☒	A description of any assumptions or corrections, such as tests of normality and adjustment for multiple comparisons
☐	☒	A full description of the statistical parameters including central tendency (e.g. means) or other basic estimates (e.g. regression coefficient) AND variation (e.g. standard deviation) or associated estimates of uncertainty (e.g. confidence intervals)
☐	☒	For null hypothesis testing, the test statistic (e.g. *F*, *t*, *r*) with confidence intervals, effect sizes, degrees of freedom and *P* value noted<br>*Give P values as exact values whenever suitable.*
☐	☒	For Bayesian analysis, information on the choice of priors and Markov chain Monte Carlo settings
☒	☐	For hierarchical and complex designs, identification of the appropriate level for tests and full reporting of outcomes
☒	☐	Estimates of effect sizes (e.g. Cohen's *d*, Pearson's *r*), indicating how they were calculated

Our web collection on statistics for biologists contains articles on many of the points above.

Software and code

Policy information about availability of computer code

Data collection	No software was used for the data collection of this study.
Data analysis	Publicly available code used in this study :<br>THORIN v1.2 (https://github.com/RJHFMSTR/THORIN), for which we provide a full documentation and tutorials on a dedicated website (https://rjhfmstr.github.io/THORIN/).<br>BCFtools v1.8 (based on HTSlib v1.8)<br>SHAPEIT v5 (https://github.com/odelaneau/shapeit5)<br>REGENIE v3.2.9 (https://rgcgithub.github.io/regenie/)<br>PLINK v1.90b5<br>PLINK v2.00a4.3<br>R v4.3.1<br>R 'igraph' package v1.2.2<br>KING v2.2.4<br><br>Non publicly available code used in this study:<br>IMPUTE5 v1.2.1

For manuscripts utilizing custom algorithms or software that are central to the research but not yet described in published literature, software must be made available to editors and reviewers. We strongly encourage code deposition in a community repository (e.g. GitHub). See the Nature Portfolio guidelines for submitting code & software for further information.

Data

Policy information about availability of data

All manuscripts must include a data availability statement. This statement should provide the following information, where applicable:

- Accession codes, unique identifiers, or web links for publicly available datasets
- A description of any restrictions on data availability
- For clinical datasets or third party data, please ensure that the statement adheres to our policy

The summary data are publicly accessible for download on our webpage (http://poedb.dcsr.unil.ch/). The UK Biobank genetic data are available under restricted access. Access can be obtained by application via the UK Biobank Access Management System (https://www.ukbiobank.ac.uk/enable-your-research/apply-for-access). The Estonian Biobank data are also available under restricted access. The access to the Estonian Biobank data must be approved by the Scientific Advisory Committee of the Estonian Biobank and by the Estonian Committee on Bioethics and Human Research. More details are available at https://genomics.ut.ee/en/content/estonian-biobank#dataaccess. Data produced as part of this study (i.e inter-chromosomally phased data and PofO information) will be returned to their respective biobanks and access will be granted to approved researchers.
The publicly available subset of the Haplotype Reference Consortium (HRC) dataset is available from the European Genome-Phenome Archive at the European Bioinformatics Institute, accession EGAS00001001710. We used additional publicly available databases that have been consulted multiple time between September 2023 and December 2024: GeneImprint (http://www.geneimprint.com/) and the UK Biobank phenotype correlations (https://ukbb-rg.hail.is/).
- UK Biobank phenotype correlations (https://ukbb-rg.hail.is/)

Research involving human participants, their data, or biological material

Policy information about studies with human participants or human data. See also policy information about sex, gender (identity/presentation), and sexual orientation and race, ethnicity and racism.

Reporting on sex and gender	We use only genetically determined sex, that we refer to as "sex".
Reporting on race, ethnicity, or other socially relevant groupings	For the UK Biobank cohort, we used only individuals who self-identified as “White British” and have a similar genetic ancestry determined by principal components analysis (UK Biobank field 22006).<br>For the Estonian Biobank, we used only individuals determined at "Estonians" by principal components analysis.<br>For the MoBa cohort, outliers individuals, as defenied by principal components analysis, were removed.
Population characteristics	The UK Biobank population includes individuals between 37 and 73 years old at recruitment.<br>The Estonian biobank cohort includes individuals born between 1905 and 2005.<br>The MoBa cohort includes longitudinal measurements for children between 0 and 8 year of age.
Recruitment	Main analyses were performed in the UKBB, a volunteer-based cohort of 500,000 individuals from the general UK population for which participants signed a broad informed consent form.<br>MoBa is an open-ended cohort study that recruited pregnant women in Norway from 1999 to 2008.
Ethics oversight	The UK Biobank data was accessed under the project 66995 and 16389.<br>The Estonian Biobank data was accessed under the under ethical approval 1.1-12/295.<br>The use of the MoBa cohort was approved by The Regional Committee for Medical Research Ethics (#2012/67).

Note that full information on the approval of the study protocol must also be provided in the manuscript.

Field-specific reporting

Please select the one below that is the best fit for your research. If you are not sure, read the appropriate sections before making your selection.

☒ Life sciences ☐ Behavioural & social sciences ☐ Ecological, evolutionary & environmental sciences

For a reference copy of the document with all sections, see nature.com/documents/nr-reporting-summary-flat.pdf

Life sciences study design

All studies must disclose on these points even when the disclosure is negative.

Sample size	The sample size of our study for the different cohorts and phenotypes are available in supplementary table 1 (for UK Biobank cohort), supplementary table 8 (MoBa cohort) and supplementary table 11 (Estonian Biobank cohort). Sample sizes for each cohort were determined as the number of individuals with both parent-of-origin information available and phenotype measurement available.
Data exclusions	As mentioned in the Methods section, we initially filtered out all variants that were not included for the phasing of the original UK Biobank release. These were determined from the SNPs QC file provided as part of the UK Biobank, ressource 1955. For the Estonian Biobank and Moba, we used pre-filtered data provided by the data management team of each respective cohort.
Replication	We attempted to replicate a total of 16 associations (53%) discovered in this study for which we had the corresponding genetic and phenotypic data available in an additional cohort (Estonian Biobank and Moba). For reproducibility, most of the softwares used for this study are publicly available and we provided details tutorial of our inference pipeline.

Randomization	Randomization was not used since there are no experimental groups.
Blinding	Blinding is not relevant to this study since no group allocation occurs.

Reporting for specific materials, systems and methods

We require information from authors about some types of materials, experimental systems and methods used in many studies. Here, indicate whether each material, system or method listed is relevant to your study. If you are not sure if a list item applies to your research, read the appropriate section before selecting a response.

Materials & experimental systems

n/a	Involved in the study
☒	☐ Antibodies
☒	☐ Eukaryotic cell lines
☒	☐ Palaeontology and archaeology
☒	☐ Animals and other organisms
☒	☐ Clinical data
☒	☐ Dual use research of concern
☒	☐ Plants

Methods

n/a	Involved in the study
☒	☐ ChIP-seq
☒	☐ Flow cytometry
☒	☐ MRI-based neuroimaging

Plants

Seed stocks	*Report on the source of all seed stocks or other plant material used. If applicable, state the seed stock centre and catalogue number. If plant specimens were collected from the field, describe the collection location, date and sampling procedures.*
Novel plant genotypes	*Describe the methods by which all novel plant genotypes were produced. This includes those generated by transgenic approaches, gene editing, chemical/radiation-based mutagenesis and hybridization. For transgenic lines, describe the transformation method, the number of independent lines analyzed and the generation upon which experiments were performed. For gene-edited lines, describe the editor used, the endogenous sequence targeted for editing, the targeting guide RNA sequence (if applicable) and how the editor was applied.*
Authentication	*Describe any authentication procedures for each seed stock used or novel genotype generated. Describe any experiments used to assess the effect of a mutation and, where applicable, how potential secondary effects (e.g. second site T-DNA insertions, mosiacism, off-target gene editing) were examined.*
